# Effects of cryotherapy on function, pain intensity, swelling, and dorsiflexion range of motion in acute ankle sprain: Protocol for the FROST randomised controlled trial

**DOI:** 10.1371/journal.pone.0325456

**Published:** 2025-06-04

**Authors:** Julio Miranda, Hytalo Jesus Silva, Fabiane Gontijo Correa, Rafaela Figueiredo, Gabriel Fonseca, Victor Guilherme Oliveira, Sérgio Samuel Borba Fonseca Silva, Samuel Pereira Santos, Itayano Mendes Lamas, Frederico Ataíde, Anderson Santons, Sérgio Antunes Santos, Germano Coelho, Vinicius Cunha Oliveira

**Affiliations:** 1 Postgraduate Program in Rehabilitation and Functional Performance, Universidade Federal dos Vales do Jequitinhonha e Mucuri (UFVJM), Diamantina, Brazil; 2 Postgraduate Program in Health Sciences, Universidade Federal dos Vales do Jequitinhonha e Mucuri (UFVJM), Diamantina, Brazil; 3 Department of Physiotherapy, Universidade Federal dos Vales do Jequitinhonha e Mucuri (UFVJM), Diamantina, Brazil; 4 Nossa Senhora da Saúde Hospital, Diamantina, Brazil; 5 Medicine College, Universidade Federal dos Vales do Jequitinhonha e Mucuri (UFVJM), Diamantina, Brazil; Indiana University-Purdue University Indianapolis, UNITED STATES OF AMERICA

## Abstract

**Background:**

Cryotherapy is a low-cost treatment option recommended by clinical practice guidelines in acute ankle sprain. However, the current quality of the evidence that supports cryotherapy is still unclear. New high-quality randomised controlled trials are needed. The aim of the Freeze On Sprain Trial (FROST) is to investigate the effectiveness of cryotherapy on function, pain intensity, swelling and dorsiflexion range of motion in people with an acute episode of ankle sprain.

**Methods:**

This is a protocol of a two-arm randomised controlled trial. Eighty-two participants over 18 years old presenting grade I or II ankle sprain up to 72 hours from the episode will be randomly allocated to Ice Group (i.e., home prescription to apply cryotherapy on the injured ankle with elevation plus non-steroidal anti-inflammatory medication – NSAID) or No Ice Group (i.e., elevation plus NSAID). Our primary outcome is function measured by the Lower Extremity Functional Scale (LEFS) questionnaire. Our secondary outcomes are pain intensity (11-point numerical rating scale), swelling (figure-of-eight method) and dorsiflexion range of motion (goniometry). Participants will be assessed at baseline, 24 hours, 7–10 days, and 12 weeks after allocation. The analysis will follow the intention-to-treat principle using linear mixed models.

**Discussion:**

The results of this study will clarify the effectiveness of cryotherapy in acute ankle sprain for better clinical decision-making processes.

**Trial registration:**

Brazilian Clinical Trials Registry (REBEC) RBR-8v9gr9c.

## Introduction

Ankle sprains are a common health condition with a period prevalence of 12% in the general population and seven sprains per 1000 exposures in athletes [1]. A new episode of ankle sprain can lead to disability, an increased risk of chronic ankle instability and recurrence, as well as direct (e.g., healthcare services) and indirect costs (e.g., productivity loss) [2]. Thus, effective therapies are important in the decision-making processes for acute ankle sprain. Treatment options frequently used after an ankle sprain comprise of non-steroidal anti-inflammatory medication (NSAIDs) [3], cryotherapy [4,5], surgical treatment [6], joint mobilisation [7], kinesiotherapy [8,9], braces [10], and/or acupuncture [11], among others.

Cryotherapy is a low-cost and easily applied treatment employed for therapeutic purposes for centuries [12]. Its application in the management of acute musculoskeletal conditions became popular mainly through the use of ice packs [13,14], and it was established in clinical practice as the standard of care through the RICE protocol (Rest, Ice, Compression, and Elevation), proposed by Mirkin in 1978 [15]. Nevertheless, the evidence regarding its effectiveness remains inconclusive [16–18], which has led to ongoing debates in the literature in recent years [19–21]. Clinical practice guidelines offer divergent recommendations for different types of acute musculoskeletal injuries, with some advocating its use [22,23], while others do not include it in their recommendations [24,25]. Consequently, there remains no consensus regarding the use of cryotherapy in acute injuries, leading many clinicians to base their prescriptions on theoretical mechanisms of action.

Preclinical research suggests that cryotherapy’s mechanisms of action are related to inflammation control [26,27] and local analgesia through reduced nociceptive conduction [28]. Cryotherapy may decrease nerve conduction velocity and reduce muscle spasms triggered by spinal reflexes after trauma [29], which could help modulate pain signalling and perception. Additionally, it may stimulate thermoreceptors, potentially inhibiting nociceptive signal processing in the central nervous system and increasing the pain threshold [28]. By lowering the local temperature, cryotherapy can reduce metabolic demand, which may help prevent swelling and secondary injury due to post-traumatic hypoxia, ultimately protecting against cell death [30]. Furthermore, local cold-induced vasoconstriction may reduce swelling by decreasing blood flow and vascular permeability [31,32]. Recent findings support this mechanism, showing that cold-water immersion significantly reduces femoral artery and cutaneous blood flow, likely due to skin cooling activating thermo-nociceptors and increasing sympathetic activity [33]. However, although findings from clinical research provide hypotheses regarding the mechanisms of action of cryotherapy, clinical decision-making should be guided by high-quality studies that assess patient-centred outcomes (e.g., function and pain intensity) rather than relying on surrogate measures [34].

A previous systematic review [35] investigated the effectiveness of cryotherapy in acute ankle sprain and found a lack of clinical research to support the efficacy of cryotherapy in clinical outcomes, raising the importance of new high-quality randomised controlled trials. Therefore, the Freeze On Sprain Trial (FROST) aims to investigate whether cryotherapy enhances the effects of NSAIDS, rest and elevation on function, pain intensity, swelling and dorsiflexion range of motion in people with an acute episode of ankle sprain. This randomised controlled trial may play an essential role as part of an informed decision-making process.

## Study objectives

The primary aim of this trial is to investigate whether cryotherapy enhances the effects of NSAIDs, limb elevation and rest on function in people with acute ankle sprain. The secondary aim is to investigate whether cryotherapy enhances the effects of NSAIDs, limb elevation and rest on pain intensity, swelling and dorsiflexion range of motion in people with acute ankle sprain.

## Methods

### Elaboration of the protocol

This protocol was developed according to the Standard Protocol Items: Recommendations for Interventional Trials (SPIRIT) guideline [36] (S1 File), and the results will be reported according to the Consolidated Standards of Reporting Trials (CONSORT) statement when the study is completed [37].

### Study design

This is a prospective, parallel-group, two-arm, superiority randomised controlled trial with a 1:1 allocation ratio.

### Trial Status

Date of the first enrolment: 05 Mar 2023; Expected date when recruitment will be completed: 05 Sep 2027; Expected date when data collection will be completed: 05 Dec 2027; Expected date when results will be completed: 01 Mar 2028.

### Research ethics approval

This study was approved by the Research Ethics Committee of the Federal University of Vales do Jequitinhonha and Mucuri (UFVJM) (CAAE: 58542222.2.0000.5108). The study protocol was approved by the institutional review board and is available in the S2 File. This study will be conducted in accordance with the Declaration of Helsinki.

### Informed consent

The physicians (IML and SPS) will obtain written informed consent forms from participants following eligibility screening and approval for inclusion in the study. Before obtaining consent, participants will be provided with a clear and detailed information sheet outlining the study’s purpose, procedures, potential risks, benefits, and their rights as participants. In addition to the information sheet, the physicians will explain the study to the participants and address any questions or concerns to ensure complete understanding.

As all participants will be adults with the legal and mental capacity to make decisions, assent will not be required. Each participant will provide their formal agreement to participate through a signed written informed consent form. The signed informed consent form will be stored securely, and participants will be provided with a copy of the form for their records. The written consent form will be documented, and the research team will address any queries or concerns raised by participants during the study. This trial does not involve the collection of biological specimens for storage.

### Settings and eligibility criteria

Study participants will be people aged 18–60 seeking care for a new episode of acute ankle sprain at the emergency service of the Nossa Senhora da Saúde Hospital in Diamantina, Brazil.

The inclusion criteria will be:

- People aged from 18 to 60 years old;
- Clinical diagnosis of grade I or II ankle sprains by a trained clinician suggesting an incomplete ligament rupture [38];
- Duration up to 72 hours from the new episode of ankle sprain to the day of the medical appointment;
- Bone fractures excluded by the Ottawa ankle rules and radiography [39].

The exclusion criteria will be as follows:

- Grade III (severe) ankle sprain, suggesting complete ligament injury, determined by a clear positive test of the anterior drawer and/or inversion stress test, accompanied by severe swelling, haemorrhage, high level of pain on palpation, in addition to total loss of the ability to support weight on the foot and of the dorsiflexion range of motion [38].
- Open injuries that contraindicate the application of ice (e.g., any degree of vascular ulcer);
- Application of any cryotherapy more than once since the episode, before the allocation process;
- Any condition contraindicative of the application of ice (e.g., Raynaud’s syndrome) or any other intervention prescribed in the trial.

### Intervention

The intervention will be reported according to the Template for Intervention Description and Replication (TIDieR) checklist and guide [40] (S3 File). Patients will be advised not to participate in any rehabilitation programme until the end of the trial (i.e., 12 weeks after allocation).

#### Ice Group.

Participants allocated to the ‘Ice Group’ will receive a home prescription to submerge the ankle in a bucket of ice and water until covering the area of swelling and pain, sitting with 90° knee flexion in the affected limb for up to three times per day, for 20 minutes, during seven days. In addition to cryotherapy, ankle elevation above the chest level at least three times a day for seven days, NSAID (i.e., nimesulide 100 mg, two times a day, for five days) and medical advice to rest for three days are prescribed [15,19,26,41].

The cryotherapy and ankle elevation instructions will be provided in a detailed booklet that participants will take home. These instructions will be reinforced, and any questions addressed during daily phone calls and/or text messages by physiotherapists (FGC and RF), who will also motivate participants to record applications in intervention diaries to assess adherence and adverse effects (S4 File). When participants do not adhere to immersion, alternative ice packs will be prescribed in the same dosage.

#### No Ice Group.

Participants allocated to the ‘No Ice Group’ will receive the same interventions as the ‘Ice Group’ but without ice. They will be provided with a booklet containing instructions on ankle elevation above the chest level, performed at least three times a day for seven days, and on avoiding any form of cryotherapy. The prescription will also include NSAID (i.e., nimesulide 100 mg, two times a day, for five days) and medical advice to rest for three days. Daily phone calls and/or text messages by physiotherapists (FGC and RF) will reinforce these instructions, address questions, motivate adherence, and encourage participants to record in the intervention diaries.

#### Criteria for discontinuing or modifying allocated interventions.

Participants experiencing adverse effects related to cryotherapy or any other intervention in the study will have their intervention discontinued immediately, and the adverse events will be documented. Additionally, the intervention will be discontinued if the participant withdraws consent. Even if the intervention is discontinued, participants will be encouraged to remain in the trial for follow-up assessments to ensure complete data collection and minimise missing data. Under no circumstances will participants be switched to a different group after randomisation.

#### Concomitant care and interventions.

Participants will be advised not to use any other intervention or treatment until the short-term follow-up assessment. If a participant initiates another intervention or begins a different treatment plan before the final follow-up, they will be instructed to report these details to the research team. All additional interventions will be documented to ensure transparency and to assess their potential impact on the study outcomes. This approach aims to maintain the comparability of study groups and minimise the risk of cointervention bias.

### Outcome measures

#### Primary outcomes.

The primary outcome will be lower extremity function, measured with the 0–80 Lower Extremity Functional Scale (LEFS), a questionnaire validated by Binkley et al. (1999) [42] for assessing lower limb function. The LEFS has a score range of 0–80, with higher scores indicating better functional status and a Minimum Clinically Important Difference (MCID) of 9 points. Participants will be assessed at baseline, 7–10 days, and 12 weeks after allocation.

#### Secondary outcomes.

The average pain intensity in 24 hours will be measured with an 11-point Numerical Rating Scale (0–10 NRS), with higher scores meaning worse pain intensity [43] and MCID of 1.3 points [44]. Participants will be assessed at baseline, 24 hours, 7–10 days, and 12 weeks after allocation.

The swelling will be measured using the figure-of-eight method, which consists of circumference using a tape measure of the areas with the highest concentration of ankle swelling (the region of the anterior talofibular, calcaneofibular and anterior tibiofibular ligaments). The measurement is made by positioning the starting point (0) of the tape measure over the midpoint between the articular projection of the tibialis anterior tendon and the lateral malleolus, directing the tape to the centre of the medial longitudinal arch of the foot, over the navicular bone, passing through the base of the fifth metatarsal and crossing the upper face of the midfoot towards the lower point of the medial malleolus, passing through the calcaneal tendon, lower point of the lateral malleolus, until finding the zero point of the measure tape [45]. See Reis et al. (2004) [46] for further details on the measurement technique. The Minimum Detectable Change (MDC) is 0.96 centimetres (cm) [47]. Participants will be assessed at baseline, 7–10 days, and 12 weeks after allocation.

The dorsiflexion range of motion will be assessed by two measurements of active ankle goniometry with the patient in the prone position (knee extension and 90° of knee flexion to reduce the influence of the gastrocnemius). The ankle will be positioned at 0° of movement in the sagittal plane, placing the goniometer axis over the calcaneus, just inferior to the lateral malleolus, with the fixed arm aligned with the calcaneal tendon and the mobile arm with the second metatarsal. A digital goniometer will be used to measure. The participant will be instructed to perform as much dorsiflexion as possible [48–50]. The MDC for this measurement is 6 degrees (^o^) [32]. Participants will be assessed at baseline, 7–10 days, and 12 weeks after allocation.

#### Harm outcomes.

Adverse events will be defined as any untoward medical occurrence in a participant during the trial, regardless of a known causal relationship with the intervention. It will be recorded from the moment participants provide a written informed consent form and are enrolled in the study. Events occurring before the initiation of the cryotherapy intervention will be recorded as unrelated to the study procedure. The most common adverse effects associated with cryotherapy include, but are not limited to, superficial skin burns, itching or allergic reactions, cold-induced peroneal nerve palsy, or other symptoms. Participants will be encouraged to self-report adverse effects daily through the intervention diaries, which include structured fields to capture specific events (S4 File). Additionally, participants will be able to report adverse effects directly to the physiotherapist responsible, ensuring timely documentation and management of any concerns.

Adverse events will be classified into serious adverse events, which include life-threatening conditions, severe or permanent disability, or hospitalisation, and non-serious adverse events, which may indicate intervention-related discomfort or complications without posing life-threatening risks or causing permanent disability. All adverse events during the trial period will be reported to the local Institutional Research Ethics Committee as soon as they are identified and documented with detailed descriptions. Data will be analysed descriptively, summarising the frequency and severity of reported events across groups. Any trends will be evaluated for potential causality with the intervention.

### Procedures and participant timeline

Individuals presenting to the emergency service with ankle sprains will be assessed for eligibility by physician members of the study (SPS and IML). These physicians will explain the study and provide the written informed consent form. All eligible participants must review and sign this document before participation. The written consent form will also inform participants that they may choose whether their data can be used if they withdraw from the trial. Additionally, they will be asked to authorise the research team to share relevant data with affiliated universities or regulatory authorities if needed. This trial does not involve the collection of biological specimens for storage.

**Fig 1:**
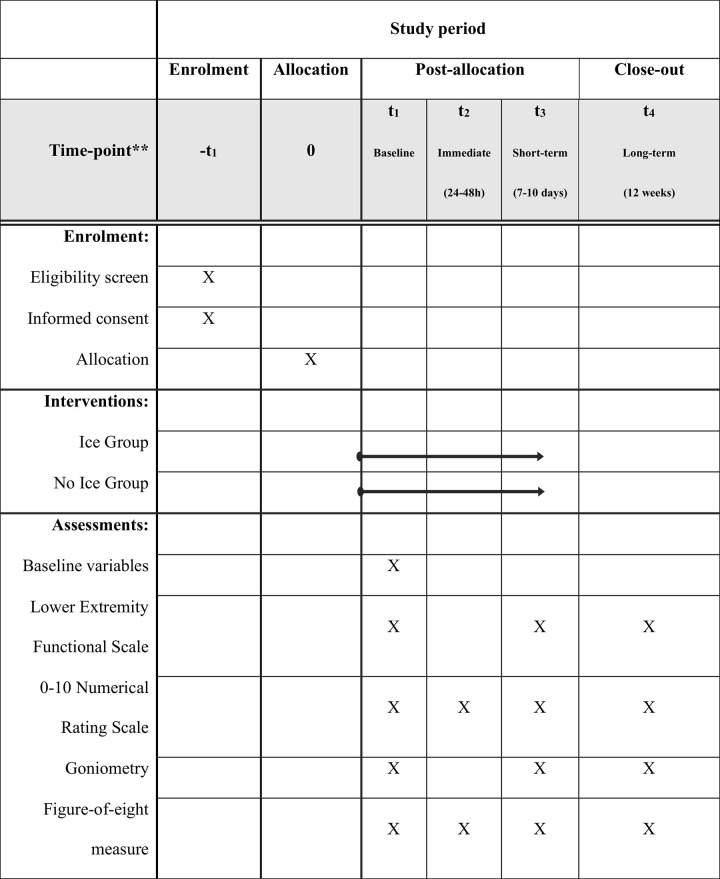
Schematic diagram of the procedures and participant timeline. t_1_ – Baseline assessment; t_2–_24-48 assessment (immediate effects); t_3_–7-10 days’ assessment (short-term; t_4_–12 weeks’ assessment (long-term).

Baseline assessment, conducted by SPS and IML, will include age, body mass index, sex, dominant limb, history of previous ankle sprains, ability to bear weight on the affected ankle (Yes/No), and comorbidities. The degree of injury will be classified according to the Birrer et al. (1999) [38] classification. Outcomes of interest (function, pain intensity, swelling, and dorsiflexion range of motion) will be assessed at baseline by SPS and IML and reassessed at the following time points by JM: short-term (7–10 days after allocation) and long-term (12 weeks after allocation). Baseline data collection will take place at a hospital emergency department, where patients are initially recruited. Additionally, the immediate effects of cryotherapy on pain intensity will be evaluated 24–48 hours after baseline (also by JM) through remote contact via telephone. Short-term and long-term assessments will be conducted in a private evaluation room at the university’s physical therapy department to ensure confidentiality, participant comfort, and data quality. A schematic diagram is available in Fig 1.

### Sample calculation

#### Function (LEFS) – primary outcome.

The sample calculation was performed considering the MCID value of 9 points for measuring the primary outcome [42] and a standard deviation of ± 12.85 based on a previous study [4]. A sample of 82 participants (41 per group) is necessary for a minimum detection of the effect size, considering a statistical power of 80%, α of 5% and a dropout rate of 20%.

#### Pain intensity (0–10 NRS) – secondary outcome.

The sample calculation was performed considering the MCID value of 1.3 points [44] and a standard deviation of ± 0.8 based on a previous study [51]. A sample of 16 participants (8 per group) is necessary for a minimum detection of the effect size, considering a statistical power of 80%, α of 5% and a dropout rate of 20%.

#### Swelling (figure-of-eight method) – secondary outcome.

The sample calculation was performed considering a difference of 3.4 cm and a standard deviation of ± 3.8 based on a previous study [47]. A sample of 50 participants (25 per group) is necessary for a minimum detection of the effect size, considering a statistical power of 80%, α of 5% and a dropout rate of 20%.

#### Dorsiflexion range of motion (Goniometry) – secondary outcome.

The sample calculation was performed considering a difference of 6° and a standard deviation of ± 6.8 based on a previous study [51]. A sample of 52 participants (26 per group) is necessary for a minimum detection of the effect size, considering a statistical power of 80%, α of 5% and a dropout rate of 20%.

### Recruitment

Individuals seeking care for acute ankle sprains at the Nossa Senhora da Saúde Hospital emergency department will be assessed for eligibility. Physicians will explain the trial to potential participants and obtain a written informed consent form. If eligible and willing to participate, these individuals will be referred to the researchers for further procedures. Social media and posters will also inform the population about the ongoing trial.

### Randomisation and allocation

The randomisation sequence for the experimental and control groups, with a 1:1 allocation ratio, will be computer-generated using Microsoft Excel® by a researcher not involved in recruitment or assignment (HJS). The sequence will be generated in blocks of 4, 6, and 8 in a random order. After agreeing to participate and signing the written informed consent form, the participant will receive a sequentially numbered, opaque, sealed envelope from the physician. Participants will be instructed to open the envelope after leaving the consultation room. The physicians, who are part of the research team but will not be involved in data collection during follow-ups, will remain blinded to group allocation. All procedures will follow methods previously described in the literature [52].

### Blinding

The statistician will be blinded to the allocation of the participants. The data will be encoded unidentifiably and will not contain any information that can raise suspicions about the allocation of the participants. Additionally, the assessor will be blinded to group allocation. However, participant blinding is not possible due to the nature of the intervention and the comparator group. The circumstances in which unblinding is allowed will be if the application of ice or any of the interventions applied in this study generate severe side effects or damage that requires medical assistance.

### Data collection methods

In this trial, data collection will be conducted initially using individual paper forms, with all recorded data subsequently transferred to an online spreadsheet (Google Sheets) for organisation and analysis. Weekly team meetings to address data inconsistencies, cross-verification of transferred data from paper forms to the digital database, and periodic audits of the online spreadsheet to identify and correct potential errors

The measurement instruments used in this study are reported in the literature as valid and reliable tools for assessing the outcomes. The LEFS demonstrated excellent test-retest reliability, with an intraclass correlation coefficient (ICC) of 0.94 (95% CI: 0.89 to 0.99) and a standard error of measurement (SEM) of ±3.9 [42]. The 0–10 NRS showed an ICC of 0.95 (95% CI: 0.93–0.96) and an SEM of 0.48 [53]. The figure-of-eight method exhibited an ICC of 0.99 (p < 0.05) [47], and goniometry for ankle dorsiflexion range of motion showed an intra-rater reliability of ICC = 0.91 and SEM = 2° [48]. No confidence interval values were reported for these two outcome measurements.

All researchers involved in baseline data collection and outcome assessments underwent standardised training to ensure measurement consistency and adherence to study protocols. This training emphasised the uniform application of assessment procedures, promoting data accuracy and reliability. An internal pilot evaluation of the intra- and inter-examiner reliability of the tools was conducted to ensure internal validation. Data for the intraclass correlation coefficient (ICC) and its 95% confidence interval (95% CI) were collected on two separate measurement occasions, with an interval of one week. We recruited six individuals of both sexes (three males and three females) and collected measurements from both lower limbs (n = 12). For goniometry, the intra-examiner reliability showed an ICC of 0.95 (95% CI: 0.80 to 0.98), and the inter-examiner reliability showed an ICC of 0.93 (95% CI: 0.83 to 0.98). For the figure-of-eight technique, the intra-examiner reliability showed an ICC of 0.97 (95% CI: 0.84 to 0.99), and the inter-examiner reliability showed an ICC of 0.96 (95% CI: 0.88 to 0.99).

### Retention

Strategies to reduce attrition and maximise data collection completeness will include daily contact with participants through phone calls or messages, appointment scheduling during enrolment, and retention monitoring. The reasons for the withdrawal will be collected.

To minimise the risk of missing data, participants who withdraw from the study or are unable to attend the evaluation site due to unforeseen circumstances, such as relocation, will be provided with the patient-reported outcome measures (PROMs), including the LEFS and 0–10 NRS, via online forms (Google Forms, a web-based survey platform). This approach aims to maximise data retention and ensure comprehensive outcome assessment.

#### Data management.

All study-related information will be securely stored in locked file cabinets in restricted-access areas at the university. A coded ID number will identify all reports, data collection, processing, and administrative forms, ensuring participant confidentiality. Participant information will not be released outside the study without explicit permission.

Data collection will begin with individual paper forms, which will be locally entered into Google Sheets for electronic storage. Google Sheets is a cloud-based platform employing data transmission and storage encryption, ensuring secure access and protection against unauthorised breaches. Access will be restricted to authorised researchers through password-protected Google accounts with two-factor authentication. Additionally, Google Sheets provides an automatic version history, allowing researchers to track, review, and restore modifications as needed. The platform also features automated backup and recovery mechanisms, minimising the risk of data loss or accidental modifications [54].

To ensure data quality and minimise errors, we will implement double data entry, where all data entries will be independently performed twice by separate individuals to identify and correct discrepancies [55]. The three steps of the method after initial data entry are re-entry, error detection, and error correction. In the re-entry step, a different person (GF) will enter the same data on an occasion separate from the first data entry. The error detection step is an automated comparison of the two versions of the keyed data. Discrepancies are detected in this step. In the error correction step, discrepancies and data entry errors are checked and corrected. Automated range checks will be employed to verify that data values fall within acceptable and expected ranges, flagging potential errors for review. Additionally, a verification process will involve independent source document verification of a subset of the data by a person not involved in the study, comparing electronic entries with the original paper forms to detect missing or erroneous values.

A dedicated data management team will monitor the research data weekly, and any identified errors will be promptly addressed. Informed consent forms and other essential documents will be digitised and securely stored on password-protected computers at the university, used exclusively by the lead researcher (VCO). Research files will also be uploaded to secure online spreadsheets linked to the research group’s lead investigator (VCO), ensuring the data are appropriately backed up and accessible only to authorised team members (VCO, GF, SSBFS, RF, and VGO).

Weekly meetings will review the clinical trial’s progress, address emerging issues, and ensure consistent protocol adherence. Stringent internal checks will be conducted by authorised team members (VCO, GF, SSBFS, RF, and VGO) to maintain data integrity. All participant records will be securely maintained for three years following the completion of the study, ensuring compliance with ethical and legal standards for research data retention.

### Plan of analysis of treatment effects

The statistical analysis will follow the intention-to-treat principle. The analysis will be conducted by a statistician (HJS), who will not be involved in recruitment or data collection. Between-group differences and their 95% confidence intervals (95% CI) will be analysed using linear mixed models (LMM) with random intercepts and fixed coefficients. Participant ID will be considered a random effect, while group (experimental vs. control), time (baseline, short- and long-term), and the group-by-time interaction will be treated as fixed effects. Post-hoc Bonferroni tests will be applied to adjust for multiple comparisons.

The assumptions of the LMM will be assessed to ensure the validity of the results. The normality of residuals will be tested using the Kolmogorov-Smirnov test, and homoscedasticity will be evaluated using Levene’s test. The independence of residuals and linearity between continuous predictors and the dependent variable will be assessed using scatterplots, with the Durbin-Watson test being used to evaluate residual independence further. Multicollinearity among independent variables will be assessed using the Variance Inflation Factor, with values exceeding five indicating potential concerns.

Different covariance structures (compound symmetry, autoregressive (AR(1)), and unstructured) will be tested to identify the most appropriate model for each outcome. For self-reported outcome measures (LEFS and 0–10 NRS), compound symmetry is expected to be suitable, given the consistency of responses within individuals over time. For ankle dorsiflexion range of motion and swelling outcomes, AR(1) will be initially tested, followed by an unstructured model, as it accounts for variability introduced by different assessors and time. The final covariance structure will be selected based on the lowest Akaike Information Criterion and Bayesian Information Criterion values.

In the case of non-parametric distributions, differences between groups will be analysed using Generalised Linear Models. The level of statistical significance will be set at p-value < 0.05. Effect sizes will be interpreted based on the corresponding 95% CIs of the difference between groups with the minimum clinically important differences (MCIDs) of the outcomes. Cohen’s d will also be calculated to quantify the magnitude of treatment effects, with thresholds for interpretation as follows: small effect (d = 0.2), medium effect (d = 0.5), and large effect (d = 0.8). All statistical analyses will be performed using Stata software, version 17.0 (StataCorp LLC, College Station, USA).

### Additional analysis

Subgroup analyses to explore different sample characteristics, such as classification of the ankle sprain (grade 1 or 2), the presence or absence of comorbidities, adherence to the prescribed treatment (based on the intervention diaries), and whether participants used concomitant interventions outside of those prescribed by the research team can be performed if deemed necessary.

The primary analysis will be conducted without adjustments for baseline covariates, as randomisation is expected to distribute these characteristics evenly between groups. In case of a significant imbalance in baseline characteristics in critical prognostic factors, group differences will be analysed, considering these factors as fixed-effects covariates to adjust their influence on the statistical model.

### Imputation procedure for missing data

To handle missing data, we will classify as Missing Not at Random (MNAR) when dropouts are due to lack of efficacy, adverse effects or lack of efficacy and Missing Completely at Random (MCAR) when the loss of follow-up does not depend on observed or unobserved measurements (e.g., patient moving to another city for non-health reasons). We plan to use the linear mixed models to handle missing data due to MCAR and multiple imputations when we consider missing as MNAR [56]. Multiple imputations will be performed using chained equations (MICE), restricting imputations to outcome variables with missing data. A total of ten imputed datasets will be generated, and the results will be pooled using Rubin’s rules. The pseudorandom number generator will be pre-specified as seed 618151920 to ensure transparency and reproducibility.

### Sensitivity analysis

To assess the robustness of the findings, sensitivity analyses will be performed considering factors that may influence the results. Outliers will be identified using boxplots, and analyses will be conducted with and without these observations to evaluate their impact on the estimated effects. An additional per-protocol analysis will be compared to the primary intention-to-treat analysis to address protocol deviations. In case of baseline imbalances, the model will be tested with and without the baseline variable that exhibits imbalance, including it as a covariate. Given that different covariance structures were tested to select the most appropriate for each outcome, sensitivity analyses will be performed to assess the robustness of the final choice by re-running models with alternative structures (e.g., compound symmetry, AR(1), unstructured) and comparing the estimated effects. Finally, to evaluate the impact of potential violations of LMM assumptions, analyses will be conducted using Generalised Linear Models with alternative distributions suitable for the data (e.g., Gamma distribution for positively skewed data).

Sensitivity analyses to test the robustness of the imputation will be made using the best or worst-case imputation, i.e., assigning the worst possible value of the outcome to dropouts for a negative reason (treatment failure) and the best possible value to positive dropouts (cures) to evaluate the range of uncertainty due to missing data [56,57].

### Planning for supervision and monitoring

This trial does not include a formal data monitoring committee. According to the SPIRIT guidelines [36], the decision to establish a data monitoring committee depends on local standards, the complexity and duration of the trial, and the level of associated risks. Trials with a short duration or minimal risks may not require a formal committee. Our study falls into this category, as it involves low-risk interventions and the minimal likelihood of severe life-threatening adverse events, and it is of relatively short duration. These factors, combined with the ethical approval from the Institutional Research Ethics Committee, support the decision not to establish a data monitoring committee for this trial.

Instead, data monitoring responsibilities will be managed internally by designated research team members (VCO, GF, SSBFS, RF and VGO). These team members will oversee data collection, storage, and management processes to ensure the integrity and quality of the trial data. Weekly team meetings will discuss trial progress, identify and resolve any issues, and maintain adherence to the established protocol.

### Interim analyses

There are no interim analyses planned.

### Auditing

This study does not include independent audits, as none were requested by the Institutional Research Ethics Committee nor deemed necessary due to the nature of the trial, its limited risks, and the absence of involvement from industry sponsors. The integrity of the study will be ensured through continuous monitoring of data collection and the implementation of stringent quality control processes, as outlined in previous sections. Additionally, the data management team will monitor the data weekly (VCO, GF, SSBFS, RF, and VGO) and will perform checks to ensure the accuracy and completeness of the data, as specified in the protocol.

Although formal auditing is not part of the protocol, daily monitoring practices and ongoing review of information, which ensure compliance with ethical and regulatory standards, will be overseen by the principal investigator (VCO), who will ensure that the research team promptly addresses any inconsistencies or procedural deviations.

### Protocol amendments and revision chronology

In case of any protocol amendments, a detailed plan will be followed to ensure proper communication and documentation. First, any proposed changes to the protocol will be promptly communicated to the ethics committee. After obtaining their approval, the principal investigator will notify all researchers about the amendment. A revised protocol version will be fully documented using a breach report form. This form will detail the nature of the deviation, its justification, and any corrective actions taken. Additionally, any modifications to the protocol will be updated in the clinical trial registry to ensure transparency and compliance with regulatory standards. The amendments made up to this point are summarised in Table 1.

**Table 1:** Protocol amendments.

Date	Version
30 Aug 2022:	Original
05 Mar 2023	Amendment 01: Due to licence issues, there is a change in the “Analysis of treatment effects” section regarding the statistical program. We have switched from SPSS to Stata v.17.
20 Jan 2025	Amendment 02: Updates to the protocol based on peer reviewers’ suggestions. The dates for recruitment, data collection, analysis, and trial completion have been updated based on the trial’s progress.

### Medical team

The medical team will comprise physicians in the Nossa Senhora da Saúde Hospital emergency department, in Diamantina, Minas Gerais, Brazil. They are all members of the study team. Physicians GC, SAS, FA, and AS will offer institutional support to represent the hospital throughout the trial and be responsible for the participants’ safety. SPS and IML will be responsible for participant recruitment and baseline assessments.

### Access to data

During the study, the data monitoring team (VCO, GF, SSBFS, RF, and VGO) will have full access to the dataset to ensure the quality and consistency of the collected information. Following data collection, JM and HJS will have full access to the dataset for subsequent analyses and reporting. Other research team members may request access to the dataset, subject to justification and approval by the principal investigator (VCO) and in compliance with established ethical and regulatory confidentiality standards.

### Compensation for trial participation

If, after completing all follow-ups and concluding their participation in the study, the participant experiences residual symptoms, they will be invited to receive treatment at the school clinic in the physiotherapy department of the Federal University of Vales do Jequitinhonha and Mucuri (UFVJM).

### Data sharing and dissemination policy

The de-identified minimal data set required to reproduce all statistical analyses, including the raw values used to calculate means, standard deviations, and other reported statistics, will be made available via Research Square, where the preprint is currently hosted under a CC BY 4.0 licence [58] or from the corresponding author upon reasonable request after publication of the completed trial in a scientific journal. A robust anonymisation process will be adopted, removing direct identifiers such as names, specific dates, and locations.

The results will be disseminated regardless of the results via scientific journal publications, conference abstracts, and the institutional repository. There are no restrictions imposed by the funders or the institution on publishing the results, irrespective of the direction or magnitude of the effect.

### Authorship eligibility statement

Authorship will be granted to individuals who make substantive contributions to the design and data collection, which involves significantly recruiting participants, administering interventions, acquiring study data, data analysis, and drafting or revising the manuscript. All authors will approve the final version of the manuscript before submission. Individuals who do not meet these criteria will not be listed as authors but will be acknowledged for their contributions where appropriate. No ghost authorship, where eligible contributors are not disclosed, and guest authorship, where individuals without significant contributions are included, will be accepted. Ultimately, we have no intention of hiring professional writers.

## Discussion

This article presents the study protocol for the FROST randomised controlled trial, which aims to investigate the effects of cryotherapy on function, pain intensity, swelling, and ankle dorsiflexion range of motion in individuals with acute ankle sprains. A previous systematic review [35] highlighted the scarcity of high-quality randomised controlled trials that investigate the effects of cryotherapy using appropriate comparators to isolate its effects on clinical outcomes. This trial seeks to fill this gap using a robust methodological design, including a comparison group with no ice component.

Surrogate endpoints, typically biomarkers or intermediate measures, are often employed as indirect substitutes for clinical outcomes of actual relevance to patients [59,60]. Although their use may enhance trial efficiency by permitting shorter follow-up periods and smaller sample sizes [60], surrogate outcomes do not directly capture patients’ experiences or recovery trajectories and may introduce greater uncertainty regarding the actual clinical benefits of an intervention [59]. Conversely, target outcomes directly reflect how an individual feels, functions, or survives, particularly when self-reported by the patients [60]. Recognising this, the FROST trial was designed to evaluate outcomes of meaningful relevance to patients and clinicians.

Nonetheless, this study has some limitations. Although the design isolates cryotherapy as an intervention, the control group does not include a placebo alternative for ice application, which may introduce a placebo effect bias. Additionally, blinding patients and therapists is not feasible due to the nature of the intervention, which may lead to bias, such as performance bias. To mitigate this risk, standardised intervention protocols will be implemented to ensure consistency in treatment delivery, and participants will receive uniform instructions to minimise variations in adherence. Moreover, all outcome assessors will remain blinded to group allocation, and participants will be instructed not to disclose their assigned group during assessments.

The sample size calculation was conducted separately for each outcome, and adjustments for multiple comparisons were not applied to the power calculation. We acknowledge the risk of a type I error (false positive), which could affect the interpretation of statistical significance. To address this, we will prioritise the interpretation of effect sizes and 95% confidence intervals, considering the MCID to assess the clinical relevance of our findings rather than relying solely on p-values.

In conclusion, the FROST trial represents a significant step forward in the knowledge of the effects of cryotherapy. The findings will contribute to a comprehensive understanding of its clinical utility and help guide evidence-based decision-making in the management of acute ankle sprains.

### Administrative information

Note: the numbers in curly brackets in this protocol refer to SPIRIT checklist item numbers [17]. The order of the items has been modified to group similar items.

## Supporting information

S1 File SPIRIT guideline. (DOCX)

S2 File Study protocol approved by the ethics committee. (PDF)

S3 File TIDIER checklist. (DOCX)

S4 File Intervention diary. (DOCX)

S5 File WHO dataset. (DOCX)

## Abbreviations

CI: Confidence Interval
CONSORT: Consolidated Standards of Reporting Trials
ICC: Intra-class Correlation Coefficient
LEFS: Lower Extremity Functional Scale
LMM: Linear Mixed Models
MCAR: Missing Completely At Random
MCID: Minimum Clinically Important Difference
MDC: Minimal Detectable Change
MNAR: Missing Not At Random
NRS: Numerical Rating Scale
NSAID: Non-steroidal anti-inflammatory drugs
PROM: Patient-reported outcome measure
REBEC: Registro Brasileiro de Ensaios Clínicos
SEM: Standard Error Measures
SPIRIT: Standard Protocol Items: Recommendations for Interventional Trials
UFVJM: Universidade Federal dos Vales do Jequitinhonha e Mucuri
