# Supplementary material for: Effects of cryotherapy on function, pain intensity, swelling, and dorsiflexion range of motion in acute ankle sprain: Protocol for the FROST randomised controlled trial
Source: PLoS One. 2025 Jun 4;20(6):e0325456. doi: 10.1371/journal.pone.0325456 (PMC12136321; doi:10.1371/journal.pone.0325456)
Supplement: S2 File — (PDF) [file pone.0325456.s002.pdf]

UNIVERSIDADE FEDERAL DOS VALES DO JEQUITINHONHA E MUCURI
PROGRAMA DE PÓS GRADUAÇÃO EM REABILITAÇÃO E DESEMPENHO FUNCIONAL

Júlio Pascoal de Miranda

EFETIVIDADE DA CRIOTERAPIA NA INTENSIDADE DA DOR, AMPLITUDE DE MOVIMENTO DE DORSIFLEXÃO, EDEMA E FUNÇÃO NA ENTORSE DE TORNOZELO AGUDA: UM ENSAIO CONTROLADO ALEATORIZADO – O ESTUDO FROST

Vigência do projeto: 14/03/2022 a 14/03/2028

Orientador: Prof. Dr. Vinícius Cunha de Oliveira

Instituição: Universidade Federal dos Vales do Jequitinhonha e Mucuri

Diamantina
2022

Júlio Pascoal de Miranda

EFETIVIDADE DA CRIOTERAPIA NA INTENSIDADE DA DOR, AMPLITUDE DE MOVIMENTO DE DORSIFLEXÃO, EDEMA E FUNÇÃO NA ENTORSE DE TORNOZELO AGUDA: UM ENSAIO CONTROLADO ALEATORIZADO – O ESTUDO FROST

Projeto de pesquisa apresentado ao curso *stricto sensu* do Programa de pós-graduação em Reabilitação e Desempenho Funcional da Universidade Federal dos Vales do Jequitinhonha e Mucuri, como parte dos requisitos exigidos para a conclusão do curso.

Orientador: Prof. Dr. Vinícius Cunha de Oliveira

Diamantina
2022

SUMÁRIO

1 INTRODUÇÃO 6

2 OBJETIVOS 7

2.1 Objetivo Geral 7

2.2 Objetivos Específicos 7

3 HIPÓTESES 7

3.1. Hipótese nula 7

3.2. Hipótese alternativa 7

4 JUSTIFICATIVA 8

5 MÉTODOS 8

5.1 Delineamento do estudo 8

5.2 Critérios de elegibilidade 8

5.2.1 Critérios de inclusão 8

5.2.2 Critérios de exclusão 9

5.3 Procedimentos 9

5.4 Medida de desfecho 10

5.5 Randomização 11

5.6 Cegamento 11

5.7 Intervenção 11

5.7.1 Grupo com gelo 12

5.7.2 Grupo sem gelo 12

6 ANÁLISE DOS DADOS 13

6.1. Cálculo amostral 13

6.2. Análises dos efeitos do tratamento 13

6.3. Dados Perdidos 13

7 RISCOS E BENEFÍCIOS 14

8 CRONOGRAMA 14

9 ORÇAMENTO 15

REFERÊNCIAS 17

Apêndice 1 – TCLE 20

Apêndice 2 – DIÁRIO DE INTERVENÇÃO 22

Anexo 1 – QUESTIONÁRIO LEFS (Adaptado de PEREIRA *et al.*, 2011) 23

Anexo 2 – TIDIER *CHECKLIST* 24

RESUMO

O objetivo deste ensaio controlado randomizado é investigar a efetividade da crioterapia na função, intensidade da dor, edema e amplitude de movimento de dorsiflexão em pessoas com um episódio agudo de entorse de tornozelo. Este é um protocolo de um ensaio controlado randomizado de dois braços. Pessoas maiores de 18 anos com diagnóstico clínico de entorse de tornozelo grau I ou II, e tempo de até 72 horas a partir do episódio da lesão, serão alocadas aleatoriamente no Grupo Gelo, que consiste em prescrição médica domiciliar para aplicação de bolsas de gelo no tornozelo com elevação, mais anti-inflamatório não esteroidal, ou Grupo Sem Gelo, que consiste na mesma prescrição médica do grupo experimental, mas sem gelo incluído. Nosso desfecho primário é função, mensurada pelo questionário Lower Extremity Functional Scale (LEFS). Nossos desfechos secundários são intensidade da dor (Escala Numérica de Dor, 0-10), edema (método da figura em oito) e amplitude de movimento de dorsiflexão (goniometria). Os acompanhamentos serão realizados no pós-tratamento (7 a 14 dias) e 12 semanas após a alocação. Um tamanho de amostra de 82 participantes será necessário para uma detecção mínima do tamanho do efeito do desfecho primário, com um poder de 80%, a de 5% e uma taxa de abandono esperada de 20%. As análises seguirão o princípio de intenção de tratar. Os efeitos do tratamento serão analisados por meio de Modelos Lineares Mistos. Os resultados deste estudo podem ajudar a esclarecer os efeitos da crioterapia no tratamento da entorse aguda de tornozelo e podem orientar clínicos na tomada de decisão.

1 INTRODUÇÃO

A entorse de tornozelo é uma condição comum na população em geral, com prevalência de 11,88%, e incidência de sete entorses a cada 1000 exposições em atletas, sendo a lesão tornozelo-pé relacionada ao esporte que mais leva à procura de serviços de saúde (GRIBBLE *et al*., 2016; DOHERTY *et al*., 2014; NABIAN *et al*., 2017). Após um novo episódio de entorse de tornozelo, há uma alta frequência de instabilidade crônica do tornozelo e recorrência (GRIBBLE *et al*., 2016). Logo, é uma lesão musculoesquelética que pode ocasionar custos diretos (gastos com consultas médicas e medicamentos) e indiretos (afastamento do trabalho e redução da produtividade) (GRIBBLE *et al*., 2016). Portanto, terapias eficazes para o tratamento de entorses de tornozelo agudas são cada vez mais procuradas pelos médicos.

As opções de tratamento frequentemente usadas após uma entorse de tornozelo incluem crioterapia (BLEAKLEY; MCDONOUGH; MACAULEY, 2006; VAN DIJK, 1999), tratamento cirúrgico (DOHERTY *et al*., 2017), mobilização articular (COSBY *et al.,* 2011), cinesioterapia (BLEAKLEY *et al*., 2010; CLELAND *et al*., 2013), órteses (BEYNNON *et al.,* 2006), acupuntura (DOHERTY *et al*., 2017), e outros. A crioterapia é uma opção de tratamento de baixo custo e fácil de usar, e tem sido recomendada por diretrizes de prática clínica para uso por profissionais de saúde em entorses de tornozelo agudas (VAN DIJK, 1999; VUURBERG *et al*., 2018). É considerada uma terapia potencialmente eficaz na fase inflamatória aguda devido aos achados de pesquisas pré-clínicas, que sugerem que a crioterapia pode controlar os processos inflamatórios, reduzindo a infiltração de macrófagos e os níveis de TNF-α, NF-κB, TGF-β e MMP-9 mRNA (NEMET *et al*., 2009; VIEIRA RAMOS *et al*., 2016); além de promover analgesia local pela diminuição da velocidade de condução nervosa (ALGAFLY; GEORGE, 2007), o que poderia levar à melhora dos desfechos clínicos.

No entanto, as evidências atuais de pesquisas clínicas que apoiam a crioterapia ainda não são claras. Uma revisão sistemática anterior (MIRANDA; SILVA; SILVA; MASCARENHAS; OLIVEIRA, 2021) investigou a eficácia da crioterapia em entorses de tornozelo agudas e constatou que a literatura carece de evidências para apoiar seu uso, levantando a importância de novos ensaios clínicos randomizados com baixa risco de viés e com grupos comparadores apropriados para isolar os efeitos das terapias (ou seja, placebo, simulação ou nenhuma intervenção) ou para investigar se a crioterapia aumenta os efeitos de outra intervenção (ou seja, crioterapia combinada com uma intervenção ativa em comparação com a mesma intervenção ativa sozinha).

Considerando isso, o objetivo deste ensaio controlado randomizado é investigar a eficácia da crioterapia na função, intensidade da dor, edema e amplitude de movimento de dorsiflexão em pessoas com um episódio agudo de entorse de tornozelo.

2 OBJETIVOS

2.1 Objetivo Geral

Investigar a eficácia da crioterapia na intensidade da dor, edema, amplitude de movimento de dorsiflexão (DFROM) e função em pessoas com entorse aguda de tornozelo.

2.2 Objetivos Específicos

- Investigar a eficácia da crioterapia na intensidade da dor em pessoas com entorse aguda de tornozelo por meio da escala numérica de dor**;**
- Investigar a eficácia da crioterapia no edema em pessoas com entorse aguda de tornozelo por meio da perimetria utilizando a tecnica da figura de 8**;**
- Investigar a eficácia da crioterapia no DFROM em pessoas com entorse aguda de tornozelo por meio da goniometria ativa do tornozelo;
- Investigar a eficácia da crioterapia na função em pessoas com entorse aguda de tornozelo por meio do questionário lower extremity functional scale (LEFS)
- Investigar os efeitos adversos da aplicação da crioterapia em pessoas com entorse aguda de tornozelo.

3 HIPÓTESES

3.1. Hipótese nula

Não há diferença entre os efeitos de tratamento entre o grupo com aplicação de gelo comparado com o grupo sem a aplicação de gelo na intensidade da dor, amplitude de movimento de dorsiflexão, edema e função em pessoas com entorse aguda de tornozelo.

3.2. Hipótese alternativa

Existe diferença entre os efeitos de tratamento entre o grupo com aplicação de gelo comparado com o grupo sem a aplicação de gelo na intensidade da dor, amplitude de movimento de dorsiflexão, edema e função em pessoas com entorse aguda de tornozelo.

4 JUSTIFICATIVA

O uso da crioterapia é uma prática difundida entre clínicos e pacientes no manejo da entorse aguda de tornozelo, além de ser recomendada por diretrizes de prática clínica. No entanto, as evidências sobre a efetividade dessa prática ainda são incertas. Uma revisão sistemática (MIRANDA *et al.*, 2021) encontrou que há evidências de baixo nível de certeza de que não há diferença de efeito ao utilizar ou não a crioterapia na entorse aguda de tornozelo, e que novos ensaios controlados aleatorizados (RCTs) com baixo risco de viés e grupo comparador adequado são necessários para esclarecer a efetividade desta prática.

5 MÉTODOS

5.1 Delineamento do estudo

Trata-se de um ensaio controlado aleatorizado prospectivo de dois braços. Um protocolo prévio foi elaborado seguindo as recomendações da diretriz SPIRIT (CHAN *et al*., 2013) e será registrado no Comitê de Ética em Pesquisa da Universidade Federal dos Vales do Jequitinhonha e Mucuri (UFVJM) e, em seguida, cadastrado no site REBEC (www.ensaiosclinicos.gov.br). Será reportado de acordo com a declaração CONSORT (ELDRIDGE *et al*., 2016). Todos os princípios éticos fornecidos pela Declaração de Helsinque (World Medical Association, 2013) serão seguidos por todos os membros desta pesquisa ao longo do estudo.

5.2 Amostra e critérios de elegibilidade

A AMOSTRA DESTE ESTUDO SERÁ POR CONVENIÊNCIA, composta por indivíduos com idade entre 18 a 60 anos, que sofrerem um episódio de entorse aguda de tornozelo, RECRUTADOS AO PROCURAREM O SERVIÇO DE EMERGÊNCIA DO HOSPITAL NOSSA SENHORA DA SAÚDE, localizado em Diamantina-MG, Brasil, com diagnóstico posteriormente confirmado por um médico ortopedista da instituição.

5.2.1 Critérios de inclusão

- Idade entre 18 a 60 anos;
- Diagnóstico clínico de entorse de tornozelo grau I ou II, indicando uma ruptura incompleta de ligamento de acordo com a classificação de BIRRER *et al.* (1999);
- Tempo de até no máximo 72 horas do episódio de lesão até o dia da consulta médica;

- Fratura óssea excluída por radiografia ou pelas regras de Ottawa para tornozelo (BACHMANN *et al.*, 2003).

5.2.2 Critérios de exclusão

- Entorse de tornozelo grau III (grave), indicando ruptura complete ligamentar, determinada por um claro teste positivo de gaveta anterior e/ou teste de estresse em inversão, acompanhado por edema grave, hemorragia, alto nível de dor a palpação, e perda total da DFROM e da capacidade de sustentar peso no pé (BIRRER *et al.*, 1999).
- Lesão aberta no local, que contraindique a aplicação de gelo;
- Ter aplicado alguma forma de crioterapia mais de uma vez desde o momento da lesão;
- Ter qualquer condição que contraindique a aplicação de gelo (por exemplo, síndrome de Reynaud), ou qualquer outra intervenção prescrita neste estudo.

5.3 Procedimentos

OS PACIENTES QUE PROCURAREM O SERVIÇO DE EMERGÊNCIA COM QUEIXA DE ENTORSE DE TORNOZELO SERÃO CONVIDADOS A PARTICIPAR DO ESTUDO. Todos àqueles elegíveis receberão informações sobre o estudo e deverão assinar um termo de consentimento livre esclarecido (TCLE) (Apêndice 1) antes da participação. A avaliação inicial E A REAVALIAÇÃO DO PARTICIPANTE OCORRERÁ NO HOSPITAL NOSSA SENHORA DA SAÚDE DENTRO DO CONSULTÓRIO MÉDICO USUAL, DE FORMA INDIVIDUALIZADA, PARA AMENIZAR QUALQUER CONSTRANGIMENTO NO MOMENTO DA COLETA ASSIM COMO RISCO DE DESCONFORTO PELA COLETA DOS DADOS. TANTO O LOCAL QUANTO AS INFORMAÇÕES DA AVALIAÇÃO INICIAL FORAM ADEQUADAS PARA SEREM O MAIS PRÓXIMO DA PRÁTICA CLÍNICA CORRIQUEIRA DOS MÉDICOS DA INSTITUIÇÃO, SENDO ASSIM, O PARTICIPANTE IRÁ DESPENDER APROXIMADAMENTE DO MESMO TEMPO QUE O COTIDIANO. OS DADOS COLETADOS incluirão idade, Índice de Massa Corporal (IMC), sexo, membro dominante, história de entorse de tornozelo anterior, capacidade de suportar peso no tornozelo afetado (Sim/Não) e se tem comorbidades. O grau de lesão será classificado de acordo com a classificação de BIRRER *et al.* (1999), que indica entorse de tornozelo grau I ou II, quando o diagnóstico clínico indica uma ruptura ligamentar incompleta, ou entorse de tornozelo Grau III (grave), indicando lesão ligamentar completa, determinado por um teste positivo claro da gaveta anterior e/ou teste de estresse em inversão, edema intenso,

hemorragia, alto índice de dor à palpação, além de perda total da capacidade de suportar peso no pé e da DFROM. Quando os indivíduos são classificados como entorse de tornozelo de Grau III, eles serão excluídos do estudo e receberão tratamento com base na imobilização do membro. TODOS os desfechos de interesse (função, intensidade da dor, edema e DFROM) serão coletados na linha de base e reavaliados nos seguintes momentos: curto prazo (ou seja, 7 a 14 dias após a alocação) NO HOSPITAL NOSSA SENHORA DA SAÚDE NO CONSULTÓRIO MÉDICO ONDE OCORREU A AVALIAÇÃO INICIAL; e longo prazo (ou seja, 12 semanas após a atribuição), EM UMA SALA DE AVALIAÇÃO DO PRÉDIO DE FISIOTERAPIA, NA UFVJM, CAMPUS JK. Além disso, iremos investigar os efeitos imediatos da crioterapia ESPECIFICAMENTE na intensidade da dor em um ponto de tempo entre 24h a 48h após o início do estudo, POR MEIO DE LIGAÇÃO TELEFÔNICA. NA REAVALIAÇÃO, O PARTICIPANTE PODERÁ DESPENDER DE UM TEMPO ENTRE 10 A 30 MINUTOS. EM TODOS AS ETAPAS DESTA PESQUISA, SERÃO SEGUIDAS TODAS AS NORMAS DE BIOSSEGURANÇA PARA A PREVENÇÃO DA DISSEMINAÇÃO DA COVID-19 JÁ ESTABELECIDAS NAS INSTITUIÇÕES PARTICIPANTES DESTE ESTUDO, COM TODOS OS PESQUISADORES FAZENDO O USO DE EQUIPAMENTOS DE PROTEÇÃO INDIVIDUAL NECESSÁRIOS. AS REAVALIAÇÕES NOS SEGUIMENTOS DE CURTO PRAZO (7 A 14 DIAS) SERÃO EXCLUSIVIDADE DOS PARTICIPANTES DESTE ESTUDO.

5.4 Medida de desfecho

Cada participante será avaliado para os seguintes resultados: Função, medida com o questionário 0-80 *Lower Extremity Functional Scale* (LEFS) (Anexo 1), com uma diferença clinicamente importante mínima (MCID) de 9 pontos (BINKLEY *et al.*, 1999); Intensidade da dor nas últimas 24 horas, medida com a Escala de Avaliação Numérica (NRS), que consiste em uma escala de 11 pontos, variando de 0, que corresponde a "Sem dor" a 10 "Pior dor imaginável" (KATZ; MELZACK, 1999) , com um MCID de 1,3 (95%IC = 1,0 a 1,5) (GALLAGHER; LIEBMAN; BIJUR, 2001); Edema, medido pelo método em oito, que consiste na perimetria com fita métrica nas áreas de maior concentração de edema do tornozelo (região dos ligamentos talofibular anterior, calcaneofibular e tibiofibular anterior). A medição é feita posicionando o ponto inicial (0) da fita métrica sobre o ponto médio entre a projeção articular do tendão tibial anterior e o maléolo lateral, direcionando a fita para o centro do arco longitudinal medial do pé, sobre o osso navicular, passando pela base do quinto metatarso e cruzando a face superior do mediopé em direção ao ponto inferior do maléolo medial, passando

pelo tendão do calcâneo, ponto inferior do maléolo lateral, até encontrar o ponto zero da fita métrica (MAWDSLEY; HOY; ERWIN, 2000), e tem uma alteração mínima detectável (MDC) de 0,96 cm (ROHNER-SPENGLER; MANNION; BABST, 2007); DFROM, medido pela goniometria ativa de tornozelo, posicionando o eixo do goniômetro aproximadamente 1,5 cm abaixo do maléolo lateral, com o braço fixo alinhado com a linha média lateral da perna e cabeça da fíbula, e o braço móvel alinhados ao quinto metatarso. O participante será instruído a realizar o máximo de dorsiflexão possível (YOUDAS *et al*., 2009). O MDC para esta medição foi determinado em 6° (YOUDAS *et al*., 2009). Será realizado treinamento dos avaliadores quanto à técnica em oito e goniometria do DFROM, seguido de estudo piloto para confiabilidade Intra e Inter examinadores. Os dados para o cálculo dos Coeficientes de Correlação Intraclasse (ICC) serão coletados em duas ocasiões distintas de medição, com intervalo de 1 semana. Prevê-se o recrutamento de seis a dez indivíduos de ambos os sexos para o estudo piloto, com coleta de medidas de ambos os membros inferiores. Todas as análises estatísticas serão realizadas usando o programa SPSS Statistics (v.22.0; IBM Corp, Armonk, NY).

5.5 Randomização

A sequência de randomização para os grupos experimental e controle, com proporção de alocação de 1:1, será gerada por um programa de computador por um dos pesquisadores que não estará envolvido no recrutamento dos participantes. A randomização será estratificada por idade em duas Stratas (‘18 a 40’ e ‘41 a 60 anos’). A sequência será gerada em blocos de 4, 6 e 8, em ordem aleatória. A atribuição será escondida em envelopes opacos selados numerados sequencialmente. Todo o procedimento será conduzido seguindo os métodos recomendados (DOIG; SIMPSONS, 2005).

5.6 Cegamento

O estatístico será cegado quanto à alocação dos participantes. Os dados serão codificados de forma não identificável e não conterão nenhuma informação que possa levantar suspeitas sobre a alocação dos participantes.

5.7 Intervenção

A intervenção será relatada de acordo com a lista de verificação e guia do Modelo para Descrição e Replicação da Intervenção (TIDieR) (HOFFMANN *et al*., 2014) (Anexo 3).

5.7.1 Grupo com gelo

Os participantes alocados para o 'Grupo com Gelo' receberão uma prescrição médica domiciliar para que, em posição sentada e joelho fletido em 90°, imergir o tornozelo afetado em um nível acima da dor e/ou edema, em um balde de gelo e água durante 20 minutos, até 3 vezes ao dia, durante 7 dias, além de anti-inflamatório (nimesulida 100 mg, 2 vezes ao dia, durante 5 dias), elevação do tornozelo acima da linha do peito durante o dia e orientação médica para repouso por 3 dias (KENNET *et al.*, 2007; VUURBERG *et al.*, 2018). Os participantes serão orientados individualmente diariamente por um dos pesquisadores por meio de ligações telefônicas e/ou mensagens de texto e motivados a registrar o dia e horário das aplicações de gelo por meio de um diário de intervenção para avaliação da aderência e efeitos adversos (Anexo 2).

Evidências de estudos pré-clínicos sugerem que a crioterapia pode atuar na redução da dor, inflamação e edema, levando à melhora da função do tornozelo. Na dor, a crioterapia pode diminuir a velocidade de condução nervosa, diminuindo os espasmos musculares gerados pelo reflexo espinhal após o trauma (NADLER *et al.*, 2004), além de estimular termorreceptores que poderiam inibir o processamento dos sinais de nocicepção pelo sistema nervoso central, aumentando a dor limiar (ALGAFLY; GEORGE, 2007). Ao diminuir a temperatura local, a crioterapia pode reduzir a demanda metabólica da área, evitando a formação de edema e o risco de lesão secundária por hipóxia pós-traumática e, consequentemente, evitando a morte celular (GUIRRO; ADIB; MÁXIMO, 1999). Outros mecanismos que podem contribuir para a redução do edema são a vasoconstrição local, que leva à diminuição do fluxo sanguíneo para o tecido e à diminuição da permeabilidade vascular (SHEPHERD *et al.*, 1983; SMITH *et al.*, 1994).

5.7.2 Grupo sem gelo

Os participantes alocados para o ‘Grupo Sem Gelo’ receberão as mesmas intervenções do ‘Grupo com Gelo’, mas sem crioterapia incluída. A prescrição constará de anti-inflamatório não esteroidal (nimesulida 100 mg, 2 vezes ao dia, durante 5 dias), elevação do tornozelo acima da linha do peito durante o dia e orientação médica para repouso por 3 dias. Os participantes do ‘Grupo Sem Gelo’ também serão instruídos individualmente por um pesquisador por meio de ligações telefônicas e/ou mensagens de texto.

6 ANÁLISE DOS DADOS

6.1. Cálculo amostral

O cálculo amostral foi realizado considerando o valor MCID de 9 pontos para a medição do desfecho primário (BINKLEY *et al*., 1999), e um desvio padrão de ± 12,85 baseado em um estudo anterior (BLEAKLEY; MCDONOUGH; MACAULEY, 2006). Uma amostra de 82 participantes (41 por grupo) é necessária para uma detecção mínima do tamanho do efeito, levando em consideração um poder estatístico de 80%, α de 5% e uma taxa de abandono de 20%.

6.2. Análises dos efeitos do tratamento

A análise estatística será realizada seguindo o princípio da intenção de tratar. A normalidade dos dados será testada pelo teste de Kolmogorov-Smirnov e a homocedasticidade dos dados pelo teste de Levene. Os dados paramétricos serão expressos em média e desvio padrão e analisados com Modelos de Efeitos Mistos para medidas repetidas com análise post-hoc de Bonferroni para correção. Nos casos de dados não paramétricos, serão expressos a mediana e seus limites superior e inferior e analisados usando os modelos lineares generalizados de efeitos mistos. Todas as análises estatísticas serão realizadas usando o programa SPSS Statistics (v.22.0; IBM Corp, Armonk, NY). Os tamanhos de efeito serão interpretados com base em suas diferenças clinicamente importantes mínimas (MCIDs).

6.3. Dados Perdidos

Para lidar com os dados perdidos, classificaremos como Perdas Não Aleatórias (MNAR) quando as desistências são devido à falta de eficácia e efeitos adversos, e Perdas Completamente ao Acaso (MCAR) quando a perda de acompanhamento não depende de observados ou não observados medições (por exemplo, paciente se mudando para outra cidade por motivos não relacionados à saúde). Estamos planejando realizar modelos de efeito misto para medidas repetidas (MMRM) para lidar com dados ausentes devido ao MCAR, e métodos de imputação simples (como o melhor ou o pior caso de imputação, ou seja, atribuindo o pior valor possível do resultado para desistências para uma razão negativa (falha do tratamento) e o melhor valor possível para desistências positivas (curas)) quando consideramos a falta como MNAR. Estamos planejando análises de sensibilidade para avaliar se os métodos usados para lidar com dados ausentes produzem alguma diferença importante nos resultados (European Medicines Agency, 2011).

7 RISCOS E BENEFÍCIOS

Os riscos relacionados a este estudo são mínimos, desde que se trata de uma prática já difundida no cenário clínico. OS PARTICIPANTES DESTA PESQUISA PODERÃO ESTAR EXPOSTOS AO RISCO DE CONSTRANGIMENTO E DESCONFORTO NO MOMENTO DA COLETA DOS DADOS, O QUE SERÁ MINIMIZADO AO REALIZAR AS COLETAS EM SALAS SEPARADAS PARA ESTE INTUITO, SEM A PRESENÇA DE QUAISQUER OUTROS INDIVÍDUOS QUE NÃO ESTEJAM ENVOLVIDOS DIRETAMENTE NA COLETA DE DADOS OU QUE NÃO FOREM CONVIDADOS OU ACOMPANHANTES DOS PACIENTES. Os voluntários que receberem a aplicação da crioterapia podem estar expostos a paralisia do nervo fibular, queimação por frio e/ou reações alérgicas à baixa temperatura. Em caso de ocorrência destes efeitos adversos, os voluntários serão orientados a interromper o tratamento imediatamente, e será encaminhado para uma nova consulta com os médicos DA EQUIPE VINCULADA AO P**ROJETO, TAL COMO ACONTECERIA FORA DO CONTEXTO DESTA PESQUISA**, JÁ QUE O USO DA CRIOTERAPIA (GELO) NO MANEJO DA ENTORSE AGUDA DE TORNOZELO É AMPLAMENTE UTILIZADA **NA PRÁTICA CLÍNICA DIÁRIA DA INSTITUIÇÃO**. Em caso de ser encontrado efeitos clinicamente importantes favoráveis a favor da aplicação da crioterapia, os pacientes do grupo comparador (Grupo sem gelo) serão convidados a receberem tratamento fisioterapêutico na clínica escola de fisioterapia, no departamento de fisioterapia, da UFVJM.

Os benefícios ao participar do estudo é um acompanhamento gratuito por um fisioterapeuta diariamente, provendo orientações quanto ao manejo da condição prescrito neste projeto, o que pode potenciar os efeitos do tratamento e evitar possíveis efeitos adversos. Além disso, os achados desse estudo irão ser de grande relevância para clínicos e tomadores de decisão de diferentes esferas, além da elaboração de novas diretrizes clínicas, para um manejo mais eficiente das entorses agudas de tornozelo, promovendo resolubilidade e diminuindo agravos a longo prazo, e consequentes gastos públicos.

8 CRONOGRAMA

ATIVIDADES	2022				2023						2024
	Jun-Jul	Ago-Set	Out-Nov	Dez.	Jan-Fev	Mar-Abr	Maio-Jun	Jul-Ago	Set-Out	Nov-Dez.	Jan-Fev
Submissão ao CEP	X										

Piloto de confiabilidade	X										
Recrutamento dos voluntários	X	X	X	X	X	X	X				
Alocação	X	X	X	X	X	X	X				
Aplicação da intervenção	X	X	X	X	X	X	X				
Coleta de dados	X	X	X	X	X	X	X	X	X		
Análise dos dados	X								X	X	
Síntese dos resultados										X	
Escrita dissertação											X
Publicação dos resultados											X
Atualização bibliográfica	X	X	X	X	X	X	X	X	X	X	X

9 ORÇAMENTO

Para realização da pesquisa será gasto um total de R$2326,68, sendo este valor atribuído a gastos com xerox dos questionários e escalas funcionais para avaliação dos desfechos nos 4 pontos de tempo (Linha de base, efeito imediato, curto e longo prazo), prontuários de cada voluntário e TCLE's (1 cópia para cada participante 1 cópia de cada participante para o pesquisador). Compra dos materiais de medida de desfecho (goniômetros e fitas métricas), além dos envelopes e papeis carbono utilizados para realizar as alocações. Para que o fisioterapeuta possa contatar os participantes diariamente, a assinatura de planos telefônicos mensais se vê necessária, assim como a impressão do diário de intervenção para cada voluntário. Será necessário arcar com a locomoção dos participantes para a clínica escola de fisioterapia da UFVJM, que ocorrerá para reavaliação dos desfechos a longo prazo, contabilizando a passagem da lotação de ida e volta. O valor de cada material e detalhes sobre a quantidade está detalhada na TABELA DE ORÇAMENTO. A INSTITUIÇÃO CO-PARTICIPANTE HOSPITAL NOSSA SENHORA DA SAÚDE (HNSS) NÃO IRÁ, EM NENHUMA HIPÓTESE, TER CUSTOS ADICIONAIS RELATIVOS AO FINANCIAMENTO DESTE PROJETO.

**TABELA DE ORÇAMENTO**			
**Material**	**Valor unitário**	**Quantidade**	**Valor total**

Prontuários dos voluntários	R$2,00	82	R$164,00
Xerox do TCLE	R$0,50	164	R$82,40
Cartazes para divulgação	R$2,00	5	R$10,00
Impressões de questionários e escalas	R$0,50	574	R$287,00
Envelopes	R$1,00	82	R$82,00
Papel Carbono	R$2,00	82	R$164,00
Goniômetros	R$50,00	4	R$200,00
Fitas métricas	R$3,00	4	R$12,00
Custos para ligações e SMS para voluntários	R$50,99 (mensal)	12 meses	R$611,88
Locomoção dos voluntários para reavaliação	R$10 (passagem lotação ida e volta)	82	R$800,00
Diário de intervenção	R$0,50	82	R$41,00
**Gasto total**	**-**	**-**	**R$2454.28**

REFERÊNCIAS

Algafly, A.; George, K. P. The effect of cryotherapy on nerve conduction velocity, pain threshold and pain tolerance. **Br J Sports Med**, v. 41(6), 365-369, 2007. doi:10.1136/bjsm.2006.031237

Bachmann, L. M., Kolb, E., Koller, M. T., Steurer, J., & ter Riet, G. Accuracy of Ottawa ankle rules to exclude fractures of the ankle and mid-foot: systematic review. **Bmj**, v. 326(7386), 417, 2003. doi:10.1136/bmj.326.7386.417

Beynnon, B. D., Renström, P. A., Haugh, L., Uh, B. S., & Barker, H. A prospective, randomized clinical investigation of the treatment of first-time ankle sprains. **Am J Sports Med**, v.34(9), 1401-1412, 2006. doi:10.1177/0363546506288676

Binkley, J. M., Stratford, P. W., Lott, S. A., & Riddle, D. L. The Lower Extremity Functional Scale (LEFS): scale development, measurement properties, and clinical application. **North American Orthopaedic Rehabilitation Research Network. Phys Ther**, v.79(4), 371-383, 1999.

Birrer, R. B., Fani-Salek, M. H., Totten, V. Y., Herman, L. M., & Politi, V. Managing ankle injuries in the emergency department. **J Emerg Med**, 17(4), 651-660, 1999. doi:10.1016/s0736-4679(99)00060-8

Bleakley, C. M., McDonough, S. M., & MacAuley, D. C. Cryotherapy for acute ankle sprains: A randomised controlled study of two different icing protocols. **British Journal of Sports Medicine**, v.40(8), 700-705, 2006. doi:http://dx.doi.org/10.1136/bjsm.2006.025932

Bleakley, C. M., *et al.* Effect of accelerated rehabilitation on function after ankle sprain: Randomised controlled trial. **BMJ (Online),** v.340(7756), 1122, 2010. doi:http://dx.doi.org/10.1136/bmj.c1964

Chan, A. W, *et al*. SPIRIT 2013 explanation and elaboration: guidance for protocols of clinical trials. **Bmj**, v.346, e7586, 2013. doi:10.1136/bmj.e7586

Cleland, J. A., Mintken, P. E., McDevitt, A., Bieniek, M. L., Carpenter, K. J., Kulp, K., Whitman, J. M. Manual physical therapy and exercise versus supervised home exercise in the management of patients with inversion ankle sprain: a multicenter randomized clinical trial. **Journal of Orthopaedic & Sports Physical Therapy**, v.43(7), 443-455, 2013. doi:10.2519/jospt.2013.4792

Cosby, N. L., Koroch, M., Grindstaff, T. L., Parente, W., & Hertel, J. Immediate effects of anterior to posterior talocrural joint mobilizations following acute lateral ankle sprain. **J Man Manip Ther**, v.19(2), 76-83, 2011. doi:10.1179/2042618610y.0000000005

Doherty, C., Bleakley, C., Delahunt, E., Holden, S. Treatment and prevention of acute and recurrent ankle sprain: an overview of systematic reviews with meta-analysis. **Br J Sports Med**, v.51(2), 113-125, 2017. doi:10.1136/bjsports-2016-096178

Doherty, C., Delahunt, E., Caulfield, B., Hertel, J., Ryan, J., & Bleakley, C. The incidence and prevalence of ankle sprain injury: a systematic review and meta-analysis of prospective epidemiological studies. **Sports Medicine**, v.44(1), 123-140, 2014. doi:10.1007/s40279-013-0102-5

Doig, G. S., Simpson, F. Randomization and allocation concealment: a practical guide for researchers. **Journal of critical care**, v.20(2), 187–193, 2005. https://doi.org/10.1016/j.jcrc.2005.04.005

Eldridge, S. M., Chan, C. L., Campbell, M. J., Bond, C. M., Hopewell, S., Thabane, L., Lancaster, G. A. CONSORT 2010 statement: extension to randomised pilot and feasibility trials. **Bmj**, v.355, i5239, 2016. doi:10.1136/bmj.i5239

European Medicines Agency (EMA). Guideline on missing data in confirmatory clinical trials. 1–12. [cited 2021 Nov 18], 2011. Available from: www.ema.europa.eu

Gallagher, E. J., Liebman, M., Bijur, P. E. Prospective validation of clinically important changes in pain severity measured on a visual analog scale**. Ann Emerg Med**, 38(6), 633-638, 2001. doi:10.1067/mem.2001.118863

Gogate, N., Satpute, K., Hall, T. The effectiveness of mobilization with movement on pain, balance and function following acute and sub acute inversion ankle sprain – A randomized, placebo controlled trial. **Physical Therapy in Sport**, 2020**.** doi:10.1016/j.ptsp.2020.12.016

Guirro, R., Abib, C., Máximo, C. Os efeitos fisiológicos da crioterapia: uma revisão**. Fisioterapia e Pesquisa**, **USP** v.6(2), 164-170, 1999. doi: https://doi.org/10.1590/fpusp.v6i2.79629

Gribble, P. A., Bleakley, C. M., Caulfield, B. M., Docherty, C. L., Fourchet, F., Fong, D. T., Delahunt, E. Evidence review for the 2016 International Ankle Consortium consensus statement on the prevalence, impact and long-term consequences of lateral ankle sprains. **Br J Sports Med**, v.50(24), 1496-1505, 2016. doi:10.1136/bjsports-2016-096189

Hoffmann, T. C., Glasziou, P. P., Boutron, I., Milne, R., Perera, R., Moher, D., Altman, D. G., Barbour, V., Macdonald, H., Johnston, M., Lamb, S. E., Dixon-Woods, M., McCulloch, P., Wyatt, J. C., Chan, A. W., & Michie, S. Better reporting of interventions: template for intervention description and replication (TIDieR) checklist and guide. **BMJ (Clinical research ed.**), v.348, g1687, 2014. https://doi.org/10.1136/bmj.g1687

Katz, J., & Melzack, R. Measurement of pain**. Surg Clin North Am**, v.79(2), 231-252, 1999. doi:10.1016/s0039-6109(05)70381-9

Kennet, J., Hardaker, N., Hobbs, S., Selfe, J. Cooling efficiency of 4 common cryotherapeutic agents. **Journal of Athletic Training**, v.42(3), 343, 2007.

Mawdsley, R. H., Hoy, D. K., Erwin, P. M. Criterion-related validity of the figure-of-eight method of measuring ankle edema. **Journal of Orthopaedic & Sports Physical Therapy**, v.30(3), 149-153, 2000. doi:10.2519/jospt.2000.30.3.149

Miranda, J. P., Silva, W. T., Silva, H. J., Mascarenhas, R. O., Oliveira, V. C. Effectiveness of cryotherapy on pain intensity, swelling, range of motion, function and recurrence in acute ankle sprain: A systematic review of randomized controlled trials. **Phys Ther Sport**, v.49, 243-249, 2021. doi:10.1016/j.ptsp.2021.03.011

Mutlu, S., Yılmaz, E. The effect of soft tissue injury cold application duration on symptoms, edema, joint mobility, and patient satisfaction: a randomized controlled trial. **Journal of emergency nursing**, v.46(4), 449-459, 2020. doi: 10.1016/j.jen.2020.02.017

Nabian, M. H., Zadegan, S. A., Zanjani, L. O., & Mehrpour, S. R. Epidemiology of Joint Dislocations and Ligamentous/Tendinous Injuries among 2,700 Patients: Five-year Trend of a Tertiary Center in Iran. **Arch Bone Jt Surg**, v.5(6), 426-434, 2017.

Nadler, S.F., Weingand, K., Kruse, R.J. The physiologic basis and clinical applications of cryotherapy and thermotherapy for the pain practitioner. **Pain Physician**, v.7, 395e399, 2004.

Nemet, D., Meckel, Y., Bar-Sela, S., Zaldivar, F., Cooper, D. M., & Eliakim, A. Effect of local cold-pack application on systemic anabolic and inflammatory response to sprint-interval training: a prospective comparative trial**. Eur J Appl Physiol**, v.107(4), 411-417, 2009. doi:10.1007/s00421-009-1138-y

Pereira, L. M., J. M. Dias, B. F. Mazuquin, L. G. Castanhas, M. O. Menacho, and J. R. Cardoso. Tradução, adaptação transcultural e avaliação das propriedades psicométricas do Lower Extremity Functional Scale (LEFS): LEFS-Brasil [Dissertação]. **Londrina: UniversidadeEstadual de Londrina**, 2011.

Rohner-Spengler, M., Mannion, A. F., & Babst, R. Reliability and minimal detectable change for the figure-of-eight-20 method of, measurement of ankle edema. **Journal of Orthopaedic & Sports Physical Therapy**, v. 37(4), 199-205, 2007. doi:10.2519/jospt.2007.2371

Shepherd, J. T., Rusch, N. J., & Vanhoutte, P. M. Effect of cold on the blood vessel wall. **General Pharmacology: The Vascular System**, v.14(1), 61-64, 1993. doi:10.1016/0306-3623(83)90064-2

Smith, T. L., Curl, W. W., George, C., & Rosencrance, E. Effects of contusion and cryotherapy on microvascular perfusion in rat dorsal skeletal muscle. **Pathophysiology**, v. 1(4), 229-233, 1994. doi:10.1016/0928-4680(94)90002-7

van Dijk, C. N. [CBO-guideline for diagnosis and treatment of the acute ankle injury. National organization for quality assurance in hospitals]. **Ned Tijdschr Geneeskd**, v.143(42), 2097-2101, 1999.

Vieira Ramos, G., Pinheiro, C. M., Messa, S. P., Delfino, G. B., Marqueti Rde, C., Salvini Tde, F., Durigan, J. L. Cryotherapy Reduces Inflammatory Response Without Altering Muscle Regeneration Process and Extracellular Matrix Remodeling of Rat Muscle**. Sci Rep**,v. 6, 18525, 2016. doi:10.1038/srep18525

Vuurberg, G., Hoorntje, A., Wink, L. M., van der Doelen, B. F. W., van den Bekerom, M. P., Dekker, R., Kerkhoffs, G. Diagnosis, treatment and prevention of ankle sprains: update of an evidence-based clinical guideline. **Br J Sports Med**, v.52(15), 956, 2018. doi:10.1136/bjsports-2017-098106

Youdas, J. W., McLean, T. J., Krause, D. A., Hollman, J. H. Changes in active ankle dorsiflexion range of motion after acute inversion ankle sprain. **J Sport Rehabil**, v.18(3), 358-374, 2009. doi:10.1123/jsr.18.3.358.

Apêndice 1 – TCLE

TERMO DE CONSENTIMENTO LIVRE E ESCLARECIDO (TCLE)

Prezado (a)

Você está sendo convidado(a) a participar de uma pesquisa intitulada: **"EFETIVIDADE DA CRIOTERAPIA NA INTENSIDADE DA DOR, AMPLITUDE DE MOVIMENTO DE DORSIFLEXÃO, EDEMA E FUNÇÃO NA ENTORSE DE TORNOZELO AGUDA: UM ENSAIO CONTROLADO ALEATORIZADO – O ESTUDO FROST"**, em virtude de ter procurado o serviço de emergência na cidade de Diamantina-MG após um episódio de entorse de tornozelo em até 72h, coordenada pelo Prof. Dr. Vinícius Cunha de Oliveira e contará ainda com Júlio P. de Miranda, Germano M. Coelho, Frederico S. Ataíde, Anderson J. Santos, Hytalo J. Silva.

A sua participação não é obrigatória sendo que, a qualquer momento da pesquisa, você poderá desistir e retirar seu consentimento. Sua recusa não trará nenhum prejuízo para sua relação com o pesquisador, com a UFVJM ou com Hospital Nossa Senhora da Saúde.

Os objetivos desta pesquisa é investigar a eficácia da crioterapia na intensidade da dor, edema, amplitude de movimento de dorsiflexão e função em pessoas com entorse aguda de tornozelo. Caso você decida aceitar o convite, será submetido(a) ao(s) seguinte(s) procedimentos: Primeiramente, você será SORTEADO para o grupo com gelo ou grupo sem gelo. Em seguida, passará por uma avaliação inicial, consistindo do peso, altura, sexo, idade, membro dominante, história de entorse de tornozelo anterior, capacidade de suportar peso no tornozelo afetado (Sim/Não) e se tem DOENÇAS CRÔNICAS. Dentro dessa avaliação inicial, iremos coletar algumas informações que serão investigadas neste estudo, como a amplitude de movimento o tornozelo, a intensidade da sua dor, nível de inchaço e função (capacidade) do tornozelo na sua vida diária, naquele momento. Em seguida, você receberá a prescrição do tratamento para a entorse de tornozelo, com ou sem gelo, dependendo do grupo em que você for sorteado, que durará cerca de 7 a 10 dias, e terá o acompanhamento por um fisioterapeuta por meio de celular (ligações ou mensagens, dependendo de sua preferência). Ao final dos 7 dias, você irá passar por uma reavaliação do tornozelo, que também se repetirá 3 meses depois. Assim, o tempo previsto para a sua participação é de aproximadamente 7 a 14 dias, com uma reavaliação 3 meses depois.

Os riscos relacionados com sua participação são mínimos, desde que se trata de uma prática já difundida no cenário clínico. Os voluntários que receberem a aplicação da crioterapia podem estar expostos aos seguintes efeitos adversos: paralisia do nervo fibular, CARACTERIZADO PELO SINAL DE PÉ CAÍDO, NO QUAL SE PERCEBE UMA PERCA DE FORÇA PARA LEVANTAR A PARTE DA FRENTE DOS PÉS (DORSIFLEXÃO), E DA SENSIBILIDADE DA PARTE DE CIMA DO PÉ; Queimação por frio, CARACTERIZADA POR FORMIGAMENTO E/OU DORMÊNCIA DOS PÉS, DOR EM QUEIMAÇÃO, COCEIRA, INCHAÇO, VERMELHIDÃO E BOLHAS; e/ou reações alérgicas à baixa temperatura, caracterizadas por placas vermelhas na pele, coceira, inchaço e dor nas extremidades, como dedos dos pés e das mãos. Caso apareça quaisquer efeitos adversos, os voluntários serão orientados a interromper o tratamento imediatamente, e será encaminhado para uma nova consulta com os médicos DA EQUIPE VINCULADA AO PROJETO, TAL COMO ACONTECERIA FORA DO CONTEXTO DESTA PESQUISA, JÁ QUE O USO DA CRIOTERAPIA (GELO) NO MANEJO DA ENTORSE AGUDA DE TORNOZELO É AMPLAMENTE UTILIZADA NA PRÁTICA CLÍNICA DIÁRIA DA INSTITUIÇÃO.

Os benefícios relacionados com a sua participação é um acompanhamento gratuito por um fisioterapeuta diariamente, provendo orientações quanto ao manejo da condição prescrito neste projeto, o que pode potenciar os efeitos do tratamento e evitar possíveis efeitos adversos.

Os resultados desta pesquisa poderão ser apresentados em seminários, congressos e similares, entretanto, os dados/informações pessoais obtidos por meio da sua participação serão confidenciais e sigilosos, não possibilitando sua identificação.

Não há remuneração com sua participação, bem como a de todas as partes envolvidas. Não está previsto indenização por sua participação, mas em qualquer momento se você sofrer algum dano, comprovadamente decorrente desta pesquisa, terá direito à indenização. Se aceito, o participante poderá ser ressarcido quanto aos gastos provenientes de locomoção (no preço da lotação) até o local da avaliação a longo prazo (3 semanas), na UFVJM, clínica escola de fisioterapia.

Você receberá uma via deste termo onde constam o telefone e o endereço do pesquisador principal, podendo tirar suas dúvidas sobre o projeto e sobre sua participação agora ou em qualquer momento.

Coordenador(a) do Projeto: Vinicius Cunha de Oliveira
Endereço: Rodovia MGT 367 - Km 583 - nº 5000 - Alto da Jacuba
Diamantina/MG CEP39100000.
Telefone: 38 3532-1239 (8982)

Declaro que entendi os objetivos, a forma de minha participação, riscos e benefícios da mesma e aceito o convite para participar. Autorizo a publicação dos resultados da pesquisa, a qual garante o anonimato e o sigilo referente à minha participação.

Nome do participante da pesquisa: ________________________________

Assinatura do participante da pesquisa: ____________________________

Informações – Comitê de Ética em Pesquisa da UFVJM
Rodovia MGT 367 - Km 583 - nº 5000 - Alto da Jacuba
Diamantina/MG CEP: 39.100-000
Tel.: (38) 3532-1240
Coordenador: Prof. Fábio Luiz Mendonça Martins
Secretária: Leila Adriana Gaudencio Sousa
Email: cep.secretaria@ufvjm.edu.br

Apêndice 2 – DIÁRIO DE INTERVENÇÃO

**DIÁRIO DE APLICAÇÃO DO GELO**		
Caro voluntário, favor preencher as informações abaixo referente a data de aplicação do gelo, a hora em que foi aplicado e a duração.		
**Nome completo:**		
**Data**	**Hora da aplicação**	**Duração da aplicação**
____/_____/_______		_________ minutos
____/_____/_______		_________ minutos
____/_____/_______		_________ minutos
____/_____/_______		_________ minutos
____/_____/_______		_________ minutos
____/_____/_______		_________ minutos
____/_____/_______		_________ minutos
____/_____/_______		_________ minutos
____/_____/_______		_________ minutos
____/_____/_______		_________ minutos
____/_____/_______		_________ minutos
____/_____/_______		_________ minutos
____/_____/_______		_________ minutos
____/_____/_______		_________ minutos
____/_____/_______		_________ minutos
____/_____/_______		_________ minutos
____/_____/_______		_________ minutos
____/_____/_______		_________ minutos
____/_____/_______		_________ minutos
____/_____/_______		_________ minutos
____/_____/_______		_________ minutos
____/_____/_______		_________ minutos

**Efeitos adversos da aplicação do gelo:**	
( ) Não houve efeitos adversos	( ) Queimadura<br>( ) Coceira ou alergia na pele<br>( ) Perda de movimento do pé<br><br>( ) Outro: ____

Anexo 1 – QUESTIONÁRIO LEFS (Adaptado de PEREIRA *et al.*, 2011)

LOWER EXTREMITY FUNCTIONAL SCALE (LEFS)-BRASIL

Estamos interessados em saber se você tem tido alguma dificuldade para realizar as atividades listadas abaixo, devido aos problemas com seu tornozelo. Por favor, marque uma resposta para cada atividade.

Hoje, você tem ou teria dificuldade em realizar:

Atividade	Extrema dificuldade ou incapaz	Bastante dificuldade	Moderada dificuldade	Pouca dificuldade	Sem dificuldade
a) Qualquer uma do seu trabalho normal, tarefas domésticas ou em atividades escolares	0	1	2	3	4
b) No seu passatempo ou hobbie predileto, atividades recreacionais ou esportivas	0	1	2	3	4
c) Entrar ou sair do banho	0	1	2	3	4
d) Caminhar entre os quartos e cômodos	0	1	2	3	4
e) Calçar sapatos ou meias	0	1	2	3	4
f) Agachar-se	0	1	2	3	4
g) Levantar um objeto, como uma sacola de compras no chão.	0	1	2	3	4
h) Realizar atividades domésticas leves	0	1	2	3	4
i) Realizar atividades domésticas pesadas	0	1	2	3	4
j) Entrar ou sair do carro	0	1	2	3	4
k) Andar dois quarteirões	0	1	2	3	4
l) Andar aproximadamente 1,5 km	0	1	2	3	4
m) Subir ou descer 10 degraus (aproximadamente um lance de escadas)	0	1	2	3	4
n) Ficar em pé durante uma hora.	0	1	2	3	4
o) Ficar sentado durante 1 hora.	0	1	2	3	4
p) Correr em terreno plano.	0	1	2	3	4
q) Correr em terreno irregular	0	1	2	3	4
r) Mudar de direção enquanto corre rapidamente	0	1	2	3	4
s) Pular	0	1	2	3	4
t) Rolar na cama	0	1	2	3	4

Pontuação Total: ___________________

Anexo 2 – TIDIER *CHECKLIST*

Item N°	Item	Where located **
	BRIEF NAME	
1.	Provide the name or a phrase that describes the intervention.	7
2.	Describe any rationale, theory, or goal of the elements essential to the intervention.	7
3.	Materials: Describe any physical or informational materials used in the intervention, including those provided to participants or used in intervention delivery or in training of intervention providers. Provide infO on where the materials can be accessed (e.g. online appendix, URL).	7
4.	Procedures: Describe each of the procedures, activities, and/or processes used in the intervention, including any enabling or support activities.	7
5.	For each category of intervention provider (e.g. psychologist, nursing assistant), describe expertise, background and any specific training given.	7
6.	Describe the modes of delivery (e.g. face-to-face or by some other mechanism, such as internet or telephone) of the intervention and whether it was provided individually or in a group.	7
7.	Describe the type(s) of location(s) where the intervention occurred, including any necessary infrastructure or relevant features.	7
8.	Describe the number of times the intervention was delivered and over what period of time including the number of sessions, their schedule, and their duration, intensity or dose.	7
9.	If the intervention was planned to be personalised, titrated or adapted, then describe what, why, when, and how.	N/A
10.ǂ	If the intervention was modified during the course of the study, describe the changes (what, why, when, and how).	N/A
11.	Planned: If intervention adherence or fidelity was assessed, describe how and by whom, and if any strategies were used to maintain or improve fidelity, describe.	Appendix 2
12.ǂ	Actual: If intervention adherence or fidelity was assessed, describe the extent to which the intervention was delivered as planned.	N/A

** Authors - use N/A if an item is not applicable for the intervention being described. Reviewers – use '?' if information about the element is not reported/not sufficiently reported.
† If the information is not provided in the primary paper, give details of where this information is available. This may include locations such as a published protocol or other published papers (provide citation details) or a website (provide the URL).
ǂ If completing the TIDieR checklist for a protocol, these items are not relevant to the protocol and cannot be described until the study is complete.
* We strongly recommend using this checklist in conjunction with the TIDieR guide (see *BMJ* 2014;348:g1687) which contains an explanation and elaboration for each item.
* The focus of TIDieR is on reporting details of the intervention elements (and where relevant, comparison elements) of a study. Other elements and methodological features of studies are covered by other reporting statements and checklists and have not been duplicated as part of the TIDieR checklist. When a randomised trial is being reported, the TIDieR checklist should be used in conjunction with the CONSORT statement (see **www.consort-statement.org**) as an extension of Item 5 of the CONSORT 2010 Statement. When a clinical trial protocol is being reported, the TIDieR checklist should be used in conjunction with the SPIRIT statement as an extension of Item 11 of the SPIRIT 2013 Statement (see **www.spirit-statement.org**). For alternate study designs, TIDieR can be used in conjunction with the appropriate checklist for that study design (see **www.equator-network.org**).

1. Projeto de Pesquisa:<br>EFETIVIDADE DA CRIOTERAPIA NA INTENSIDADE DA DOR, AMPLITUDE DE MOVIMENTO DE DORSIFLEXÃO, EDEMA E FUNÇÃO NA ENTORSE DE TORNOZELO AGUDA: UM ENSAIO CONTROLADO ALEATORIZADO ¿ O ESTUDO FROST
2. Número de Participantes da Pesquisa: 82
3. Área Temática:
4. Área do Conhecimento:<br>Grande Área 4. Ciências da Saúde

PESQUISADOR RESPONSÁVEL

5. Nome:<br>VINICIUS CUNHA DE OLIVEIRA			
6. CPF:<br>051.729.666-76	7. Endereço (Rua, n.º):<br>LAURA SOARES CARNEIRO 71 BURITIS 101 BELO HORIZONTE MINAS GERAIS 30575220		
8. Nacionalidade:<br>BRASILEIRO	9. Telefone:<br>31995696214	10. Outro Telefone:	11. Email:<br>viniciuscunhaoliveira@yahoo.com.br

Termo de Compromisso: Declaro que conheço e cumprirei os requisitos da Resolução CNS 466/12 e suas complementares. Comprometo-me a utilizar os materiais e dados coletados exclusivamente para os fins previstos no protocolo e a publicar os resultados sejam eles favoráveis ou não. Aceito as responsabilidades pela condução científica do projeto acima. Tenho ciência que essa folha será anexada ao projeto devidamente assinada por todos os responsáveis e fará parte integrante da documentação do mesmo.

Data: 03 / 03 / 2022

Assinatura

INSTITUIÇÃO PROPONENTE

12. Nome:<br>Universidade Federal dos Vales do Jequitinhonha e Mucuri	13. CNPJ:<br>16.888.315/0001-57	14. Unidade/Órgão:<br>Pró-Reitoria de Pesquisa e Pós-Graduação
15. Telefone:<br>(38) 3532-1200	16. Outro Telefone:	

Termo de Compromisso (do responsável pela instituição): Declaro que conheço e cumprirei os requisitos da Resolução CNS 466/12 e suas Complementares e como esta instituição tem condições para o desenvolvimento deste projeto, autorizo sua execução.

Responsável: Thiago Fonseca Silva CPF: 073.774.676-90

Cargo/Função: Pró-Reitor de Pesquisa e Pós-Graduação

Data: 08 / 03 / 2022

Assinatura

PATROCINADOR PRINCIPAL

17. Nome:	18. Telefone:	19. Outro Telefone:
6797 Universidade Federal dos Vales do Jequitinhonha e Mucuri	(38) 3532-1200	

Termo de Compromisso: Declaro que conheço e cumprirei os requisitos da Resolução CNS 466/12 e suas complementares. Comprometo-me a utilizar os materiais e dados coletados exclusivamente para os fins previstos no protocolo e a publicar os resultados sejam eles favoráveis ou não. Aceito as responsabilidades pela condução científica do projeto acima.

Nome: ______________________ CPF: ______________________

Cargo/Função: ______________________ Email: ______________________

Data: ________ / ________ / ________

____
Assinatura

Projeto de Pesquisa:
EFETIVIDADE DA CRIOTERAPIA NA INTENSIDADE DA DOR, AMPLITUDE DE MOVIMENTO DE DORSIFLEXÃO, EDEMA E FUNÇÃO NA ENTORSE DE TORNOZELO AGUDA: UM ENSAIO CONTROLADO ALEATORIZADO ¿ O ESTUDO FROST

Informações Preliminares

Responsável Principal

CPF/Documento: 051.729.666-76	Nome: VINICIUS CUNHA DE OLIVEIRA
Telefone: 31995696214	E-mail: viniciuscunhaoliveira@yahoo.com.br

Instituição Proponente

CNPJ: 16.888.315/0001-57	Nome da Instituição: Universidade Federal dos Vales do Jequitinhonha e Mucuri

Essa submissão de emenda é exclusiva do seu Centro Coordenador?

A emenda é exclusiva de seu Centro Coordenador, então as alterações realizadas em seu projeto, em virtude da emenda, NÃO serão replicadas nos Centros Participantes vinculados e nos Comitês de Ética das Instituições Coparticipantes, quando da sua aprovação.

É um estudo internacional? Não

Assistentes

CPF/Documento	Nome
093.300.396-00	Júlio Miranda

Área de Estudo

Grandes Áreas do Conhecimento

- Grande Área 4. Ciências da Saúde

Propósito Principal do Estudo

- Clínico

Título Público da Pesquisa: EFETIVIDADE DA CRIOTERAPIA NA INTENSIDADE DA DOR, AMPLITUDE DE MOVIMENTO DE DORSIFLEXÃO, EDEMA E FUNÇÃO NA ENTORSE DE TORNOZELO AGUDA: UM ENSAIO CONTROLADO ALEATORIZADO - O ESTUDO FROST

Acrônimo do Título Público: FROST

Expansão do Acrônimo do Freezing on Sprain Trial

Acrônimo FROST

Expansão do Acrônimo: Freezing on Sprain Trial

Contato Público

CPF/Documento	Nome	Telefone	E-mail
051.729.666-76	VINICIUS CUNHA DE OLIVEIRA	31995696214	viniciuscunhaoliveira@yahoo.com.br

Contato Científico: VINICIUS CUNHA DE OLIVEIRA

Desenho de Estudo / Apoio Financeiro

Desenho do Estudo: Intervenção/Experimental

Condições de saúde ou problemas

Condição de saúde ou Problema
entorse de tornozelo aguda

Descritores Gerais para as Condições de Saúde

CID1-10:Classificação Internacional de Doenças

Código CID	Descrição CID
S93	Luxacao, entorse e distensao das articulacoes e dos ligamentos ao nivel do tornozelo e do pe

DeCS:Descritores em Ciência da Saúde

Código DECS	Descrição DECS
C26.558.100	Traumatismos do Tornozelo

Descritores Específicos para as Condições de

CID1-10:Classificação Internacional de Doenças

Código CID	Descrição CID
S93	Luxacao, entorse e distensao das articulacoes e dos ligamentos ao nivel do tornozelo e do pe

DeCS:Descritores em Ciência da Saúde

Código DECS	Descrição DECS
C26.558.100	Traumatismos do Tornozelo

Tipo de Intervenção: Experimental

Natureza da Intervenção

- Outro termoterapia

Descritores da Intervenção

Descritores da Intervenção

Intervenções
Crioterapia

Lista de CID

Código CID	Descrição CID
S93	Luxacao, entorse e distensao das articulacoes e dos ligamentos ao nivel do tornozelo e do pe

Lista de DECS

Código DECS	Descrição DECS
E02.258	Crioterapia

Fase

- Fase 4

Desenho:

Ensaio controlado aleatorizado

Apoio Financeiro

CNPJ	Nome	E-mail	Telefone	Tipo
16.888.315/0001-57	Universidade Federal dos Vales do Jequitinhonha e Mucuri		3835321200	Institucional Principal

Palavra Chave

Palavra-chave
entorse de tornozelo aguda
Crioterapia

Resumo:

O objetivo deste ensaio controlado randomizado é investigar a efetividade da crioterapia na função, intensidade da dor, edema e amplitude de movimento de dorsiflexão em pessoas com um episódio agudo de entorse de tornozelo. Este é um protocolo de um ensaio controlado randomizado de dois braços. Pessoas maiores de 18 anos com diagnóstico clínico de entorse de tornozelo grau I ou II, e tempo de até 72 horas a partir do episódio da lesão, serão alocadas aleatoriamente no Grupo Gelo, que consiste em prescrição médica domiciliar para aplicação de bolsas de gelo no tornozelo com elevação, mais anti-inflamatório não esteroidal, ou Grupo Sem Gelo, que consiste na mesma prescrição médica do grupo experimental, mas sem gelo incluído. Nosso desfecho primário é função, mensurada pelo questionário Lower Extremity Functional Scale (LEFS). Nossos desfechos secundários são intensidade da dor (Escala Numérica de Dor, 0-10), edema (método da figura em oito) e amplitude de movimento de dorsiflexão (goniometria). Os acompanhamentos serão realizados no pós-tratamento (7 a 14 dias) e 12 semanas após a alocação. Um tamanho de amostra de 82 participantes será necessário para uma detecção mínima do tamanho do efeito do desfecho primário, com um poder de 80%, a de 5% e uma taxa de abandono esperada de 20%. As análises seguirão o princípio de intenção de tratar. Os efeitos do tratamento serão analisados por meio de Modelos Lineares Mistos. Os resultados deste estudo podem ajudar a esclarecer os efeitos da crioterapia no tratamento da entorse aguda de tornozelo e podem orientar clínicos na tomada de decisão.

Introdução:

A entorse de tornozelo é uma condição comum na população em geral, com prevalência de 11,88%, e incidência de sete entorses a cada 1000 exposições em atletas, sendo a lesão tornozelo-pé relacionada ao esporte que mais leva à procura de serviços de saúde (GRIBBLE et al., 2016; DOHERTY et al., 2014; NABIAN et al., 2017). Após um novo episódio de entorse de tornozelo, há uma alta frequência de instabilidade crônica do tornozelo e recorrência (GRIBBLE et al., 2016). Logo, é uma lesão musculoesquelética que pode ocasionar custos diretos (gastos com consultas médicas e medicamentos) e indiretos (afastamento do trabalho e redução da produtividade) (GRIBBLE et al., 2016). Portanto, terapias eficazes para o tratamento de entorses de tornozelo agudas são cada vez mais procuradas pelos médicos. As opções de tratamento frequentemente usadas após uma entorse de tornozelo incluem crioterapia (BLEAKLEY; MCDONOUGH; MACAULEY, 2006; VAN DIJK, 1999), tratamento cirúrgico (DOHERTY et al., 2017), mobilização articular (COSBY et al., 2011), cinesioterapia (BLEAKLEY et al., 2010; CLELAND et al., 2013), órteses (BEYNNON et al., 2006), acupuntura (DOHERTY et al., 2017), e outros. A crioterapia é uma opção de tratamento de baixo custo e fácil de usar, e tem sido recomendada por diretrizes de prática clínica para uso por profissionais de saúde em entorses de tornozelo agudas (VAN DIJK, 1999; VUURBERG et al., 2018). É considerada uma terapia potencialmente eficaz na fase inflamatória aguda devido aos achados de pesquisas pré-clínicas, que sugerem que a crioterapia pode controlar os processos inflamatórios, reduzindo a infiltração de macrófagos e os níveis de TNF-¿, NF-¿B, TGF-ß e MMP-9 mRNA (NEMET et al., 2009; VIEIRA RAMOS et al., 2016); além de promover analgesia local pela diminuição da velocidade de condução nervosa (ALGAFLY; GEORGE, 2007), o que poderia levar à melhora dos desfechos clínicos. No entanto, as evidências atuais de pesquisas clínicas que apoiam a crioterapia ainda não são claras. Uma revisão sistemática anterior (MIRANDA; SILVA; SILVA; MASCARENHAS; OLIVEIRA, 2021) investigou a eficácia da crioterapia em entorses de tornozelo agudas e constatou que a literatura carece de evidências para apoiar seu uso, levantando a importância de novos ensaios clínicos randomizados com baixa risco de viés e com grupos comparadores apropriados para isolar os efeitos das terapias (ou seja, placebo, simulação ou nenhuma intervenção) ou para investigar se a crioterapia aumenta os efeitos de outra intervenção (ou seja, crioterapia combinada com uma intervenção ativa em comparação com a mesma intervenção ativa sozinha). Considerando isso, o objetivo deste ensaio controlado randomizado é investigar a eficácia da crioterapia na função, intensidade da dor, edema e amplitude de movimento de dorsiflexão em pessoas com um episódio agudo de entorse de tornozelo.

Hipótese:

3.1. Hipótese nula: Não há diferença entre os efeitos de tratamento entre o grupo com aplicação de gelo comparado com o grupo sem a aplicação de gelo na intensidade da dor, amplitude de movimento de dorsiflexão, edema e função em pessoas com entorse aguda de tornozelo.3.2. Hipótese alternativa: Existe diferença entre os efeitos de tratamento entre o grupo com aplicação de gelo comparado com o grupo sem a aplicação de gelo na intensidade da dor, amplitude de movimento de dorsiflexão, edema e função em pessoas com entorse aguda de tornozelo.

Objetivo Primário:

Objetivo Geral
Investigar a eficácia da crioterapia na intensidade da dor, edema, amplitude de movimento de dorsiflexão (DFROM) e função em pessoas com entorse aguda de tornozelo.

Objetivo Secundário:

2.2 Objetivos Específicos
¿Investigar a eficácia da crioterapia na intensidade da dor em pessoas com entorse aguda de tornozelo POR MEIO DA ESCALA NUMÉRICA DE DOR;
¿Investigar a eficácia da crioterapia no edema em pessoas com entorse aguda de tornozelo POR MEIO DA PERIMETRIA UTILIZANDO A TECNICA DA FIGURA DE 8;
¿Investigar a eficácia da crioterapia no DFROM em pessoas com entorse aguda de tornozelo POR MEIO DA GONIOMETRIA ATIVA DO TORNOZELO;
¿Investigar a eficácia da crioterapia na função em pessoas com entorse aguda de tornozelo POR MEIO DO QUESTIONÁRIO LOWER EXTREMITY FUNCTIONAL SCALE (LEFS)
¿Investigar os efeitos adversos da aplicação da crioterapia em pessoas com entorse aguda de tornozelo.

Metodologia Proposta:

Trata-se de um ensaio controlado aleatorizado prospectivo de dois braços. Um protocolo prévio foi elaborado seguindo as recomendações da diretriz SPIRIT (CHAN et al., 2013) e será registrado no Comitê de Ética em Pesquisa da Universidade Federal dos Vales do Jequitinhonha e Mucuri (UFVJM) e, em seguida, cadastrado no site REBEC (www.ensaiosclinicos.gov.br). Será reportado de acordo com a declaração CONSORT (ELDRIDGE et al., 2016). Todos os princípios éticos fornecidos pela Declaração de Helsinque (World Medical Association, 2013) serão seguidos por todos os membros desta pesquisa ao longo do estudo.
Uma análise qualitativa baseada em entrevistas semiestruturadas para investigar as barreiras, facilitadores, e crenças dos participantes será realizada (Material Suplementar).

Critério de Inclusão:

¿ Idade entre 18 a 60 anos;
¿ Diagnóstico clínico de entorse de tornozelo grau I ou II, indicando uma ruptura incompleta de ligamento de acordo com a classificação de BIRRER et al. (1999);
¿ Tempo de até no máximo 72 horas do episódio de lesão até o dia da consulta médica;

¿ Fratura óssea excluída por radiografia ou pelas regras de Ottawa para tornozelo (BACHMANN et al., 2003).

Critério de Exclusão:

¿ Entorse de tornozelo grau III (grave), indicando ruptura complete ligamentar, determinada por um claro teste positivo de gaveta anterior e/ou teste de estresse em inversão, acompanhado por edema grave, hemorragia, alto nível de dor a palpação, e perda total da DFROM e da capacidade de sustentar peso no pé (BIRRER et al., 1999).
¿ Lesão aberta no local, que contraindique a aplicação de gelo;
¿ Ter aplicado alguma forma de crioterapia mais de uma vez desde o momento da lesão;
¿ Ter qualquer condição que contraindique a aplicação de gelo (por exemplo, síndrome de Reynaud), ou qualquer outra intervenção prescrita neste estudo.

Riscos:

Os riscos relacionados a este estudo são mínimos, desde que se trata de uma prática já difundida no cenário clínico. Os participantes desta pesquisa poderão estar expostos ao risco de constrangimento e desconforto no momento da coleta dos dados, o que será minimizado ao realizar as coletas em salas separadas para este intuito, sem a presença de quaisquer outros indivíduos que não estejam envolvidos diretamente na coleta de dados ou que não forem convidados ou acompanhantes dos pacientes. Os voluntários que receberem a aplicação da crioterapia podem estar expostos a paralisia do nervo fibular, queimação por frio e/ou reações alérgicas à baixa temperatura. Em caso de ocorrência destes efeitos adversos, os voluntários serão orientados a interromper o tratamento imediatamente, e será encaminhado para uma nova consulta com os médicos da equipe vinculada ao projeto, tal como aconteceria fora do contexto desta pesquisa, já que o uso da crioterapia (gelo) no manejo da entorse aguda de tornozelo é amplamente utilizada na prática clínica diária da instituição. Em caso de ser encontrado efeitos clinicamente importantes favoráveis a favor da aplicação da crioterapia, os pacientes do grupo comparador (grupo sem gelo) serão convidados a receberem tratamento fisioterapêutico na clínica escola de fisioterapia, no departamento de fisioterapia, da UFVJM.

Benefícios:

Os benefícios ao participar do estudo é um acompanhamento gratuito por um fisioterapeuta diariamente, provendo orientações quanto ao manejo da condição prescrito neste projeto, o que pode potenciar os efeitos do tratamento e evitar possíveis efeitos adversos. Além disso, os achados desse estudo irão ser de grande relevância para clínicos e tomadores de decisão de diferentes esferas, além da elaboração de novas diretrizes clínicas, para um manejo mais eficiente das entorses agudas de tornozelo, promovendo resolubilidade e diminuindo agravos a longo prazo, e consequentes gastos públicos.

Metodologia de Análise de Dados:

A análise estatística será realizada seguindo o princípio da intenção de tratar. A normalidade dos dados será testada pelo teste de Kolmogorov-Smirnov e a homocedasticidade dos dados pelo teste de Levene. Os dados paramétricos serão expressos em média e desvio padrão e analisados com Modelos de Efeitos Mistos para medidas repetidas com análise post-hoc de Bonferroni para correção. Nos casos de dados não paramétricos, serão expressos a mediana e seus limites superior e inferior e analisados usando os modelos lineares generalizados de efeitos mistos. Todas as análises estatísticas serão realizadas usando o programa SPSS Statistics (v.22.0; IBM Corp, Armonk, NY). Os tamanhos de efeito serão interpretados com base em suas diferenças clinicamente importantes mínimas (MCIDs).

Desfecho Primário:

Função, medida com o questionário 0-80 Lower Extremity Functional Scale (LEFS)

Desfecho Secundário:

Edema, medido pelo método em oito, que consiste na perimetria com fita métrica nas áreas de maior concentração de edema do tornozelo (região dos ligamentos talofibular anterior, calcaneofibular e tibiofibular anterior).

Intensidade da dor nas últimas 24 horas, medida com a Escala de Avaliação Numérica (NRS), que consiste em uma escala de 11 pontos, variando de 0, que corresponde a ¿Sem dor¿ a 10 ¿Pior dor imaginável¿

Amplitude de movimento de dorsiflexão ativa, medido pela goniometria ativa de tornozelo, posicionando o eixo do goniômetro aproximadamente 1,5 cm abaixo do maléolo lateral, com o braço fixo alinhado com a linha média lateral da perna e cabeça da fíbula, e o braço móvel alinhados ao quinto metatarso. O participante será instruído a realizar o máximo de dorsiflexão possível

Tamanho da Amostra no 82

Países de Recrutamento

País de Origem do Estudo	País	Nº de participantes da pesquisa
Sim	BRASIL	82

Outras Informações

Haverá uso de fontes secundárias de dados (prontuários, dados demográficos, etc)?

Não

Informe o número de indivíduos abordados pessoalmente, recrutados, ou que sofrerão algum tipo de intervenção neste centro de pesquisa:

82

Grupos em que serão divididos os participantes da pesquisa neste centro

ID Grupo	Nº de Indivíduos	Intervenções a serem realizadas
Grupo Gelo	41	Elevação do tornozelo,repouso de 3 dias, AINS e imersão em balde de gelo do tornozelo afetado
Grupo sem gelo	41	Elevação do tornozelo,repouso de 3 dias e AINS

O Estudo é Multicêntrico no Brasil?

Não

Propõe dispensa do TCLE?

Não

Haverá retenção de amostras para armazenamento em banco?

Não

Cronograma de Execução

Identificação da Etapa	Início (DD/MM/AAAA)	Término (DD/MM/AAAA)
Piloto de confiabilidade	28/03/2022	11/04/2022
Aplicação da intervenção	18/04/2022	01/01/2024
Síntese dos Resultados	01/02/2024	09/02/2024
Coleta de dados	28/04/2022	11/01/2024
Recrutamento dos voluntários	18/04/2022	01/01/2024
Atualização bibliográfica	28/03/2022	19/03/2024
Publicação dos resultados	19/02/2024	08/03/2024
Análise dos dados	22/01/2024	01/02/2024
Alocação dos voluntários	18/04/2022	01/01/2024

Orçamento Financeiro

Identificação de Orçamento	Tipo	Valor em Reais (R$)
Goniômetros	Custeio	R$ 200,00
Custos para ligações e SMS para voluntários	Custeio	R$ 611,88
Locomoção dos voluntários para reavaliação	Custeio	R$ 800,00
Xerox do TCLE	Custeio	R$ 82,40
Impressões de questionários e escalas	Custeio	R$ 287,00
Prontuários dos voluntários	Custeio	R$ 164,00
Diário de intervenção	Custeio	R$ 41,00
Fitas métricas	Custeio	R$ 12,00
Envelopes	Custeio	R$ 82,00
Cartazes para divulgação	Custeio	R$ 10,00
Papel Carbono	Custeio	R$ 164,00
Total em R$		R$ 2.454,28

Bibliografia:

Algafly, A.; George, K. P. The effect of cryotherapy on nerve conduction velocity, pain threshold and pain tolerance. Br J Sports Med, v. 41(6), 365-369, 2007. doi:10.1136/bjsm.2006.031237 Bachmann, L. M., Kolb, E., Koller, M. T., Steurer, J., & ter Riet, G. Accuracy of Ottawa ankle rules to exclude fractures of the ankle and mid-foot: systematic review. Bmj, v. 326(7386), 417, 2003. doi:10.1136/bmj.326.7386.417 Beynnon, B. D., Renström, P. A., Haugh, L., Uh, B. S., & Barker, H. A prospective, randomized clinical investigation of the treatment of first-time ankle sprains. Am J Sports Med, v.34(9), 1401-1412, 2006. doi:10.1177/0363546506288676 Binkley, J. M., Stratford, P. W., Lott, S. A., & Riddle, D. L. The Lower Extremity Functional Scale (LEFS): scale development, measurement properties, and clinical application. North American Orthopaedic Rehabilitation Research Network. Phys Ther, v.79(4), 371-383, 1999. Birrer, R. B., Fani-Salek, M. H., Totten, V. Y., Herman, L. M., & Politi, V. Managing ankle injuries in the emergency department. J Emerg Med, 17(4), 651-660, 1999. doi:10.1016/s0736-4679(99)00060-8 Bleakley, C. M., McDonough, S. M., & MacAuley, D. C. Cryotherapy for acute ankle sprains: A randomised controlled study of two different icing protocols. British Journal of Sports Medicine, v.40(8), 700-705, 2006. doi:http://dx.doi.org/10.1136/bjsm.2006.025932 Bleakley, C. M., et al. Effect of accelerated rehabilitation on function after ankle sprain: Randomised controlled trial. BMJ (Online), v.340(7756), 1122, 2010. doi:http://dx.doi.org/10.1136/bmj.c1964 Chan, A. W, et al. SPIRIT 2013 explanation and elaboration: guidance for protocols of clinical trials. Bmj, v.346, e7586, 2013. doi:10.1136/bmj.e7586 Cleland, J. A., Mintken, P. E., McDevitt, A., Bieniek, M. L., Carpenter, K. J., Kulp, K., Whitman, J. M. Manual physical therapy and exercise versus supervised home exercise in the management of patients with inversion ankle sprain: a multicenter randomized clinical trial. Journal of Orthopaedic & Sports Physical Therapy, v.43(7), 443-455, 2013. doi:10.2519/jospt.2013.4792 Cosby, N. L., Koroch, M., Grindstaff, T. L., Parente, W., & Hertel, J. Immediate effects of anterior to posterior talocrural joint mobilizations following acute lateral ankle sprain. J Man Manip Ther, v.19(2), 76-83, 2011. doi:10.1179/2042618610y.0000000005 Doherty, C., Bleakley, C., Delahunt, E., Holden, S. Treatment and prevention of acute and recurrent ankle sprain: an overview of systematic reviews with meta-analysis. Br J Sports Med, v.51(2), 113-125, 2017. doi:10.1136/bjsports-2016-096178 Doherty, C., Delahunt, E., Caulfield, B., Hertel, J., Ryan, J., & Bleakley, C. The incidence and prevalence of ankle sprain injury: a systematic review and meta-analysis of prospective epidemiological studies. Sports Medicine, v.44(1), 123-140, 2014. doi:10.1007/s40279-013-0102-5 Doig, G. S., Simpson, F. Randomization and allocation concealment: a practical guide for researchers. Journal of critical care, v.20(2), 187¿193, 2005. https://doi.org/10.1016/j.jcrc.2005.04.005 Eldridge, S. M., Chan, C. L., Campbell, M. J., Bond, C. M., Hopewell, S., Thabane, L., Lancaster, G. A. CONSORT 2010 statement: extension to randomised pilot and feasibility trials. Bmj, v.355, i5239, 2016. doi:10.1136/bmj.i5239 European Medicines Agency (EMA). Guideline on missing data in confirmatory clinical trials. 1¿12. [cited 2021 Nov 18], 2011. Available from: www.ema.europa.eu Gallagher, E. J., Liebman, M., Bijur, P. E. Prospective validation of clinically important changes in pain severity measured on a visual analog scale. Ann Emerg Med, 38(6), 633-638, 2001. doi:10.1067/mem.2001.118863 Gogate, N., Satpute, K., Hall, T. The effectiveness of mobilization with movement on pain, balance and function following acute and sub acute inversion ankle sprain ¿ A randomized, placebo controlled trial. Physical Therapy in Sport, 2020. doi:10.1016/j.ptsp.2020.12.016 Guirro, R., Abib, C., Máximo, C. Os efeitos fisiológicos da crioterapia: uma revisão. Fisioterapia e Pesquisa, USP v.6(2), 164-170, 1999. doi: https://doi.org/10.1590/fpusp.v6i2.79629 Gribble, P. A., Bleakley, C. M., Caulfield, B. M., Docherty, C. L., Fourchet, F., Fong, D. T., Delahunt, E. Evidence review for the 2016 International Ankle Consortium consensus statement on the prevalence, impact and long-term consequences of lateral ankle sprains. Br J Sports Med, v.50(24), 1496-1505, 2016. doi:10.1136/bjsports-2016-096189 Hoffmann, T. C., Glasziou, P. P., Boutron, I., Milne, R., Perera, R., Moher, D., Altman, D. G., Barbour, V., Macdonald, H., Johnston, M., Lamb, S. E., Dixon-Woods, M., McCulloch, P., Wyatt, J. C., Chan, A. W., & Michie, S. Better reporting of interventions: template for intervention description and replication (TIDieR) checklist and guide. BMJ (Clinical research ed.), v.348, g1687, 2014. https://doi.org/10.1136/bmj.g1687 Katz, J., & Melzack, R. Measurement of pain. Surg Clin North Am, v.79(2), 231-252, 1999. doi:10.1016/s0039-6109(05)70381-9 Kennet, J., Hardaker, N., Hobbs, S., Selfe, J. Cooling efficiency of 4 common cryotherapeutic agents. Journal of Athletic Training, v.42(3), 343, 2007. Mawdsley, R. H., Hoy, D. K., Erwin, P. M. Criterion-related validity of the figure-of-eight method of measuring ankle edema. Journal of Orthopaedic & Sports Physical Therapy, v.30(3), 149-153, 2000. doi:10.2519/jospt.2000.30.3.149 Miranda, J. P., Silva, W. T., Silva, H. J., Mascarenhas, R. O., Oliveira, V. C. Effectiveness of cryotherapy on pain intensity, swelling, range of motion, function and recurrence in acute ankle sprain: A systematic review of randomized controlled trials. Phys Ther Sport, v.49, 243-249, 2021. doi:10.1016/j.ptsp.2021.03.011 Mutlu, S., Y¿lmaz, E. The effect of soft tissue injury cold application duration on symptoms, edema, joint mobility, and patient satisfaction: a randomized controlled trial. Journal of emergency nursing, v.46(4), 449-459, 2020. doi: 10.1016/j.jen.2020.02.017 Nabian, M. H., Zadegan, S. A., Zanjani, L. O., & Mehrpour, S. R. Epidemiology of Joint Dislocations and Ligamentous/Tendinous Injuries among 2,700 Patients: Five-year Trend of a Tertiary Center in Iran. Arch Bone Jt Surg, v.5(6), 426-434, 2017. Nadler, S.F., Weingand, K., Kruse, R.J. The physiologic basis and clinical applications of cryotherapy and thermotherapy for the pain practitioner. Pain Physician, v.7, 395e399, 2004. Nemet, D., Meckel, Y., Bar-Sela, S., Zaldivar, F., Cooper, D. M., & Eliakim, A. Effect of local cold-pack application on systemic anabolic and inflammatory response to sprint-interval training: a prospective comparative trial. Eur J Appl Physiol, v.107(4), 411-417, 2009. doi:10.1007/s00421-009-1138-y Pereira, L. M., J. M. Dias, B. F. Mazuquin, L. G. Castanhas, M. O. Menacho, and J. R. Cardoso. Tradução, adaptação transcultural e avaliação das propriedades psicométricas do Lower Extremity Functional Scale (LEFS): LEFS-Brasil [Dissertação]. Londrina: UniversidadeEstadual de Londrina, 2011. Rohner-Spengler, M., Mannion, A. F., & Babst, R. Reliability and minimal detectable change for the figure-of-eight-20 method of, measurement of ankle edema. Journal of Orthopaedic & Sports Physical Therapy, v. 37(4), 199-205, 2007. doi:10.2519/jospt.2007.2371 Shepherd, J. T., Rusch, N. J., & Vanhoutte, P. M. Effect of cold on the blood vessel wall. General Pharmacology: The Vascular System, v.14(1), 61-64, 1993. doi:10.1016/0306-3623(83)90064-2 Smith, T. L., Curl, W. W., George, C., & Rosencrance, E. Effects of contusion and cryotherapy on microvascular perfusion in rat dorsal skeletal muscle. Pathophysiology, v. 1(4), 229-233, 1994. doi:10.1016/0928-4680(94)90002-7 van Dijk, C. N. [CBO-guideline for diagnosis and treatment of the acute ankle injury. National organization for quality assurance in hospitals]. Ned Tijdschr Geneeskd, v.143(42), 2097-2101, 1999. Vieira Ramos, G., Pinheiro, C. M., Messa, S. P., Delfino, G. B., Marqueti Rde, C., Salvini Tde, F., Durigan, J. L. Cryotherapy Reduces Inflammatory Response Without Altering Muscle Regeneration Process and Extracellular Matrix Remodeling of Rat Muscle. Sci Rep,v. 6, 18525, 2016. doi:10.1038/srep18525 Vuurberg, G., Hoorntje, A., Wink, L. M., van der Doelen, B. F. W., van den Bekerom, M. P., Dekker, R., Kerkhoffs, G. Diagnosis, treatment and prevention of ankle sprains: update of an evidence-based clinical guideline. Br J Sports Med, v.52(15), 956, 2018. doi:10.1136/bjsports-2017-098106 Youdas, J. W., McLean, T. J., Krause, D. A., Hollman, J. H. Changes in active ankle dorsiflexion range of motion after acute inversion ankle sprain. J Sport Rehabil, v.18(3), 358-374, 2009. doi:10.1123/jsr.18.3.358.

Upload de Documentos

Arquivo Anexos:

Tipo	Arquivo
Declaração de Instituição e Infraestrutura	carta_anuencia_CEP_DEPFISIOTERAPIA.pdf
TCLE / Termos de Assentimento / Justificativa de Ausência	TCLE_modificado.doc
Brochura Pesquisa	Material_Suplementar_Analise_Qualitativa.pdf
Folha de Rosto	folhaDeRostoassinada.pdf

Projeto Detalhado / Brochura Investigador	PROJETO_FROST_DETALHADO_MODIFICADO.pdf
Comprovante de Recepção	PB_COMPROVANTE_RECEPCAO_1905586.pdf
Declaração de Instituição e Infraestrutura	Carta_Coparticipe_HNSS.pdf
Outros	CARTA_RESPOSTA.pdf

Finalizar

Manter sigilo da integra do projeto de pesquisa: Sim

Prazo: Até a publicação dos resultados

Justificativa da Emenda:

Optamos pela análise qualitativa neste estudo devido à necessidade de uma compreensão profunda das percepções e experiências dos participantes em relação às barreiras, facilitadores e crenças das pessoas que sofrem entorse de tornozelo e aplicam crioterapia, devido à sua capacidade de explorar em profundidade as experiências e percepções individuais. As barreiras ao uso da crioterapia, como falta de acesso a recursos adequados, desinformação ou medo de agravar a lesão, podem ser identificadas e compreendidas através de entrevistas, permitindo aos pesquisadores captar as nuances das dificuldades enfrentadas pelos pacientes. Simultaneamente, a análise qualitativa pode revelar facilitadores importantes, como o apoio social, a orientação de profissionais de saúde e o acesso a informações confiáveis, que incentivam a adesão ao tratamento. Além disso, essa abordagem permite explorar as crenças dos pacientes sobre a eficácia da crioterapia, seus benefícios percebidos e quaisquer mitos ou preconceitos que possam influenciar seu uso. Ao captar essas perspectivas diretamente dos indivíduos afetados. Portanto, a análise qualitativa é crucial para obter uma visão holística e contextualizada dessas questões, fornecendo dados ricos e detalhados que métodos quantitativos poderiam não captar com a mesma profundidade. É importante ressaltar que a adição da análise qualitativa ao projeto original, não acarreta em mudanças de nenhum dos procedimentos metodológicos experimentais, e nem em aumento do tamanho amostral previamente estabelecido.

FEDERAL UNIVERSITY OF THE JEQUITINHONHA AND MUCURI VALLEYS
POSTGRADUATE PROGRAMME IN REHABILITATION AND FUNCTIONAL PERFORMANCE

Júlio Pascoal de Miranda

EFFECTIVENESS OF CRYOTHERAPY ON PAIN INTENSITY, DORSIFLEXION RANGE OF MOTION, OEDEMA, AND FUNCTION IN ACUTE ANKLE SPRAIN: A RANDOMISED CONTROLLED TRIAL – THE FROST STUDY

Project duration: 14/03/2022 to 14/03/2028

Supervisor: Prof. Dr. Vinícius Cunha de Oliveira

Institution: Federal University of Jequitinhonha and Mucuri Valleys

Diamantina
2022

Júlio Pascoal de Miranda

EFFECTIVENESS OF CRYOTHERAPY ON PAIN INTENSITY, DORSIFLEXION RANGE OF MOTION, OEDEMA, AND FUNCTION IN ACUTE ANKLE SPRAIN: A RANDOMISED CONTROLLED TRIAL – THE FROST STUDY

Research project presented to the stricto sensu course of the Postgraduate Programme in Rehabilitation and Functional Performance at the Federal University of the Jequitinhonha and Mucuri Valleys, as part of the requirements for completing the course.

Supervisor: Prof. Dr. Vinícius Cunha de Oliveira

Diamantina
2022

SUMMARY

1 INTRODUCTION 6

2 OBJECTIVES 7

2.1 General Objective 7

2.2 Specific Objectives 7

3 HYPOTHESIS 7

3.1. Null hypothesis 7

3.2. Alternative hypothesis 7

4 BACKGROUND 8

5 METHODS 8

5.1 Study design 8

5.2 Eligibility criteria 8

5.2.1 Inclusion criteria 8

5.2.2 Exclusion criteria 9

5.3 Procedures 9

5.4 Outcome measure 10

5.5 Randomisation 11

5.6 Blinding 11

5.7 Intervention 11

5.7.1 Group with ice 12

5.7.2 Group without ice 12

6 ANALYSING THE DATA 13

6.1. Sample calculation 13

6.2. Analyses of treatment effects 13

6.3. Lost Data 13

7 RISKS AND BENEFITS 14

8 SCHEDULE 14

9 BUDGET 15

REFERENCES..17

Appendix 1 - ICF 20

Appendix 2 - INTERVENTION DIARY...22

Annex 1 - LEFS QUESTIONNAIRE (Adapted from PEREIRA *et al.*, 2011) 23

Annex 2 - TIDIER *CHECKLIST*...24

SUMMARY

The aim of this randomised controlled trial is to investigate the effectiveness of cryotherapy on function, pain intensity, oedema and dorsiflexion range of motion in people with an acute episode of ankle sprain. This is a protocol for a two-arm randomised controlled trial. People over the age of 18 with a clinical diagnosis of grade I or II ankle sprain, and up to 72 hours from the episode of injury, will be randomly allocated to the Ice Group, which consists of a home medical prescription for the application of ice packs to the ankle with elevation, plus a non-steroidal anti-inflammatory drug, or the No Ice Group, which consists of the same medical prescription as the experimental group, but without ice included. Our primary outcome is function, measured by the Lower Extremity Functional Scale (LEFS) questionnaire. Our secondary outcomes are pain intensity (Numeric Pain Scale, 0-10), oedema (figure-of-eight method) and dorsiflexion range of motion (goniometry). Follow-ups will be carried out post-treatment (7 to 14 days) and 12 weeks after allocation. A sample size of 82 participants will be required for minimum detection of the effect size of the primary outcome, with a power of 80%, a 5% and an expected drop-out rate of 20%. The analyses will follow the intention-to-treat principle. Treatment effects will be analysed using Linear Mixed Models. The results of this study can help clarify the effects of cryotherapy in the treatment of acute ankle sprain and can guide clinicians in their decision-making.

1 INTRODUCTION

Ankle sprain is a common condition in the general population, with a prevalence of 11.88%, and an incidence of seven sprains per 1000 exposures in athletes, being the sports-related ankle-foot injury that most leads to seeking health services (GRIBBLE *et al*., 2016; DOHERTY *et al*., 2014; NABIAN *et al*., 2017). After a new episode of ankle sprain, there is a high frequency of chronic ankle instability and recurrence (GRIBBLE *et al.*, 2016). Therefore, it is a musculoskeletal injury that can cause direct costs (expenditure on medical consultations and medication) and indirect costs (time off work and reduced productivity) (GRIBBLE *et al*., 2016). Therefore, effective therapies for the treatment of acute ankle sprains are increasingly sought after by doctors.

Treatment options often used after an ankle sprain include cryotherapy (BLEAKLEY; MCDONOUGH; MACAULEY, 2006; VAN DIJK, 1999),
surgical treatment (DOHERTY *et al.*, 2017), joint mobilisation (COSBY *et al.*, 2011), kinesiotherapy (BLEAKLEY *et al*., 2010; CLELAND *et al*., 2013), orthoses (BEYNNON *et al.*, 2006), acupuncture (DOHERTY *et al*., 2017), and others. Cryotherapy is a low-cost, easy-to-use treatment option and has been recommended by clinical practice guidelines for use by healthcare professionals in acute ankle sprains (VAN DIJK, 1999; VUURBERG *et al*., 2018). It is considered a potentially effective therapy in the acute inflammatory phase due to preclinical research findings, which suggest that cryotherapy can control inflammatory processes by reducing macrophage infiltration and levels of TNF-α, NF-κB, TGF-β and MMP-9 mRNA (NEMET *et al*., 2009; VIEIRA RAMOS *et al*., 2016); in addition to
promote local analgesia by reducing nerve conduction velocity (ALGAFLY; GEORGE, 2007), which could lead to improved clinical outcomes.

However, the current evidence from clinical research supporting cryotherapy is still unclear. A previous systematic review (MIRANDA; SILVA; MASCARENHAS; OLIVEIRA, 2021) investigated the effectiveness of cryotherapy in acute ankle sprains and found that the literature lacks evidence to support its use, raising the importance of new randomised clinical trials with low risk of bias and with appropriate comparator groups to isolate the effects of therapies (i.e. placebo, sham or no intervention) or to investigate whether cryotherapy increases the effects of another intervention (i.e. cryotherapy combined with an active intervention compared to the same active intervention alone).

Considering this, the aim of this randomised controlled trial is to investigate the effectiveness of cryotherapy on function, pain intensity, oedema and dorsiflexion range of motion in people with an acute episode of ankle sprain.

2 OBJECTIVES

2.1 General Objective

To investigate the effectiveness of cryotherapy on pain intensity, oedema, dorsiflexion range motion (DFROM) and function in people with acute ankle sprains.

2.2 Specific objectives

- To investigate the effectiveness of cryotherapy on pain intensity in people with acute ankle sprains using the numerical pain scale**;**
- To investigate the effectiveness of cryotherapy on oedema in people with acute ankle sprains by means of perimetry using the figure-of-8 technique**;**
- To investigate the efficacy of cryotherapy on DFROM in people with acute ankle sprains using active ankle goniometry;
- To investigate the effectiveness of cryotherapy on function in people with acute ankle sprains using the lower extremity functional scale (LEFS) questionnaire.
- To investigate the adverse effects of cryotherapy in people with acute ankle sprains.

3 HYPOTHESIS

3.1. Null hypothesis

There was no difference between the treatment effects of the ice group and the non-ice group in terms of pain intensity, dorsiflexion range of motion, oedema and function in people with acute ankle sprains.

3.2. Alternative hypothesis

There is a difference between the effects of treatment between the group with ice application and the group without ice application on pain intensity, dorsiflexion range of motion, oedema and function in people with acute ankle sprains.

4 BACKGROUND

The use of cryotherapy is a widespread practice among clinicians and patients in the management of acute ankle sprains, and is recommended by clinical practice guidelines. However, the evidence on the effectiveness of this practice is still uncertain. A systematic review (MIRANDA *et al.*, 2021) found that there is evidence of a low level of certainty that there is no difference in effect when using or not using cryotherapy in acute ankle sprain, and that new randomised controlled trials (RCTs) with a low risk of bias and an adequate comparator group are needed to clarify the effectiveness of this practice.

5 METHODS

5.1 Study design

This is a two-arm prospective randomised controlled trial. A preliminary protocol has been drawn up following the recommendations of the SPIRIT guideline (CHAN *et al.*, 2013) and will be registered with the Research Ethics Committee of the Federal University of Jequitinhonha and Mucuri Valleys (UFVJM) and then registered on the REBEC website (www.ensaiosclinicos.gov.br). It will be reported in accordance with the CONSORT statement (ELDRIDGE *et al.*, 2016). All ethical principles provided by the Declaration of Helsinki (World Medical Association, 2013) will be followed by all members of this research throughout the study.

5.2 Sample and eligibility criteria

THE SAMPLE OF THIS STUDY WILL BE BY CONVENIENCE, made up of individuals between the ages of 18 and 60 who have suffered an episode of acute ankle sprain, RECRUITED BY SEEKING THE EMERGENCY SERVICE OF THE HOSPITAL NOSSA SENHORA DA SAÚDE, located in Diamantina-MG, Brazil, with a diagnosis later confirmed by an orthopaedic doctor at the institution.

5.2.1 Inclusion criteria

- between 18 and 60;
- Clinical diagnosis of grade I or II ankle sprain, indicating an incomplete ligament rupture according to the classification by BIRRER *et al.* (1999);
- Up to a maximum of 72 hours from the episode of injury to the day of the doctor's appointment;

- Bone fracture excluded by X-ray or Ottawa ankle rules (BACHMANN *et al.*, 2003).

5.2.2 Exclusion criteria

- Grade III (severe) ankle sprain, indicating complete ligament rupture, determined by a clear positive anterior drawer test and/or inversion stress test, accompanied by severe oedema, haemorrhage, a high level of pain on palpation, and total loss of DFROM and the ability to bear weight on the foot (BIRRER *et al.*, 1999).
- Open lesion on the site that contraindicates the application of ice;
- Having applied some form of cryotherapy more than once since the injury;
- Have any condition that contraindicates the application of ice (e.g. Reynaud's syndrome), or any other intervention prescribed in this study.

5.3 Procedures

PATIENTS WHO COME TO THE EMERGENCY DEPARTMENT COMPLAINING OF A SPRAINED ANKLE WILL BE INVITED TO TAKE PART IN THE

STUDY. All those eligible will receive information about the study and will be asked to sign an informed consent form (Appendix 1) before taking part. THE INITIAL ASSESSMENT AND REASSESSMENT OF THE PARTICIPANT WILL TAKE PLACE AT OUR LADY OF HEALTH'S HOSPITAL WITHIN THE USUAL MEDICAL CONSULTATORY, IN AN INDIVIDUALISED WAY, TO ENHANCE ANY CONSTRUCTION AT THE TIME OF COLLECTION AS WELL AS THE RISK OF DISCOMFORT FROM THE DATA COLLECTION. BOTH THE LOCATION AND THE INFORMATION OF THE INITIAL ASSESSMENT HAVE BEEN ADAPTED TO BE AS CLOSE AS POSSIBLE TO THE ORDINARY CLINICAL PRACTICE OF THE INSTITUTION'S DOCTORS, SO THAT THE PARTICIPANT WILL SPEND APPROXIMATELY THE SAME AMOUNT OF TIME AS A DOCTOR.

TIME THAN EVERYDAY LIFE. DATA COLLECTED will include age, Body Mass Index (BMI), gender, dominant limb, history of previous ankle sprain, ability to bear weight on the affected ankle (Yes/No) and whether they have comorbidities. The degree of injury will be classified according to the BIRRER *et al.* (1999) classification, which indicates Grade I or II ankle sprain, when the clinical diagnosis indicates an incomplete ligament rupture, or Grade III (severe) ankle sprain, indicating complete ligament injury, determined by a clear positive anterior drawer test and/or inversion stress test, intense oedema,

haemorrhage, a high level of pain on palpation, as well as total loss of weight-bearing capacity in the foot and DFROM. When individuals are classified as Grade III ankle sprain, they will be excluded from the study and will receive treatment based on limb immobilisation. ALL outcomes of interest (function, pain intensity, oedema and DFROM) will be collected at baseline and reassessed at the following time points: short-term (i.e. 7 to 14 days after allocation) AT NOSSA SENHORA DA SAÚDE HOSPITAL AT THE CONSULTATORY
MEDICAL WHERE THE INITIAL EVALUATION HAPPENED; and long term (i.e. 12 weeks after assignment), IN AN EVALUATION ROOM IN THE PHYSIOTHERAPY BUILDING, AT UFVJM, CAMPUS JK. In addition, we will investigate the immediate effects of cryotherapy SPECIFICALLY on pain intensity at a time point between 24h and 48h after the start of the study, BY TELEPHONE CALL. DURING THE REASSESSMENT, THE PARTICIPANT MAY SPEND BETWEEN 10 AND 30 MINUTES. IN ALL STAGES OF THIS RESEARCH, ALL BIOSAFETY STANDARDS FOR THE PREVENTION OF THE SPREAD OF COVID-19 ALREADY ESTABLISHED IN THE INSTITUTIONS PARTICIPATING IN THIS STUDY WILL BE FOLLOWED, WITH ALL RESEARCHERS MAKING USE OF THE NECESSARY PERSONAL PROTECTIVE EQUIPMENT. RE-EVALUATIONS IN SHORT-TERM FOLLOW-UPS (7 TO 14 DAYS) WILL BE EXCLUSIVE TO THE PARTICIPANTS IN THIS STUDY.

5.4 Outcome measure

Each participant will be assessed for the following outcomes: Function, measured with the 0-80 *Lower Extremity Functional Scale* (LEFS) questionnaire (Appendix 1), with a minimum clinically important difference (MCID) of 9 points (BINKLEY *et al.*, 1999); Pain intensity in the last 24 hours, measured with the Numerical Rating Scale (NRS), which consists an 11-point scale, ranging from 0, which corresponds to "No pain" to 10 "Worst pain imaginable" (KATZ; MELZACK, 1999) , with an MCID of 1.3 (95%IC = 1.0 to 1.5) (GALLAGHER; LIEBMAN; BIJUR, 2001); Oedema, measured by the figure-of-eight method, which consists of perimetry with a tape measure the areas of greatest concentration of ankle oedema (region of the anterior talofibular, calcaneofibular and anterior tibiofibular ligaments). The measurement is taken by positioning the starting point (0) of the tape measure on the midpoint between the articular projection of the anterior tibial tendon and the lateral malleolus, directing the tape towards the centre of the medial longitudinal arch of the foot, over the navicular bone, passing through the base of the fifth metatarsal and crossing the upper surface of the midfoot towards the lower point of the medial malleolus, passing through

through the calcaneal tendon, lower point of the lateral malleolus, until it meets the zero point of the tape measure (MAWDSLEY; HOY; ERWIN, 2000), and has a minimum detectable change (MDC) of 0.96 cm (ROHNER-SPENGLER; MANNION; BABST, 2007); DFROM, measured by the

active ankle goniometry, positioning the axis of the goniometer approximately 1.5 cm below the lateral malleolus, with the fixed arm aligned with the lateral midline of the leg and head of the fibula, and the movable arm aligned with the fifth metatarsal. The participant will be instructed to perform as much dorsiflexion as possible (YOUDAS *et al.*, 2009). The MDC for this measurement was determined to be 6° (YOUDAS *et al.*, 2009). Assessors will be trained in the figure-of-eight technique and DFROM goniometry, followed by a pilot study for intra- and inter-examiner reliability. Data for calculating Intraclass Correlation Coefficients (ICC) will be collected on two separate measurement occasions, 1 week apart. It is planned recruit six to ten individuals of both sexes for the pilot study, taking measurements from both lower limbs. All statistical analyses will be carried out using SPSS Statistics (v.22.0; IBM Corp, Armonk, NY).

5.5 Randomisation

The randomisation sequence for the experimental and control groups, with a 1:1 allocation ratio, will be generated by a computer program by one of the researchers who will not be involved in recruiting the participants. Randomisation will be stratified by into two Stratas ('18 to 40' and '41 to 60'). The sequence will be generated in blocks of 4, 6 and 8, in random order. The allocation will be hidden in sequentially numbered sealed opaque envelopes. The entire procedure will be conducted following the recommended methods (DOIG; SIMPSONS, 2005).

5.6 Blinding

The statistician will be blinded to the allocation of participants. The data will be coded in an unidentifiable way and will not contain any information that could raise suspicions about the allocation of participants.

5.7 Intervention

The intervention will be reported according to the checklist and guide of the Template for Intervention Description and Replication (TIDieR) (HOFFMANN *et al.*, 2014) (Appendix 3).

5.7.1 Group with ice

Participants allocated to the 'Ice Group' will receive a home medical prescription to immerse the affected ankle in a bucket of ice and water for 20 minutes, in a sitting position with the knee flexed 90° above the pain and/or oedema, up to 3 times a day, for 7 days, in addition to anti-inflammatory medication (nimesulide 100 mg, 2 times a day, for 5 days), elevation of the ankle above the chest line during the day and medical advice to rest for 3 days (KENNET *et al.*, 2007; VUURBERG *et al.*, 2018). Participants will be individually guided daily by one of the researchers via telephone calls and/or text messages and encouraged to record the day and time of the ice applications in an intervention diary to assess adherence and adverse effects (Appendix 2).

Evidence from pre-clinical studies suggests that cryotherapy can act to reduce pain, inflammation and oedema, leading to improved ankle function. In terms of pain, cryotherapy can decrease nerve conduction velocity, reducing muscle spasms generated by the spinal reflex after trauma (NADLER *et al.*, 2004), as well as stimulating thermoreceptors that could inhibit the processing of nociception signals by the central nervous system, increasing the pain threshold (ALGAFLY; GEORGE, 2007). By lowering the local temperature, cryotherapy can reduce the metabolic demand of the area, preventing the formation of oedema and the risk of secondary injury due to post-traumatic hypoxia and, consequently, preventing cell death (GUIRRO; ADIB; MÁXIMO, 1999). Other mechanisms that can contribute to reducing oedema are local vasoconstriction, which leads to a reduction in blood flow to the tissue and a reduction in vascular permeability (SHEPHERD *et al.*, 1983; SMITH *et al.*, 1994).

5.7.2 Group without ice

Participants allocated to the 'No Ice Group' will receive the same interventions as the 'Ice Group', but without cryotherapy included. The prescription will consist of a non-steroidal anti-inflammatory drug (nimesulide 100 mg, twice a day, for 5 days), elevation of the ankle above the chest line during the day and medical advice to rest for 3 days. Participants in the 'No Ice Group' will also be instructed individually by a researcher via telephone calls and/or text messages.

6 ANALYSING DATA

6.1. Sample calculation

The sample calculation was carried out considering the MCID value of 9 points for measuring the primary outcome (BINKLEY *et al.*, 1999), and a standard deviation of± 12.85 based on a previous study (BLEAKLEY; MCDONOUGH; MACAULEY, 2006). A sample 82 participants (41 per group) is required for minimum effect size detection, taking into account a statistical power of 80 per cent, α of 5 per cent and a drop-out rate of 20 per cent.

6.2. Analyses of treatment effects

The statistical analysis will be carried out using the intention-to-treat principle. Data normality will be tested using the Kolmogorov-Smirnov test and data homoscedasticity using the Levene test. Parametric data will be expressed as mean and standard deviation and analysed using Mixed Effects Models for repeated measures with Bonferroni post hoc analysis for correction. In the case of non-parametric data, the median and its upper and lower limits will be expressed and analysed using generalised linear mixed-effects models. All statistical analyses will be carried out using SPSS Statistics (v.22.0; IBM Corp, Armonk, NY). Effect sizes will be interpreted based on their minimal clinically important differences (MCIDs).

6.3. Lost Data

To deal with missing data, we will classify them as Non-Randomised Losses (NRL) when the dropouts are due to lack of efficacy and adverse effects, and Completely Chance Losses (CCL) when the loss to follow-up does not depend on observed or unobserved measurements (e.g. patient moving to another city for non-health reasons). We are planning to carry out mixed effect models for repeated measures (MMRM) to deal with missing data due to MCAR, and simple imputation methods (such as best or worst case imputation, i.e. assigning the worst possible value of the outcome for dropouts for a negative reason (treatment failure) and the best possible value for positive dropouts (cures)) when we consider missingness as MNAR. We are planning sensitivity analyses to assess whether the methods used to deal with missing data produce any important difference in the results (European Medicines Agency, 2011).

7 RISKS AND BENEFITS

The risks related to this study are minimal, since it is a practice that is already widespread in clinical settings. THE PARTICIPANTS IN THIS RESEARCH MAY BE EXPOSED TO THE RISK OF EMBARRASSMENT AND DISCOMFORT AT THE TIME OF DATA COLLECTION, WHICH WILL BE MINIMISED BY CARRYING OUT THE COLLECTIONS IN SEPARATE ROOMS FOR THIS PURPOSE, WITHOUT THE PRESENCE OF ANY OTHER INDIVIDUALS WHO ARE NOT DIRECTLY INVOLVED IN THE DATA COLLECTION OR WHO ARE NOT INVITED OR INVITED TO PARTICIPATE.
ACCOMPANYING PATIENTS. Volunteers receiving cryotherapy may be exposed to paralysis of the peroneal nerve, frostbite and/or allergic reactions to the low temperature. If these adverse effects occur, the volunteers will be advised to stop treatment immediately and will be referred for a new consultation with the doctors on the team linked to the **project, AS WOULD HAPPEN OUTSIDE THE CONTEXT OF THIS RESEARCH**, SINCE THE USE OF CRYOTHERAPY (ICE) IN THE MANAGEMENT OF ACUTE THROAT ENTORSE IS WIDELY USED **IN THE INSTITUTION'S DAILY CLINICAL PRACTICE**. In
If clinically important effects in favour of the application of cryotherapy are found, the patients in the comparator group (Group without ice) will be invited to receive physiotherapy treatment at the physiotherapy school clinic in the physiotherapy department at UFVJM.

The benefits of taking part in the study are free daily follow-up by a physiotherapist, providing guidance on the management of the condition prescribed in this project, which can enhance the effects of the treatment and avoid possible adverse effects. In addition, the findings of this study will be of great relevance to clinicians and decision-makers in different spheres, as well as the development new clinical guidelines for more efficient management of acute ankle sprains, promoting resolubility and reducing long-term problems and consequent public spending.

8 SCHEDULE

ACTIVITIES	2022				2023						2024
	Jun-Jul	Aug-Sept	Oct-Nov	Dec.	Jan-Feb	Mar-Apr	May-June	Jul-Aug	Sept-Oct	Nov-Dec.	Jan-Feb
Submission to the CEP	X										

Reliability pilot	X										
Recruiting volunteers	X	X	X	X	X	X	X				
Allocation	X	X	X	X	X	X	X				
Implementation of the intervention	X	X	X	X	X	X	X				
Data collection	X	X	X	X	X	X	X	X	X		
Analysing the data	X								X	X	
Summary of results										X	
Dissertation writing											X
Publication of results											X
Bibliographic update	X	X	X	X	X	X	X	X	X	X	X

9 BUDGET

To carry out the research, a total of R$2326.68 will be spent, this amount being attributed to the cost of photocopying the questionnaires and functional scales to assess the outcomes at the 4 time points (baseline, immediate effect, short and long term), medical records for each volunteer and ICFs (1 copy for each participant 1 copy for each participant for the researcher). Purchase of the outcome measurement materials (goniometers and tapes), as well as the envelopes and carbon paper used to make the allocations. In order for the physiotherapist to be able to contact the participants on a daily basis, it will be necessary to subscribe to monthly telephone plans, as well as to print out the intervention diary for each volunteer. It will be necessary to pay for the participants' travel to the physiotherapy school clinic at UFVJM, which will take place for the reassessment of long-term outcomes, accounting for the . The value of each material and details of the quantity are detailed in the BUDGET TABLE. THE CO-PARTICIPATING INSTITUTION HOSPITAL NOSSA SENHORA DA SAÚDE (HNSS) WILL NOT, UNDER ANY CIRCUMSTANCES, INCUR ADDITIONAL COSTS RELATED TO THE FINANCING OF THIS PROJECT.

**BUDGET TABLE**			
**Material**	**Unit value**	**Quantity**	**Total value**

Volunteers' records	R$2,00	82	R$164,00
Xerox of the ICF	R$0,50	164	R$82,40
Posters for publicity	R$2,00	5	R$10,00
Printouts of questionnaires and scales	R$0,50	574	R$287,00
Envelopes	R$1,00	82	R$82,00
Carbon paper	R$2,00	82	R$164,00
Goniometers	R$50,00	4	R$200,00
Measuring tapes	R$3,00	4	R$12,00
Costs for calls and SMS for volunteers	R$50.99 (monthly)	12 months	R$611,88
Transport of volunteers for re-evaluation	R$10 (return ticket)	82	R$800,00
Intervention diary	R$0,50	82	R$41,00
**Total expenditure**	**-**	**-**	**R$2454.28**

REFERENCES

Algafly, A.; George, K. P. The effect of cryotherapy on nerve conduction velocity, pain threshold and pain tolerance. **Br J Sports Med**, v. 41(6), 365-369, 2007. doi:10.1136/bjsm.2006.031237

Bachmann, L. M., Kolb, E., Koller, M. T., Steurer, J., & ter Riet, G. Accuracy of Ottawa ankle rules to exclude fractures of the ankle and mid-foot: systematic review. **Bmj**, v. 326(7386), 417, 2003. doi:10.1136/bmj.326.7386.417

Beynnon, B. D., Renström, P. A., Haugh, L., Uh, B. S., & Barker, H. A prospective, randomised clinical investigation of the treatment of first-time ankle sprains. **Am J Sports Med**, v.34(9), 1401-1412, 2006. doi:10.1177/0363546506288676

Binkley, J. M., Stratford, P. W., Lott, S. A., & Riddle, D. L. The Lower Extremity Functional Scale (LEFS): scale development, measurement properties, and clinical application. **North American Orthopaedic Rehabilitation Research Network. Phys Ther**, v.79(4), 371-383, 1999.

Birrer, R. B., Fani-Salek, M. H., Totten, V. Y., Herman, L. M., & Politi, V. Managing ankle injuries in the emergency department. **J Emerg Med**, 17(4), 651-660, 1999. doi:10.1016/s0736-4679(99)00060-8

Bleakley, C. M., McDonough, S. M., & MacAuley, D. C. Cryotherapy for acute ankle sprains: A randomised controlled study of two different icing protocols. **British Journal of Sports Medicine**, v.40(8), 700-705, 2006. doi:http://dx.doi.org/10.1136/bjsm.2006.025932

Bleakley, C. M., *et al.* Effect of accelerated rehabilitation on function after ankle sprain: Randomised controlled trial. **BMJ (Online),** v.340(7756), 1122, 2010. doi:http://dx.doi.org/10.1136/bmj.c1964

Chan, A. W, *et al*. SPIRIT 2013 explanation and elaboration: guidance for protocols of clinical trials. **Bmj**, v.346, e7586, 2013. doi:10.1136/bmj.e7586

Cleland, J. A., Mintken, P. E., McDevitt, A., Bieniek, M. L., Carpenter, K. J., Kulp, K., Whitman, J. M. Manual physical therapy and exercise versus supervised home exercise in the management of patients with inversion ankle sprain: a multicentre randomized clinical trial. **Journal of Orthopaedic & Sports Physical Therapy**, v.43(7), 443-455, 2013. doi:10.2519/jospt.2013.4792

Cosby, N. L., Koroch, M., Grindstaff, T. L., Parente, W., & Hertel, J. Immediate effects of anterior to posterior talocrural joint mobilisations following acute lateral ankle sprain. **J Manip Ther**, v.19(2), 76-83, 2011. doi:10.1179/2042618610y.0000000005

Doherty, C., Bleakley, C., Delahunt, E., Holden, S. Treatment and prevention of acute and recurrent ankle sprain: an overview systematic reviews with meta-analysis. **Br J Sports Med**, v.51(2), 113-125, 2017. doi:10.1136/bjsports-2016-096178

Doherty, C., Delahunt, E., Caulfield, B., Hertel, J., Ryan, J., & Bleakley, C. The incidence and prevalence of ankle sprain injury: a systematic review and meta-analysis of prospective epidemiological studies. **Sports Medicine**, v.44(1), 123-140, 2014. doi:10.1007/s40279-013-0102-5

Doig, G. S., Simpson, F. Randomisation and allocation concealment: a practical guide for researchers. **Journal of critical care**, v.20(2), 187-193, 2005. https://doi.org/10.1016/j.jcrc.2005.04.005

Eldridge, S. M., Chan, C. L., Campbell, M. J., Bond, C. M., Hopewell, S., Thabane, L., Lancaster, G. A. CONSORT 2010 statement: extension to randomised pilot and feasibility trials. **Bmj**, v.355, i5239, 2016. doi:10.1136/bmj.i5239

European Medicines Agency (EMA). Guideline on missing data in confirmatory clinical trials. 1-12. [cited 2021 Nov 18], 2011. Available from: www.ema.europa.eu

Gallagher, E. J., Liebman, M., Bijur, P. E. Prospective validation of clinically important changes in pain severity measured on a visual analogue scale**. Ann Emerg Med**, 38(6), 633-638, 2001. doi:10.1067/mem.2001.118863

Gogate, N., Satpute, K., Hall, T. The effectiveness of mobilisation with movement on pain, balance and function following acute and sub acute inversion ankle sprain - A randomized, placebo controlled trial. **Physical Therapy in Sport**, 2020**.** doi:10.1016/j.ptsp.2020.12.016

Guirro, R., Abib, C., Máximo, C. The physiological effects of cryotherapy: a review**. Fisioterapia e Pesquisa**, **USP** v.6(2), 164-170, 1999. doi: https://doi.org/10.1590/fpusp.v6i2.79629

Gribble, P. A., Bleakley, C. M., Caulfield, B. M., Docherty, C. L., Fourchet, F., Fong, D. T., Delahunt, E. Evidence review for the 2016 International Ankle Consortium consensus statement on the prevalence, impact and long-term consequences of lateral ankle sprains. **Br J Sports Med**, v.50(24), 1496-1505, 2016. doi:10.1136/bjsports-2016-096189

Hoffmann, T. C., Glasziou, P. P., Boutron, I., Milne, R., Perera, R., Moher, D., Altman, D. G., Barbour, V., Macdonald, H., Johnston, M., Lamb, S. E., Dixon-Woods, M., McCulloch, P., Wyatt, J. C., Chan, A. W., & Michie, S. Better reporting of interventions: template for intervention description and replication (TIDieR) checklist and guide. **BMJ (Clinical research ed.**), v.348, g1687, 2014. https://doi.org/10.1136/bmj.g1687

Katz, J., & Melzack, R. Measurement of pain**. Surg Clin North Am**, v.79(2), 231-252, 1999. doi:10.1016/s0039-6109(05)70381-9

Kennet, J., Hardaker, N., Hobbs, S., Selfe, J. Cooling efficiency of 4 common cryotherapeutic agents. **Journal Athletic Training**, v.42(3), 343, 2007.

Mawdsley, R. H., Hoy, D. K., Erwin, P. M. Criterion-related validity of the figure-of-eight method of measuring ankle oedema. **Journal of Orthopaedic & Sports Physical Therapy**, v.30(3), 149-153, 2000. doi:10.2519/jospt.2000.30.3.149

Miranda, J. P., Silva, W. T., Silva, H. J., Mascarenhas, R. O., Oliveira, V. C. Effectiveness of cryotherapy on pain intensity, swelling, range of motion, function and recurrence in acute ankle sprain: A systematic review randomized controlled trials. **Phys Ther Sport**, v.49, 243-249, 2021. doi:10.1016/j.ptsp.2021.03.011

Mutlu, S., Yılmaz, E. The effect of soft tissue injury cold application duration on symptoms, oedema, joint mobility, and patient satisfaction: a randomized controlled trial. **Journal of emergency nursing**, v.46(4), 449- 459, 2020. doi: 10.1016/j.jen.2020.02.017

Nabian, M. H., Zadegan, S. A., Zanjani, L. O., & Mehrpour, S. R. Epidemiology of Joint Dislocations and Ligamentous/Tendinous Injuries among 2,700 Patients: Five-year Trend of a Tertiary Centre in Iran. **Arch Bone Jt Surg**, v.5(6), 426-434, 2017.

Nadler, S.F., Weingand, K., Kruse, R.J. The physiologic basis and clinical applications of cryotherapy and thermotherapy for the pain practitioner. **Pain Physician**, v.7, 395e399, 2004.

Nemet, D., Meckel, Y., Bar-Sela, S., Zaldivar, F., Cooper, D. M., & Eliakim, A. Effect of local cold-pack application on systemic anabolic and inflammatory response to sprint-interval training: a prospective comparative trial**. Eur J Appl Physiol**, v.107(4), 411-417, 2009. doi:10.1007/s00421-009-1138-y

Pereira, L. M., J. M. Dias, B. F. Mazuquin, L. G. Castanhas, M. O. Menacho, and J. R. Cardoso. Translation, cross-cultural adaptation and evaluation of the psychometric properties of the Lower Extremity Functional Scale (LEFS): LEFS-Brazil [Dissertation]. **Londrina: State University of Londrina**, 2011.

Rohner-Spengler, M., Mannion, A. F., & Babst, R. Reliability and minimal detectable change for the figure-of-eight-20 method of, measurement of ankle oedema. **Journal of Orthopaedic & Sports Physical Therapy**, v. 37(4), 199-205, 2007. doi:10.2519/jospt.2007.2371

Shepherd, J. T., Rusch, N. J., & Vanhoutte, P. M. Effect of cold on the blood vessel wall. **General Pharmacology: The Vascular System**, v.14(1), 61-64, 1993. doi:10.1016/0306-3623(83)90064-2

Smith, T. L., Curl, W. W., George, C., & Rosencrance, E. Effects of contusion and cryotherapy on microvascular perfusion in rat dorsal skeletal muscle. **Pathophysiology**, v. 1(4), 229-233, 1994. doi:10.1016/0928- 4680(94)90002-7

van Dijk, C. N. [CBO-guideline for diagnosis and treatment of the acute ankle injury. National organisation for quality assurance in hospitals]. **Ned Tijdschr Geneeskd**, v.143(42), 2097-2101, 1999.

Vieira Ramos, G., Pinheiro, C. M., Messa, S. P., Delfino, G. B., Marqueti Rde, C., Salvini Tde, F., Durigan, J. L. Cryotherapy Reduces Inflammatory Response Without Altering Muscle Regeneration Process and Extracellular Matrix Remodelling of Rat Muscle**. Sci Rep**,v. 6, 18525, 2016. doi:10.1038/srep18525

Vuurberg, G., Hoorntje, A., Wink, L. M., van der Doelen, B. F. W., van den Bekerom, M. P., Dekker, R., Kerkhoffs, G. Diagnosis, treatment and prevention of ankle sprains: update of evidence-based clinical guideline. **Br J Sports Med**, v.52(15), 956, 2018. doi:10.1136/bjsports-2017-098106

Youdas, J. W., McLean, T. J., Krause, D. A., Hollman, J. H. Changes in active ankle dorsiflexion range of motion after acute inversion ankle sprain. **J Sport Rehabil**, v.18(3), 358-374, 2009. doi:10.1123/jsr.18.3.358.

Appendix 1 - TCLE

TERM OF FREE AND INFORMED CONSENT (TCLE)

Dear Sir

You are being invited to take part in a research project entitled: **"EFFECTIVENESS CRIOTHERAPY ON PAIN INTENSITY, DORSIFLEXION MOVEMENT AMPLITUDE, EDEMA AND FUNCTION IN THORNKIN ENTORSE ACUTE: ONE CONTROLLED ALEATORISED - O FROST STUDY"**, as a result of having sought the emergency service in the city of Diamantina-MG after an episode of ankle sprain within 72 hours, coordinated by Prof Dr Vinícius Cunha de Oliveira and will also include Júlio P. de Miranda, Germano M. Coelho, Frederico S. Ataíde, Anderson J. Santos, Hytalo J. Silva.

Your participation is not compulsory and you may withdraw your consent at any time during the research. Your refusal will not jeopardise your relationship with the researcher, UFVJM or Hospital Nossa Senhora da Saúde.

The aim of this research is to investigate the effectiveness of cryotherapy on pain intensity, oedema, dorsiflexion range of motion and function in people with acute ankle sprains. If you decide to accept the invitation, you will be subjected to the following procedure(s): Firstly, you will be SELECTED for the group with ice or the group without ice. You will then undergo an initial assessment, consisting of your weight, height, gender, age, dominant limb, history of previous ankle sprain, ability to bear weight on the affected ankle (Yes/No) and whether you have CHRONIC DISEASES. Within this initial assessment, we will collect some information that will be investigated in this study, such as the range of movement of the ankle, the intensity of your pain, level of swelling and function (capacity) of the ankle in your daily life at that time. You will then receive a prescription for ankle sprain treatment, with or without ice, depending on the group you are drawn into, which will last around 7 to 10 days, and you will be monitored by a physiotherapist via mobile phone (calls or messages, depending on your preference). At the end of the 7 days, you will undergo a reassessment of your ankle, which will also be repeated 3 months later. This means that you can expect to take part in the programme for between 7 and 14 days, with a reassessment 3 months later.

The risks related to their participation are minimal, since it is a practice that is already widespread in the clinical setting. Volunteers who receive cryotherapy may be exposed to the following adverse effects: paralysis of the peroneal nerve, CHARACTERISED BY THE SIGN OF A FALLEN FOOT, IN WHICH A LOSS OF FORCE TO LIFT THE FRONT OF THE FEET (DORSIFLEXION), AND OF THE SENSITIVITY OF THE TOP OF THE FOOT IS PERCEIVED; Frostbite, CHARACTERISED BY TINGLING AND/OR SLEEPING OF THE FEET, BURNING PAIN, ITCHING, SWELLING, REDNESS AND BUBBLES; and/or reactions allergic to low temperatures, characterised by red plaques on the skin, itching, swelling and pain in the extremities, such as the and toes. If any adverse effects appear, the volunteers will be instructed to stop the treatment immediately, and will be referred for a new consultation with the doctors of the TEAM LINKED TO THE PROJECT, AS WOULD HAPPEN OUTSIDE THE CONTEXT OF THIS , AS THE USE OF CRYOTHERAPY (ICE) IN THE MANAGEMENT OF ACUTE TOEBONE BURNING IS WIDELY USED IN THE INSTITUTION'S DAILY CLINICAL PRACTICE.

The benefits related to your participation are that you will receive free monitoring by a physiotherapist on a daily basis, providing guidance on the management of the condition prescribed in this project, which can enhance the effects of the treatment and avoid possible adverse effects. The results of this research may be presented at seminars, congresses and the like, however, the personal data/information obtained through your participation will be kept confidential.
confidential and secretive, and cannot be identified.

There is no remuneration for your participation, or that of all parties involved. There is no provision for compensation for your participation, but at any time if you suffer any proven damage as a result of this research, you will be entitled to compensation. If accepted, the participant may be reimbursed for travel expenses (at the price of the allotment) to the site of the long-term assessment (3 weeks), at UFVJM, the physiotherapy school clinic.

You will receive a copy of this form with the telephone number and address of the principal investigator, and you can ask any questions you may have about the project and your participation now or at any time.

Project Coordinator: Vinicius Cunha de Oliveira
Address: Rodovia MGT 367 - Km 583 - nº 5000 - Alto da Jacuba
Diamantina/MG CEP39100000.
Telephone: 38 3532-1239 (8982)

I declare that I have understood the objectives, the form of my participation, the risks and benefits and I accept the invitation to take part. I authorise the publication of the research results, which guarantees the anonymity and confidentiality of my participation.

Name of research participant: ______________________________________

Signature of research participant: __________________________________

Information - UFVJM Research Ethics Committee Rodovia MGT 367 - Km 583 - nº 5000 - Alto da Jacuba Diamantina/MG CEP: 39.100-000
Tel: (38) 3532-1240
Coordinator: Prof Fábio Luiz Mendonça Martins
Secretary: Leila Adriana Gaudencio Sousa Email: cep.secretaria@ufvjm.edu.br

Appendix 2 - DIARY OF INTERVENTION

ICE APPLICATION DIARY		
Dear volunteer, please fill in the information below regarding the date the ice was applied, the time it was applied and the duration.		
**Full name:**		
**Date**	**Time of application**	**Duration of application**
____/_____/_______		__________minutes
____/_____/_______		__________minutes
____/_____/_______		__________minutes
____/_____/_______		__________minutes
____/_____/_______		__________minutes
____/_____/_______		__________minutes
____/_____/_______		__________minutes
____/_____/_______		__________minutes
____/_____/_______		__________minutes
____/_____/_______		__________minutes
____/_____/_______		__________minutes
____/_____/_______		__________minutes
____/_____/_______		__________minutes
____/_____/_______		__________minutes
____/_____/_______		__________minutes
____/_____/_______		__________minutes
____/_____/_______		__________minutes
____/_____/_______		__________minutes
____/_____/_______		__________minutes
____/_____/_______		__________minutes
____/_____/_______		__________minutes
____/_____/_______		__________minutes
**Adverse effects of ice application:**		
( ) There were no adverse effects	( ) Burn<br>( ) Itchy or allergic skin ( )<br>Loss of movement in the foot<br><br>( ) Other: ____	

Annex 1 - LEFS QUESTIONNAIRE (Adapted from PEREIRA *et al.*, 2011)

**LOWER EXTREMITY FUNCTIONAL SCALE (LEFS)-BRASIL**					
We are interested to know if you have had any difficulty carrying out the activities listed below due to problems with your ankle. Please tick one for each activity. **Today, you have or would have difficulty realising it:**					
Activity	Extreme difficulty or incapable	Quite difficult	Moderate difficulty	Little difficulty	Without difficulty
a) Any of your normal work, household chores or school activities	0	1	2	3	4
b) Your favourite hobby or pastime, recreational or sporting activities	0	1	2	3	4
c) Getting in or out of the bath	0	1	2	3	4
d)Walking between rooms and rooms	0	1	2	3	4
e) Wearing shoes or socks	0	1	2	3	4
f) Squatting	0	1	2	3	4
g) Lifting an object, such as a carrier bag. shopping on the floor.	0	1	2	3	4
h) Carry out light domestic activities	0	1	2	3	4
i) Carry out heavy domestic activities	0	1	2	3	4
j) Getting in or out of the car	0	1	2	3	4
k) Walk two blocks	0	1	2	3	4
l) Walk approximately 1.5 kilometres	0	1	2	3	4
m) Up or down 10 steps (approximately one flight of stairs)	0	1	2	3	4
n) Stand for one hour.	0	1	2	3	4
o)Sit for 1 hour.	0	1	2	3	4
p)Running on flat ground.	0	1	2	3	4
q)Running on uneven ground	0	1	2	3	4
r) Changing direction while running quickly	0	1	2	3	4
s) Jump	0	1	2	3	4
t) Rolling over in bed	0	1	2	3	4
**Total Score:** ________________________					

Annex 2 - TIDIER *CHECKLIST*

Item No.	Item	Where located **
	BRIEF NAME	
1.	Provide the name or a phrase that describes the intervention.	7
2.	Describe any rationale, theory, or goal of the elements essential to the intervention.	7
3.	Materials: Describe any physical or informational materials used in the intervention, including those provided to participants or used in intervention delivery or in training of intervention providers. Provide infO on where the materials can be accessed (e.g. online appendix, URL).	7
4.	Procedures: Describe each of the procedures, activities, and/or processes used in the intervention, including any enabling or support activities.	7
5.	For each category of intervention provider (e.g. psychologist, nursing assistant), describe expertise, background and any specific training given.	7
6.	Describe the modes of delivery (e.g. face-to-face or by some other mechanism, such internet or telephone) of the intervention and whether it was provided individually or in a group.	7
7.	Describe the type(s) of location(s) where the intervention occurred, including any necessary infrastructure or relevant features.	7
8.	Describe the number of times the intervention was delivered and over what period of time including the number of sessions, their schedule, and their duration, intensity or dose.	7
9.	If the intervention was planned to be personalised, titrated or adapted, then describe what, why, when, and how.	N/A
10.ǂ	If the intervention was modified during the course of the study, describe the changes (what, why, when, and how).	N/A
11.	Planned: If intervention adherence or fidelity was assessed, describe how and by whom, and if any strategies were used to maintain or improve fidelity, describe.	Appendix 2
12.ǂ	Actual: If intervention adherence or fidelity was assessed, describe the extent to which the intervention was delivered as planned.	N/A

** Authors - use N/A if an item is not applicable for the intervention being described. Reviewers - use '?' if information about the element is not reported/not sufficiently reported.
† If the information is not provided in the primary paper, give details of where this information is available. This may include locations such as a published protocol or other published papers (provide citation details) or a website (provide the URL).
ǂ If completing the TIDieR checklist for a protocol, these items are not relevant to the protocol and cannot be described until the study is complete.
* We strongly recommend using this checklist in conjunction with the TIDieR guide (see *BMJ* 2014;348:g1687) which contains an explanation and elaboration for each item.
* The focus of TIDieR is on reporting details of the intervention elements (and where relevant, comparison elements) of a study. Other elements and methodological features of studies are covered by other reporting statements and checklists and have not been duplicated as part of the TIDieR checklist. When a randomised trial is being reported, the TIDieR checklist should be used in conjunction with the CONSORT statement (see **www.consort-statement.org**) as an extension of Item 5 of the CONSORT 2010 Statement. When a clinical trial protocol is being reported, the TIDieR checklist should be used in conjunction with the SPIRIT statement as an extension of Item 11 of the SPIRIT 2013 Statement (see **www.spirit-statement.org**). For alternate study designs, TIDieR can be used in conjunction with the appropriate checklist for that study design (see www.equator-network.org).

1. Research Project:
EFFECTIVENESS OF CRYOTHERAPY ON PAIN INTENSITY, DORSIFLEXION RANGE OF MOTION, EDEMA AND FUNCTION IN ACUTE ANKLE SPRAIN: A RANDOMIZED CONTROLLED TRIAL - THE FROST STUDY
2. Number of Research Participants: 82
3. Thematic Area:
4. Knowledge Area: Major Area 4. Health Sciences

RESPONSIBLE RESEARCHER

5. Name: VINICIUS CUNHA OF OLIVEIRA			
6. CPF: 051.729.666-76	7. Address (Street, No.): LAURA SOARES CARNEIRO 71 BURITIS 101 BELO HORIZONTE MINAS GERAIS 30575220		
8. Nationality: BRAZILIAN	9. Telephone: 31995696214	10. Other Phone:	11. Email: viniciuscunhaoliveira@yahoo.com.br

Term of Commitment: I declare that I am aware of and will comply with the requirements of CNS Resolution 466/12 and its complementary provisions. I undertake to use the materials and data collected exclusively for the purposes set forth in the protocol and to publish the results, whether favorable or not.

I accept responsibility for the scientific management of the above project. I am aware that this sheet will be attached to the project, duly signed by all those responsible, and will be an integral part of the project documentation.

Date: 03 / 03 / 2022

Signature

PROPOSING INSTITUTION

12. Name: Federal University of Jequitinhonha and Mucuri Valleys	13. CNPJ: 16,888,315/0001-57	14. Unit/Body: Office of the Vice-Rector for Research and Graduate Studies
15. Telephone: (38) 3532-1200	16. Other Telephone:	

Commitment Term (from the person responsible for the institution): I declare that I know and will comply with the requirements of CNS Resolution 466/12 and its Complements and as this institution has the conditions to develop this project, I authorize its execution.

Responsible: Thiago Fonseca Silva CPF: 073.774.676-90

Position/Function: Pro-Rector of Research and Postgraduate Studies

Date: 08 / 03 / 2022

Signature

MAIN SPONSOR

Machine Translated by Google

17. Name:	18. Telephone:	19. Other Telephone:
6797 Federal University of the Jequitinhonha Valleys and Mucuri	(38) 3532-1200	

Term of Commitment: I declare that I am aware of and will comply with the requirements of CNS Resolution 466/12 and its complementary provisions. I undertake to use the materials and data collected exclusively for the purposes set forth in the protocol and to publish the results, whether favorable or not.
I accept responsibility for the scientific conduct of the above project.

Name: ______________________ CPF: ______________________

Position/Function: ______________________ E-mail: ______________________

Date: ______ / ______ / ______

____
Signature

Research Project:
EFFECTIVENESS OF CRYOTHERAPY ON PAIN INTENSITY, DORSIFLEXION RANGE OF MOTION, EDEMA AND FUNCTION IN ACUTE ANKLE SPRAIN: A RANDOMIZED CONTROLLED TRIAL - THE FROST STUDY

Preliminary Information

Main Responsible

CPF/Document: 051.729.666-76	Name: VINICIUS CUNHA DE OLIVEIRA
Phone: 31995696214	Email: viniciuscunhaoliveira@yahoo.com.br

Proposing Institution

CNPJ: 16.888.315/0001-57	Name of Institution: Federal University of Jequitinhonha and Mucuri Valleys

Is this amendment submission exclusive to your Coordinating Center?

The amendment is exclusive to your Coordinating Center, so the changes made to your project, due to the amendment, will NOT be replicated in the Linked Participating Centers and the Ethics Committees of the Co-Participating Institutions, upon approval.

Is it an international study? No

Assistants

CPF/Document	Name
093.300.396-00	Julio Miranda

Study Area

Major Areas of Knowledge

- Major Area 4. Health Sciences

Main Purpose of the Study

- Clinical

Public Title of Research: EFFECTIVENESS OF CRYOTHERAPY ON PAIN INTENSITY, RANGE OF MOTION DORSIFLEXION, EDEMA AND FUNCTION IN ACUTE ANKLE SPRAIN: A CONTROLLED TRIAL RANDOMIZED - THE FROST STUDY

Acronym for Public Title: FROST

Acronym Expansion Freezing on Sprain Trial

Acronym FROST

Acronym Expansion: Freezing on Sprain Trial

Public Contact

CPF/Document	Name	Telephone	E-mail
051.729.666-76	VINICIUS CUNHA OF OLIVEIRA	31995696214	viniciuscunhaoliveira@yahoo.com.br

Scientific Contact: VINICIUS CUNHA OF OLIVEIRA

Study Design / Financial Support

Study Design: Intervention/Experimental

Health conditions or problems

Health Condition or Problem
acute ankle sprain

General Descriptors for Health Conditions

ICD1-10: International Classification of Diseases

ICD Code	CID Description
S93	Dislocation, sprain and strain of joints and ligaments at the ankle and foot

DeCS: Health Science Descriptors

DECS Code	DECS Description
C26.558.100	Ankle Injuries

Specific Descriptors for Conditions of

ICD1-10: International Classification of Diseases

ICD Code	CID Description
S93	Dislocation, sprain and strain of joints and ligaments at the ankle and foot

DeCS: Health Science Descriptors

DECS Code	DECS Description
C26.558.100	Ankle Injuries

Type of Intervention: Experimental

Nature of Intervention

- Other thermotherapy

Intervention Descriptors

Intervention Descriptors

Interventions
Cryotherapy

CID List

ICD Code	CID Description
S93	Dislocation, sprain and strain of joints and ligaments at the ankle and foot

List of DECS

DECS Code	DECS Description
E02.258	Cryotherapy

Phase

- Phase 4

Design:

Randomized controlled trial

Financial Support

CNPJ	Name	E-mail	Telephone	Type
16,888,315/0001-57 Federa	University of the Valleys Jequitinhonha and Mucuri		3835321200	Main Institutional

Keyword

Keyword
acute ankle sprain
Cryotherapy

Study Details

Summary:

The aim of this randomized controlled trial is to investigate the effectiveness of cryotherapy on function, pain intensity, edema, and dorsiflexion range of motion in people with an acute episode of ankle sprain. This is a two-arm randomized controlled trial protocol. People over 18 years of age with a clinical diagnosis of grade I or II ankle sprain, and up to 72 hours from the injury episode, will be randomly allocated to the Ice Group, which consists of a physician's prescription for home application of ice packs to the ankle with elevation, plus a nonsteroidal anti-inflammatory drug, or the No Ice Group, which consists of the same physician's prescription as the experimental group, but without ice included. Our primary outcome is function, measured by the Lower Extremity Functional Scale (LEFS) questionnaire.

Our secondary outcomes are pain intensity (Numerical Pain Scale, 0–10), edema (figure-of-eight method), and dorsiflexion range of motion (goniometry). Follow-ups will be performed at post-treatment (7–14 days) and 12 weeks after allocation. A sample size of 82 participants will be required for a minimum detection of the effect size of the primary outcome, with a power of 80%, a of 5%, and an expected dropout rate of 20%. Analyses will follow the intention-to-treat principle. Treatment effects will be analyzed using Linear Mixed Models. The results of this study may help clarify the effects of cryotherapy in the treatment of acute ankle sprain and may guide clinicians in decision-making.

Introduction:

Ankle sprain is a common condition in the general population, with a prevalence of 11.88% and an incidence of seven sprains per 1000 exposures in athletes, with sports-related ankle-foot injuries leading to the most demand for health services (GRIBBLE et al., 2016; DOHERTY et al., 2014; NABIAN et al., 2017). After a new episode of ankle sprain, there is a high frequency of chronic ankle instability and recurrence (GRIBBLE et al., 2016). Therefore, it is a musculoskeletal injury that can cause direct costs (expenses for medical consultations and medications) and indirect costs (absence from work and reduced productivity) (GRIBBLE et al., 2016). Therefore, effective therapies for the treatment of acute ankle sprains are increasingly sought by physicians. Treatment options frequently used after an ankle sprain include cryotherapy (BLEAKLEY; MCDONOUGH; MACAULEY, 2006; VAN DIJK, 1999), surgical treatment (DOHERTY et al., 2017), joint mobilization (COSBY et al., 2011), kinesiotherapy (BLEAKLEY et al., 2010; CLELAND et al., 2013), orthoses (BEYNNON et al., 2006), acupuncture (DOHERTY et al., 2017), and others. Cryotherapy is a low-cost and easy-to-use treatment option and has been recommended by clinical practice guidelines for use by health care professionals in acute ankle sprains (VAN DIJK, 1999; VUURBERG et al., 2018). It is considered a potentially effective therapy in the acute inflammatory phase due to the findings of preclinical research, which suggest that cryotherapy can control inflammatory processes by reducing macrophage infiltration and the levels of TNF-¿, NF-¿B, TGF-ß and MMP-9 mRNA (NEMET et al., 2009; VIEIRA RAMOS et al., 2016); in addition to promoting local analgesia by decreasing nerve conduction velocity (ALGAFLY; GEORGE, 2007), which could lead to improved clinical outcomes. However, current clinical research evidence supporting cryotherapy is still unclear. A previous systematic review (MIRANDA; SILVA; SILVA; MASCARENHAS; OLIVEIRA, 2021) investigated the efficacy of cryotherapy in acute ankle sprains and found that the literature lacks evidence to support its use, raising the importance of new randomized clinical trials with low risk of bias and with appropriate comparator groups to isolate the effects of therapies (i.e., placebo, sham, or no intervention) or to investigate whether cryotherapy enhances the effects of another intervention (i.e., cryotherapy combined with an active intervention compared with the same active intervention alone). Considering this, the objective of this randomized controlled trial is to investigate the efficacy of cryotherapy on function, pain intensity, edema, and dorsiflexion range of motion in people with an acute episode of ankle sprain.

Hypothesis:

3.1. Null hypothesis: There is no difference in the effects of treatment between the group with ice application compared to the group without ice application on pain intensity, dorsiflexion range of motion, edema and function in people with acute ankle sprain.3.2. Alternative hypothesis: There is a difference in the effects of treatment between the group with ice application compared to the group without ice application on pain intensity, dorsiflexion range of motion, edema and function in people with acute ankle sprain.

Primary Objective:

General Objective
To investigate the efficacy of cryotherapy on pain intensity, edema, dorsiflexion range of motion (DFROM) and function in people with acute ankle sprain.

Secondary Objective: 2.2

Specific Objectives ¿To
investigate the effectiveness of cryotherapy on pain intensity in people with acute ankle sprain USING THE NUMERICAL PAIN SCALE; ¿To investigate the effectiveness of cryotherapy on edema
in people with acute ankle sprain USING PERIMETRY USING THE FIGURE OF 8 TECHNIQUE; ¿To investigate the effectiveness of cryotherapy on DFROM in people with acute ankle sprain USING ACTIVE ANKLE
GONIOMETRY; ¿To investigate the effectiveness of cryotherapy on function in people with acute ankle sprain USING THE LOWER EXTREMITY FUNCTIONAL SCALE (LEFS) QUESTIONNAIRE

To investigate the adverse effects of cryotherapy in people with acute ankle sprain.

Proposed Methodology: This
is a two-arm prospective randomized controlled trial. A preliminary protocol was prepared following the recommendations of the SPIRIT guideline (CHAN et al., 2013) and will be registered with the Research Ethics Committee of the Federal University of the Jequitinhonha and Mucuri Valleys (UFVJM) and then registered on the REBEC website (www.ensaiosclinicos.gov.br). It will be reported according to the CONSORT statement (ELDRIDGE et al., 2016). All ethical principles provided by the Declaration of Helsinki (World Medical Association, 2013) will be followed by all members of this research throughout the study.

A qualitative analysis based on semi-structured interviews to investigate the barriers, facilitators, and beliefs of the participants will be carried out (Supplementary Material).

Inclusion Criteria:

Age between 18 and 60 years;
Clinical diagnosis of grade I or II ankle sprain, indicating an incomplete ligament rupture according to the classification of BIRRER et al. (1999); Time of up to a maximum of 72 hours from the injury episode
to the day of the medical consultation;

Machine Translated by Google

Bone fracture excluded by radiography or by the Ottawa rules for ankle (BACHMANN et al., 2003).

Exclusion Criteria:

Grade III (severe) ankle sprain, indicating complete ligament rupture, determined by a clear positive anterior drawer test and/or inversion stress test, accompanied by severe edema, hemorrhage, high level of pain on palpation, and total loss of DFROM and the ability to bear weight on the foot (BIRRER et al., 1999).

¿Open injury at the site, which contraindicates the application of ice; ¿Having
applied some form of cryotherapy more than once since the time of the injury; ¿Having any condition that
contraindicates the application of ice (for example, Reynaud's syndrome), or any other intervention prescribed in this study.

Risks:

The risks associated with this study are minimal, since this is a practice that is already widespread in the clinical setting. Participants in this study may be exposed to the risk of embarrassment and discomfort at the time of data collection, which will be minimized by performing the collections in separate rooms for this purpose, without the presence of any other individuals who are not directly involved in the data collection or who are not invited or accompanying the patients. Volunteers who receive cryotherapy may be exposed to peroneal nerve palsy, frostbite and/or allergic reactions to low temperatures. In the event of these adverse effects, volunteers will be instructed to stop treatment immediately and will be referred for a new consultation with the doctors on the team linked to the project, as would happen outside the context of this study, since the use of cryotherapy (ice) in the management of acute ankle sprains is widely used in the daily clinical practice of the institution. If clinically important favorable effects are found in favor of the application of cryotherapy, patients in the comparator group (group without ice) will be invited to receive physiotherapy treatment at the physiotherapy school clinic, in the physiotherapy department, at UFVJM.

Benefits:

The benefits of participating in the study include free daily monitoring by a physiotherapist, who will provide guidance on the management of the condition prescribed in this project, which can enhance the effects of the treatment and avoid possible adverse effects. In addition, the findings of this study will be of great relevance to clinicians and decision-makers in different areas, in addition to the development of new clinical guidelines for more efficient management of acute ankle sprains, promoting resolution and reducing long-term damage and consequent public spending.

Data Analysis Methodology: Statistical

analysis will be performed following the intention-to-treat principle. Data normality will be tested by the Kolmogorov-Smirnov test and data homoscedasticity by the Levene test. Parametric data will be expressed as mean and standard deviation and analyzed using Mixed Effects Models for repeated measures with Bonferroni post-hoc analysis for correction. In the case of nonparametric data, the median and its upper and lower limits will be expressed and analyzed using generalized linear mixed-effects models. All statistical analyses will be performed using the SPSS Statistics program (v.22.0; IBM Corp, Armonk, NY). Effect sizes will be interpreted based on their minimal clinically important differences (MCIDs).

Primary Outcome:

Function, measured with the 0-80 Lower Extremity Functional Scale (LEFS) questionnaire

Secondary Outcome:

Edema, measured using the figure-of-eight method, which consists of perimetry with a tape measure in the areas of greatest concentration of ankle edema (region of the anterior talofibular, calcaneofibular and anterior tibiofibular ligaments).

Pain intensity in the last 24 hours, measured with the Numerical Rating Scale (NRS), which consists of an 11-point scale, ranging from 0, which corresponds to "No pain" to 10, "Worst imaginable pain"

Active dorsiflexion range of motion, measured by active ankle goniometry, positioning the axis of the goniometer approximately 1.5 cm below the lateral malleolus, with the fixed arm aligned with the lateral midline of the leg and head of the fibula, and the mobile arm aligned with the fifth metatarsal. The participant will be instructed to perform as much dorsiflexion as possible

Sample Size in 82

Recruitment Countries

Country of Origin of the Study	Country	No. of survey participants
Yes	BRAZIL	82

Other Information

Will there be use of secondary data sources (medical records, demographic data, etc.)?

No

Report the number of individuals approached personally, recruited, or who will undergo some type of intervention at this center search:

82

Groups into which research participants at this center will be divided

Group ID	No. of Individuals	Interventions to be carried out
Ice Group	41	Ankle elevation, 3 days rest, NSAIDs and immersion in a bucket of ice the affected ankle
Iceless group	41	Ankle elevation, 3 days rest and NSAIDs

Is the Study Multicenter in Brazil?

No

Do you propose waiving the TCLE?

No

Will samples be retained for bank storage?

No

Execution Schedule

Stage Identification	Start (DD/MM/YYYY)	End Date (DD/MM/YYYY)
Reliability Pilot	03/28/2022	11/04/2022
Application of intervention	04/18/2022	01/01/2024
Summary of Results	01/02/2024	02/09/2024
Data collection	04/28/2022	11/01/2024
Recruitment of volunteers	04/18/2022	01/01/2024
Bibliographic update	03/28/2022	03/19/2024
Publication of results	02/19/2024	03/08/2024
Data analysis	01/22/2024	01/02/2024
Volunteer Allocation	04/18/2022	01/01/2024

Financial Budget

Budget Identification	Type	Value in Reais (R$)
Goniometers	Costing	R$ 200.00
Costs for calls and SMS to volunteers	Costing	R$ 611.88
Volunteer movement for reassessment	Costing	R$ 800.00
Copy of the TCLE	Costing	R$ 82.40
Printouts of questionnaires and scales	Costing	R$ 287.00
Volunteer records	Costing	R$ 164.00
Intervention diary	Costing	R$ 41.00
Tape measures	Costing	R$ 12.00
Envelopes	Costing	R$ 82.00
Posters for advertising	Costing	R$ 10.00
Carbon paper	Costing	R$ 164.00
Total in R$		R$ 2,454.28

Bibliography:

Machine Translated by Google

Algafly, A.; George, KP The effect of cryotherapy on nerve conduction velocity, pain threshold and pain tolerance. Br J Sports Med, v. 41(6), 365- 369, 2007. doi:10.1136/ bjsm.2006.031237 Bachmann, L.M., Kolb, E., Koller, M.T., Steurer, J., & ter Riet, G. Accuracy of Ottawa ankle rules to exclude fractures of the ankle and mid-foot: systematic review. Bmj, v. 326(7386), 417, 2003. doi:10.1136/bmj.326.7386.417 Beynnon, BD, Renström, PA, Haugh, L., Uh, BS, & Barker, H. A prospective, randomized clinical investigation of the treatment of first-time ankle sprains. Am J Sports Med, v.34(9), 1401-1412, 2006. doi:10.1177/0363546506288676 Binkley, JM, Stratford, PW, Lott, SA, & Riddle, DL The Lower Extremity Functional Scale (LEFS): scale development , measurement properties, and clinical application. North American Orthopedic Rehabilitation Research Network. Phys Ther, v.79(4), 371-383, 1999. Birrer, RB, Fani-Salek, MH, Totten, VY, Herman, LM, & Politi, V. Managing ankle injuries in the emergency department. J Emerg Med, 17(4), 651-660, 1999. doi:10.1016/s0736-4679(99)00060-8 Bleakley, CM, McDonough, SM, & MacAuley, DC Cryotherapy for acute ankle sprains: A randomized controlled study of two different icing protocols. British Journal of Sports Medicine, v.40(8), 700-705, 2006. doi:http:// dx.doi.org/10.1136/bjsm.2006.025932 Bleakley, CM, et al. Effect of accelerated rehabilitation on function after ankle sprain: Randomized controlled trial. BMJ (Online), v.340(7756), 1122, 2010. doi:http://dx.doi.org/10.1136/bmj.c1964 Chan, A. W, et al. SPIRIT 2013 explanation and elaboration: guidance for protocols of clinical trials. Bmj, v.346, e7586, 2013. doi:10.1136/bmj.e7586 Cleland, J.

A., Mintken, PE, McDevitt, A., Bieniek, ML, Carpenter, KJ, Kulp, K., Whitman, JM Manual physical therapy and exercise versus supervised home exercise in the management of patients with inversion ankle sprain: a multicenter randomized trial clinical trial. Journal of Orthopedic & Sports Physical Therapy, v.43(7), 443-455, 2013. doi:10.2519/jospt.2013.4792 Cosby, NL, Koroch, M., Grindstaff, TL, Parente, W., & Hertel, J .
Immediate effects of anterior to posterior talocrural joint mobilizations following acute lateral ankle sprain. J Man Manip Ther, v.19(2), 76-83, 2011. doi:10.1179/2042618610y.0000000005 Doherty, C., Bleakley, C., Delahunt, E., Holden, S. Treatment and prevention of acute and recurrent ankle sprain: an overview of systematic reviews with meta-analysis. Br J Sports Med, v.51(2), 113-125, 2017. doi:10.1136/bjsports-2016-096178 Doherty, C., Delahunt, E., Caulfield, B., Hertel, J., Ryan, J. ., & Bleakley, C. The incidence and prevalence of ankle sprain injury: a systematic review and meta-analysis of prospective epidemiological studies. Sports Medicine, v.44(1), 123-140, 2014. doi:10.1007/s40279-013-0102-5 Doig, GS, Simpson, F.
Randomization and allocation concealment: a practical guide for researchers. Journal of critical care, v.20(2), 187¿193, 2005. https://doi.org/ 10.1016/j.jcrc.2005.04.005 Eldridge, SM, Chan, CL, Campbell, MJ, Bond, CM , Hopewell, S., Thabane, L., Lancaster, GA
CONSORT 2010 statement: extension to randomized pilot and feasibility trials. Bmj, v.355, i5239, 2016. doi:10.1136/bmj.i5239 European Medicines Agency (EMA). Guideline on missing data in confirmatory clinical trials. 1¿12. [cited 2021 Nov 18], 2011. Available from: www.ema.europa.eu Gallagher, EJ, Liebman, M., Bijur, PE Prospective validation of clinically important changes in pain severity measured on a visual analog scale.
Ann Emerg Med, 38(6), 633-638, 2001. doi:10.1067/mem.2001.118863 Gogate, N., Satpute, K., Hall, T. The effectiveness of mobilization with movement on pain, balance and function following acute and sub acute inversion ankle sprain ¿ A randomized, placebo controlled trial. Physical Therapy in Sport, 2020. doi:10.1016/ j.ptsp.2020.12.016 Guirro, R., Abib, C., Máximo, C. The physiological effects of cryotherapy: a review.
Physiotherapy and Research, USP v.6(2), 164-170, 1999. doi: https://doi.org/10.1590/fpusp.v6i2.79629 Gribble, PA, Bleakley, CM, Caulfield, BM, Docherty, CL , Fourchet, F., Fong, DT, Delahunt, E. Evidence review for the 2016 International Ankle Consortium consensus statement on the prevalence, impact and long-term consequences of lateral ankle sprains. Br J Sports Med, v.50(24), 1496-1505, 2016. doi:10.1136/bjsports-2016- 096189 Hoffmann, TC, Glasziou, PP, Boutron, I., Milne, R., Perera, R., Moher, D., Altman, D. G., Barbour, V., Macdonald, H., Johnston, M., Lamb, SE, Dixon-Woods, M., McCulloch, P., Wyatt, JC, Chan, AW, & Michie, S. Better reporting of interventions: template for intervention description and replication (TIDieR) checklist and guide. BMJ (Clinical research ed.), v.348, g1687, 2014. https://doi.org/10.1136/bmj.g1687 Katz, J., & Melzack, R. Measurement of pain. Surg Clin North Am, v.79(2), 231-252, 1999. doi:10.1016/s0039-6109(05)70381-9 Kennet, J., Hardaker, N., Hobbs, S., Selfe, J. Cooling efficiency of 4 common cryotherapeutic agents. Journal of Athletic Training, v.42(3), 343, 2007. Mawdsley, RH, Hoy, D.

K., Erwin, PM Criterion-related validity of the figure-of-eight method of measuring ankle edema. Journal of Orthopedic & Sports Physical Therapy, v.30(3), 149-153, 2000. doi:10.2519/jospt.2000.30.3.149 Miranda, JP, Silva, WT, Silva, HJ, Mascarenhas, RO, Oliveira, VC Effectiveness of cryotherapy on pain intensity, swelling, range of motion, function and recurrence in acute ankle sprain: A systematic review of randomized controlled trials. Phys Ther Sport, v.49, 243-249, 2021. doi:10.1016/j.ptsp.2021.03.011 Mutlu, S., Y¿lmaz, E. The effect of soft tissue injury cold application duration on symptoms, edema, joint mobility, and patient satisfaction: a randomized controlled trial. Journal of emergency nursing, v.46(4), 449-459, 2020. doi: 10.1016/j.jen.2020.02.017 Nabian, MH, Zadegan, SA, Zanjani, LO, & Mehrpour, SR Epidemiology of Joint Dislocations and Ligamentous/Tendinous Injuries among 2,700 Patients: Five-year Trend of a Tertiary Center in Iran. Arch Bone Jt Surg, v.5(6), 426-434, 2017.
Nadler, SF, Weingand, K., Kruse, RJ The physiological basis and clinical applications of cryotherapy and thermotherapy for the pain practitioner.
Pain Physician, v.7, 395e399, 2004. Nemet, D., Meckel, Y., Bar-Sela, S., Zaldivar, F., Cooper, DM, & Eliakim, A. Effect of local cold-pack application on systemic anabolic and inflammatory response to sprint-interval training: a prospective comparative trial. Eur J Appl Physiol, v.107(4), 411-417, 2009. doi:10.1007/s00421-009-1138- y Pereira, LM, JM Dias, BF Mazuquin, LG Castanhas, MO Menacho, and JR Cardoso.
Translation, cross-cultural adaptation and evaluation of the psychometric properties of the Lower Extremity Functional Scale (LEFS): LEFS-Brazil [Dissertation]. Londrina: UniversidadeEstadual de Londrina, 2011. Rohner-Spengler, M., Mannion, AF, & Babst, R. Reliability and minimal detectable change for the figure-of- eight-20 method of, measurement of ankle edema. Journal of Orthopaedic & Sports Physical Therapy, v. 37(4), 199-205, 2007. doi:10.2519/jospt.2007.2371 Shepherd, JT, Rusch, NJ, & Vanhoutte, PM Effect of cold on the blood vessel wall. General Pharmacology: The Vascular System, v.14(1), 61-64, 1993. doi:10.1016/0306-3623(83)90064-2 Smith, TL, Curl, WW, George, C., & Rosencrance, E. Effects of contusion and cryotherapy on microvascular perfusion in rat dorsal skeletal muscle. Pathophysiology, vol. 1(4), 229-233, 1994. doi:10.1016/0928-4680(94)90002-7 van Dijk, CN [CBO-guideline for diagnosis and treatment of the acute ankle injury. National organization for quality assurance in hospitals]. Ned Tijdschr Geneeskd, v.143(42), 2097-2101, 1999. Vieira Ramos, G., Pinheiro, CM, Messa, SP, Delfino, G.
B., Marqueti Rde, C., Salvini Tde, F., Durigan, JL Cryotherapy Reduces Inflammatory Response Without Altering Muscle Regeneration Process and Extracellular Matrix Remodeling of Rat Muscle. Sci Rep, v. 6, 18525, 2016. doi:10.1038/srep18525 Vuurberg, G., Hoorntje, A., Wink, L.M., van der Doelen, BFW, van den Bekerom, M.P., Dekker, R., Kerkhoffs, G. Diagnosis, treatment and prevention of ankle sprains: update of an evidence-based clinical guideline. Br J Sports Med, v.52(15), 956, 2018. doi:10.1136/bjsports-2017-098106 Youdas, JW, McLean, TJ, Krause, DA, Hollman, JH Changes in active ankle dorsiflexion range of motion after acute inversion ankle spread. J Sport Rehabil, v.18(3), 358-374, 2009. doi:10.1123/jsr.18.3.358.

Upload Documents

File Attachments:

Type	File
Declaration of Institution and Infrastructure	letter_anuencia_CEP_DEPFISIOTERAPIA.pdf
TCLE / Terms of Assent / Justification of Absence	TCLE_modified.doc
Research Brochure	Supplementary_Material_Qualitative_Analysis.pdf
Title Page	signedFacesheet.pdf

Machine Translated by Google

Detailed Project / Researcher Brochure	DETAILED_FROST_PROJECT_MODIFIED.pdf
Receipt receipt	PB_COMPROVANTE_RECEPCAO_1905586.pdf
Declaration of Institution and Infrastructure	HNSS_Coparticipation_Letter.pdf
Others	LETTER_RESPONSE.pdf

Finish

Maintain confidentiality of the entire research project: Yes

Deadline: Until the publication of the results

Justification for the Amendment:

We chose qualitative analysis in this study due to the need for a deep understanding of the participants' perceptions and experiences regarding people's barriers, facilitators and beliefs who suffer ankle sprains and apply cryotherapy, due to its ability to explore in depth of individual experiences and perceptions. Barriers to the use of cryotherapy, such as lack of access to adequate resources, misinformation or fear of worsening the injury, can be identified and understood through interviews, allowing researchers to capture the nuances of the difficulties faced by patients. At the same time, qualitative analysis can reveal important facilitators, such as social support, guidance from health professionals and access to reliable information, which encourage adherence to treatment. In addition, this approach allows exploring the beliefs of patients about the effectiveness of cryotherapy, its perceived benefits, and any myths or biases that may influence its use. By capturing these perspectives directly from affected individuals. Therefore, qualitative analysis is crucial to obtain a holistic and contextualized view of these issues, providing rich, detailed data that quantitative methods might not capture as well depth. It is important to emphasize that the addition of qualitative analysis to the original project does not result in changes in any of the experimental methodological procedures, nor in increasing the size previously established sample.
