# Supplementary material for: Effects of cryotherapy on function, pain intensity, swelling, and dorsiflexion range of motion in acute ankle sprain: Protocol for the FROST randomised controlled trial
Source: PLoS One. 2025 Jun 4;20(6):e0325456. doi: 10.1371/journal.pone.0325456 (PMC12136321; doi:10.1371/journal.pone.0325456)
Supplement: S4 File — (DOCX) [file pone.0325456.s004.docx]

**SUPPLEMENTARY MATERIAL**

**INTERVENTION DIARY**

| **DIÁRIO DE APLICAÇÃO DO GELO** | | | |
| --- | --- | --- | --- |
| Caro voluntário, favor preencher as informações abaixo referente a data de aplicação do gelo, a hora em que foi aplicado e a duração. | | | |
| **Nome completo:** | | | |
| **Data** | **Hora da aplicação** | | **Duração da aplicação** |
| **____/_____/_______** |  | | **_________** minutos |
| **____/_____/_______** |  | | **_________** minutos |
| **____/_____/_______** |  | | **_________** minutos |
| **____/_____/_______** |  | | **_________** minutos |
| **____/_____/_______** |  | | **_________** minutos |
| **____/_____/_______** |  | | **_________** minutos |
| **____/_____/_______** |  | | **_________** minutos |
| **____/_____/_______** |  | | **_________** minutos |
| **____/_____/_______** |  | | **_________** minutos |
| **____/_____/_______** |  | | **_________** minutos |
| **____/_____/_______** |  | | **_________** minutos |
| **____/_____/_______** |  | | **_________** minutos |
| **____/_____/_______** |  | | **_________** minutos |
| **____/_____/_______** |  | | **_________** minutos |
| **____/_____/_______** |  | | **_________** minutos |
| **____/_____/_______** |  | | **_________** minutos |
| **____/_____/_______** |  | | **_________** minutos |
| **____/_____/_______** |  | | **_________** minutos |
| **____/_____/_______** |  | | **_________** minutos |
| **____/_____/_______** |  | | **_________** minutos |
| **Efeitos adversos da aplicação do gelo:** | | | |
| ( ) Não houve efeitos adversos | | ( ) Queimadura  ( ) Coceira ou alergia na pele  ( ) Perda de movimento do pé  ( ) Outro: _____________________________________ | |

**DIARY INTERVENTION – adapted to english version**

| **ICE APPLICATION DIARY** | | | |
| --- | --- | --- | --- |
| Dear volunteer, please fill in the information below regarding the date of application of the ice, the time it was applied and the duration. | | | |
| **Full name:** | | | |
| **Date** | **application time** | | **Application duration** |
| **____/_____/_______** |  | | **_________** minutes |
| **____/_____/_______** |  | | **_________** minutes |
| **____/_____/_______** |  | | **_________** minutes |
| **____/_____/_______** |  | | **_________** minutes |
| **____/_____/_______** |  | | **_________** minutes |
| **____/_____/_______** |  | | **_________** minutes |
| **____/_____/_______** |  | | **_________** minutes |
| **____/_____/_______** |  | | **_________** minutes |
| **____/_____/_______** |  | | **_________** minutes |
| **____/_____/_______** |  | | **_________** minutes |
| **____/_____/_______** |  | | **_________** minutes |
| **____/_____/_______** |  | | **_________** minutes |
| **____/_____/_______** |  | | **_________** minutes |
| **____/_____/_______** |  | | **_________** minutes |
| **____/_____/_______** |  | | **_________** minutes |
| **____/_____/_______** |  | | **_________** minutes |
| **____/_____/_______** |  | | **_________** minutes |
| **____/_____/_______** |  | | **_________** minutes |
| **____/_____/_______** |  | | **_________** minutes |
| **____/_____/_______** |  | | **_________** minutes |
| **____/_____/_______** |  | | **_________** minutes |
| **Adverse effects of ice application:** | | | |
| ( ) There were no adverse effects | | ( ) burn  ( ) Itchy or allergic skin  ( ) Loss of foot movement  ( ) Other: _____________________________________ | |
