# Supplementary material for: Effects of cryotherapy on function, pain intensity, swelling, and dorsiflexion range of motion in acute ankle sprain: Protocol for the FROST randomised controlled trial
Source: PLoS One. 2025 Jun 4;20(6):e0325456. doi: 10.1371/journal.pone.0325456 (PMC12136321; doi:10.1371/journal.pone.0325456)
Supplement: S5 File — (DOCX) [file pone.0325456.s005.docx]

**REGISTRO BRASILEIRO DE ENSAIOS CLÍNICOS (REBEC) REGISTRATION DATA SET**

| **Data category** | Information |
| --- | --- |
| Primary registry and trial identifying number | ensaiosclinicos.gov.br RBR-8v9gr9c |
| Date of registration in primary registry | 03 February, 2023 |
| Secondary identifying numbers | UTN number:  U1111-1285-1877  Plataformabrasil.saude.gov.br  58542222.2.0000.5108 |
| Source(s) of monetary or material support | Coordination for the Improvement of Higher Education Personnel (CAPES-BR) |
| Primary sponsor | Universidade Federal dos Vales do Jequitinhonha e Mucuri (UFVJM) |
| Secondary sponsor(s) | Nossa Senhora da Saúde Hospital |
| Contact for public queries | Full name: Julio Pascoal de Miranda  Address: Campus JK - Rodovia MGT 367 – Km 583, nº 5000. Bairro Alto da Jacuba,  City: Diamantina / Brazil  Zipe Code: 39100-000  Phone: +55 (38) 3532-1239  Email: julio.miranda@ufvjm.edu.br  Affiliation: Universidade Federal dos Vales do Jequitinhonha e Mucuri |
| Contact for scientific queries | Full name: Julio Pascoal de Miranda  Address: Campus JK - Rodovia MGT 367 – Km 583, nº 5000. Bairro Alto da Jacuba,  City: Diamantina / Brazil  Zip Code: 39100-000  Phone: +55 (38) 3532-1239  Email: julio.miranda@ufvjm.edu.br  Affiliation: Universidade Federal dos Vales do Jequitinhonha e Mucuri |
| Public title | Effectiveness of Ice application in acute ankle sprain treatment |
| Scientific title | Effectiveness of Cryotherapy on function, pain intensity, swelling, dorsiflexion range of motion in Acute Ankle Sprain: a randomized controlled trial - the FROST study |
| Countries of recruitment | Brazil |
| Health condition(s) or problem(s) studied | Acute Ankle Sprain |
| Intervention(s) | Experimental Group: home prescription to immerse the ankle on a bucket of ice and water until cover the area of swelling and pain, while sitting and positioning the affected limb with a 90° knee flexion, for up to 3 times a day for 20 minutes during 7 days with ankle elevation above the chest level in addition to non-steroidal anti-inflammatory medication (nimesulide 100 mg, 2 times a day, during 5 days) and medical advice to rest for 3 days. Additionally, the subjects will be individually instructed daily by a physiotherapist via phone calls and/or text messages and motivated to record the day and time of the ice applications using an intervention diary to assess the adherence and adverse effects. If the participant does not adhere to ankle immersion treatment, the physiotherapist will prescribe ice packs in the same dosage as an alternative cryotherapy option.  No Ice Group (Control): The subjects allocated to the ‘No Ice Group’ will receive the same interventions of the ‘Ice group’ but with no ice included. The prescription will be consisted of non-steroidal anti-inflammatory medication (nimesulide 100 mg, 2 times a day, during 5 days), ankle elevation above the chest level and medical advice to rest for 3 days. The subjects in the ‘No Ice Group’ will also be individually instructed daily by a physiotherapist through phone calls and/or text messages. Patients will be advised to not be part of any rehabilitation program until 12 weeks after allocation. In case of non-compliance with this orientation, we will perform sensitivity analysis to minimize the concomitant care effects. |
| Key inclusion and exclusion criteria | Inclusion criteria: Age between 18-60 years old; Clinical diagnosis of grade I or II ankle sprains, indicating an incomplete ligament rupture; Time of up to 72 hours from the episode of the injury to the day of the medical appointment; Bone fractures excluded by radiography or by the Ottawa ankle rules  Exclusion criteria: Grade III (severe) ankle sprain, indicating complete ligament injury, determined by a clear positive test of the anterior drawer and / or inversion stress test, accompanied by severe swelling, hemorrhage, high level of pain on palpation, in addition to total loss of the ability to support weight on the foot and of the dorsiflexion range of motion; Open injuries that contraindicate the application of ice; Having applied some form of cryotherapy more than once since the moment of the injury until the allocation process; Apply some form of cryotherapy after being assigned to the control group; Have any conditions that contraindicate the application of ice (e.g. Raynaud's syndrome), or any other intervention prescribed in this study. |
| Study type | Interventional Allocation: randomized Intervention model: two arm parallel assignment Masking: outcomes assessor Primary purpose: rehabilitation |
| Date of first enrolment | February 2023 |
| Target sample size | 82 |
| Recruitment status | Recruiting |
| Primary outcome(s) | Foot Function (0-80 Lower Extremity Functional Scale) |
| Key secondary outcomes | Active Ankle Dorsiflexion Range of Motion (Goniometry)  Ankle Swelling (Figure-of-eight perimetry method)  Pain intensity (0-10 Numerical Rating Scale) |
